# Photoswitchable Nitrogen Superbases: Using Light for Reversible Carbon Dioxide Capture

**DOI:** 10.1002/anie.202112344

**Published:** 2021-11-26

**Authors:** Lukas F. B. Wilm, Mowpriya Das, Daniel Janssen‐Müller, Christian Mück‐Lichtenfeld, Frank Glorius, Fabian Dielmann

**Affiliations:** ^1^ Institute of Inorganic and Analytical Chemistry Westfälische Wilhelms-Universität Münster Corrensstrasse 28–30 48149 Münster Germany; ^2^ Institute of General, Inorganic and Theoretical Chemistry Leopold-Franzens-Universität Innsbruck Innrain 80–82 6020 Innsbruck Austria; ^3^ Institute of Organic Chemistry Westfälische Wilhelms-Universität Münster Corrensstrasse 36 48149 Münster Germany

**Keywords:** CO_2_ activation, *N*-heterocyclic imines, nitrogen superbases, photochromism, photoswitches

## Abstract

Using light as an external stimulus to alter the reactivity of Lewis bases is an intriguing tool for controlling chemical reactions. Reversible photoreactions associated with pronounced reactivity changes are particularly valuable in this regard. We herein report the first photoswitchable nitrogen superbases based on guanidines equipped with a photochromic dithienylethene unit. The resulting N‐heterocyclic imines (NHIs) undergo reversible, near quantitative electrocyclic isomerization upon successive exposure to UV and visible irradiation, as demonstrated over multiple cycles. Switching between the ring‐opened and ring‐closed states is accompanied by substantial p*K*_a_ shifts of the NHIs by up to 8.7 units. Since only the ring‐closed isomers are sufficiently basic to activate CO_2_ via the formation of zwitterionic Lewis base adducts, cycling between the two isomeric states enables the light‐controlled capture and release of CO_2_.

## Graphical abstract

Light‐controlled CO_2_ activation was achieved by the development of photoswitchable nitrogen superbases equipped with a photochromic dithienylethene unit. The reversible electrocyclic isomerization of the *N*‐heterocyclic imine backbone is accompanied by substantial p*K*_a_ shifts of up to 8.7 units.

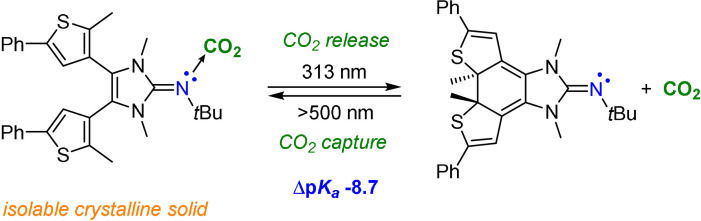

## Introduction

**Scheme 1:**
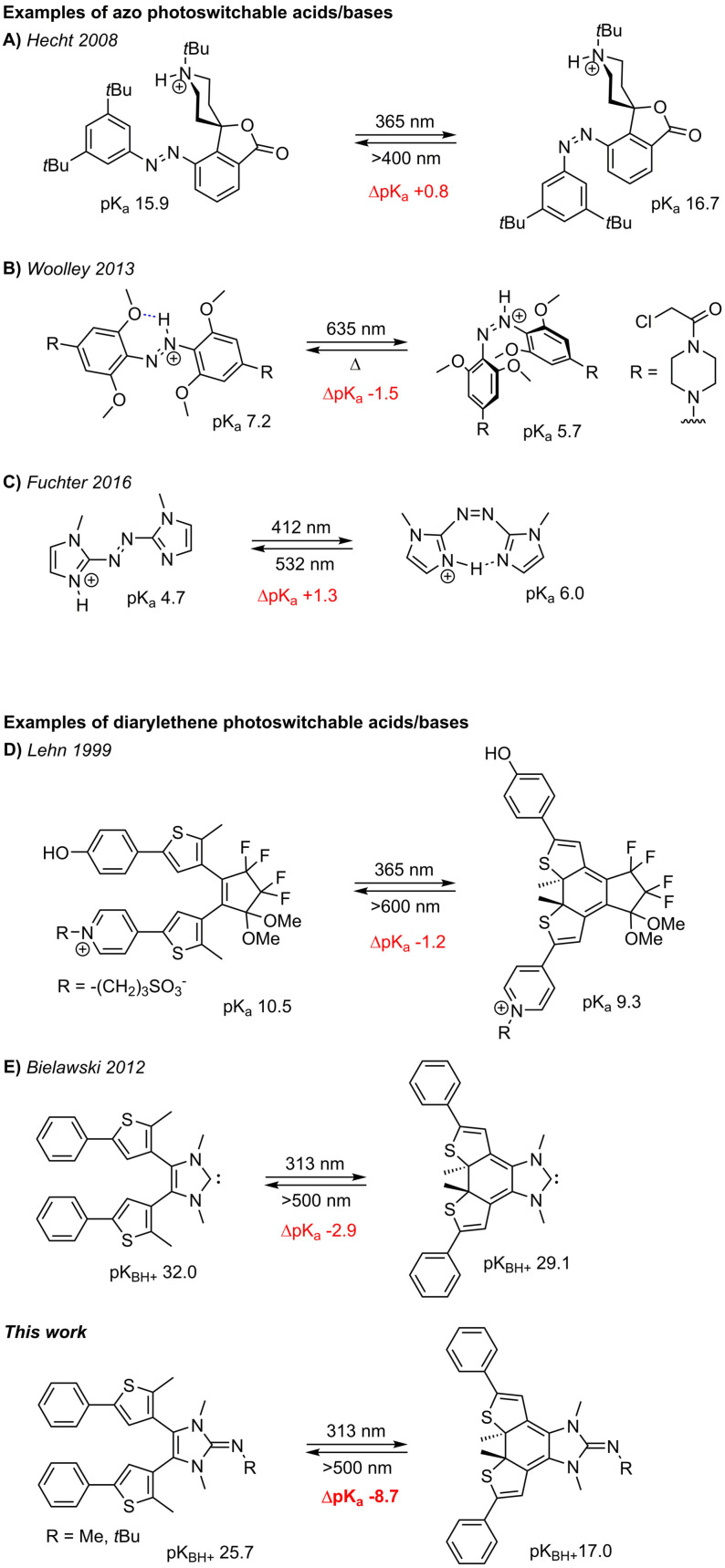
Examples for photoswitchable organic bases and their corresponding basicities [p*K*_a_ values determined in water (**B**, **C**), 5:2 methanol/water (**D**) and acetonitrile (**A**); p*K*_BH_^+^ values (**E**, this work {depicted for R=*t*Bu}) calculated using PW6B95‐D3//TPSS‐D3/def2‐TZVP + COSMO‐RS(acetonitrile)].

The effectiveness of light in initiating and regulating complex molecular and biochemical processes is evident, for example, in photoresponsive reactions such as photosynthesis^[1]^ or vision.^[2]^ Light is non‐invasive, provides excellent temporal and spatial control and can be precisely regulated with an appropriate light source. The remote‐control of chemical reactions using light is therefore particularly attractive.^[3]^ In this context, the development of photoswitchable acids and bases has attracted considerable attention,[^4^, ^5^, ^6^, ^7^, ^8^, ^9^, ^10^] as they enable external control of pH‐dependent chemical/biochemical processes or can act as photoswitchable catalysts.[^4^, ^11^] Apart from photoresponsive molecules, whose acidity/basicity is irreversible altered by the release of a caged proton upon irradiation with light,[^11^, ^12^] a common approach for photoswitchable bases and acids is based on the reversible *cis*‐*trans* photoisomerization of compounds containing azo[^4^, ^5^, ^7^, ^8^, ^9^, ^13^] or ethene groups[^6^, ^10^, ^14^] (Scheme 1). Between the two configurational isomers, p*K*_a_ shifts of up to 1.5 units were observed due to the stabilization of one isomer by hydrogen bonding interactions (Scheme 1 B,C) or on/off steric shielding of the basic site (Scheme 1 A). Another concept to modify basicity/acidity of chemical species takes advantage of the electronic changes that occur upon photocyclization of dithienylethene (DTE) derivatives. For example, Lehn and co‐workers showed that the acidity of phenol can be modulated by 1.2 p*K*_a_ units by photochemically establishing or disrupting the electronic communication with an electron‐withdrawing pyridinium group across a DTE backbone (Scheme 1 D). Branda and co‐workers showed that more significant reactivity changes were observed for DTE‐based switchable acids/bases when the central ethene unit is directly involved.^[15]^ An effective system was obtained by incorporating the photochromic DTE unit into the backbone of imidazolium salts.[^16^, ^17^, ^18^, ^19^, ^20^] The corresponding N‐heterocyclic carbenes (NHCs)[^21^, ^22^] function as photoswitchable ligands,^[23]^ organocatalysts[^24^, ^25^] or for reversible activation of ammonia.^[21]^

Recently, we have become interested in developing superbases capable of reversible CO_2_ capture via zwitterionic adducts.[^26^, ^27^] This kind of low‐energy bond formation is a promising way to activate and capture the relative inert CO_2_ molecule.^[28]^ Since nitrogen bases have the advantage of being less sensitive towards oxidation with molecular oxygen and hydrolysis than carbon or phosphorus bases, we recently explored the fixation of CO_2_ using N‐heterocyclic Imines (NHIs) and showed that both the basicity of NHIs and the CO_2_ binding energies are largely governed by the nature of the N‐heterocycle. Accordingly, Lewis base adducts between CO_2_ and NHIs with imidazoline backbone are sufficiently stable to be isolable, whereas the complexation of CO_2_ with benzimidazoline‐2‐imines is endergonic.^[27]^ We therefore envisaged to synthesize photoswitchable NHIs based on the NHC‐scaffold developed by Yam and Bielawski,[^16^, ^18^, ^19^, ^20^, ^25^] which will allow switching between these two states and thus enable light‐triggered reversible CO_2_ capture (Scheme 1).

Using (sun)light to drive the capture and release of CO_2_ is particularly attractive due to its abundance and facile usage. Photoresponsive systems capable of reversible CO_2_ uptake have been reported based on reversible structural changes of metal‐organic frameworks (MOFs),^[29]^ yet covalent CO_2_ binding with photoswitchable bases is unknown.

## Results and Discussion

**Scheme 2:**
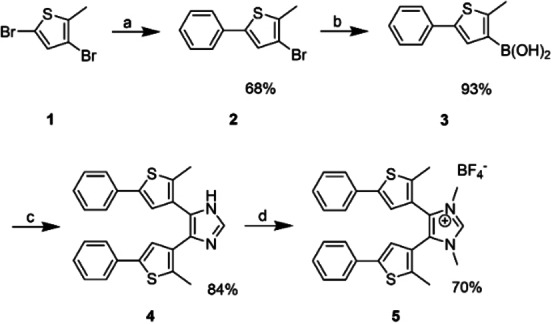
Synthesis of the imidazolium salt **5. a**: *n*BuLi, B(O^*n*^Bu)_3_, THF, −78 °C to RT, then Pd(PPh_3_)_4_, PhI, ethylene glycol, aq. Na_2_CO_3_, 80 °C, 16 h; **b**: *n*BuLi, B(O*n*Bu)_3_, THF, −78 °C to RT, then aq. HCl; **c**: Pd(PPh_3_)_4,_ 4,5‐diiodo‐2,5‐dihydro‐1*H*‐imidazole, K_2_HPO_4_, *n*Bu_4_NBr, MeOH‐H_2_O, 120°, 16 h; **d**: MeI, K_2_CO_3_, MeCN, 80 °C, 16 h, then NaBF_4_ in EtOH‐H_2_O.

The synthesis of photoswitchable imidazolium salts has been reported by Bielawski and co‐workers.[^19^, ^21^] However, since Bielwaski's original synthesis of the hexafluorophosphate derivative of **5** gives only a combined yield of 3.3 % in 7 steps from expensive 2‐methyl‐5‐phenylthiophene, we developed a new synthetic route by which the imidazolium salt **5** was synthesized in 5 steps and an overall yield of 32 % starting from more readily available 2‐methylthiophene (Scheme 2), which provided facile access to multiple grams of the imidazolium salt.

Following a literature procedure by Barbour and his co‐workers,^[30]^ 3,5‐dibromo‐2‐methylthiophene (**1**) was synthesized by bromination of 2‐methyl thiophene. The Suzuki coupling reaction between **1** and iodobenzene as coupling partner is selective and gave the desired 3‐bromo‐2‐methyl‐5‐phenylthiophene **2** in good yield.^[31]^ The boronic acid **3** was prepared in a one‐pot procedure with very good yield by subjecting **2** to lithium‐halogen exchange, followed by boronylation and hydrolysis of the in situ formed boronic esters. A modified and optimized reaction procedure^[32]^ was adopted for making the imidazole scaffold **4**. Two‐fold Suzuki coupling reaction between 4,5‐diiodoimidazole and the boronic acid **3** was performed to form the desired dithienyl imidazole derivative **4**. The final imidazolium salt **5** was obtained in good yield by methylation of **4** with iodomethane in MeCN,^[19]^ followed by a crystallization through anion exchange by addition of a sodium tetrafluoroborate solution (for detailed procedure see ESI).

**Scheme 3:**
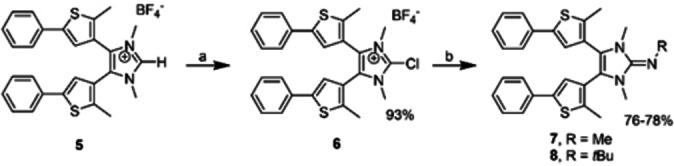
Synthesis of N‐heterocyclic imines **7** and **8. a**: NaHMDS, C_2_Cl_6_, THF, −78 °C; **b**: MeNH_3_Cl or *t*BuNH_2_, KF, MeCN, RT, 3 d, then KO*t*Bu, THF.

Following a protocol by Kunetskiy et al. for the synthesis of NHIs,^[33]^ we prepared the photoswitchable NHIs **7** and **8** starting from imidazolium salt **5** (Scheme 3). Deprotonation of **5** gave the corresponding free carbene which was treated with hexachloroethane to afford the 2‐chloroazolium salt **6** in very good yields. The KF‐mediated coupling of **6** with methylammonium chloride or *tert*‐butylamine in acetonitrile and subsequent deprotonation of the iminium salts (**7**HBF_4_ and **8**HBF_4_) gave the NHIs **7** and **8** as colorless solids in good yields. The NHIs **7** and **8** are soluble in common organic solvents including nonpolar media like *n*‐hexane or Et_2_O. The IR spectra show strong, characteristic absorption bands for the asymmetric ν_as_(C=N) (**7**: 1656 cm^−1^, **8**: 1685 cm^−1^) and ν_as_(C‐S) (**7**,**8**: 689 and 755 cm^−1^) stretching vibrations.

Both NHIs **7** and **8** undergo electrocyclic isomerization upon exposure to UV irradiation (λ_irr_=313 nm), while exposure to visible light (λ_irr_=520 nm) reversed the reaction. The NHIs can thus be switched reversibly between the open (**7 o**, **8 o**) and closed (**7 c**, **8 c**) form.

**Figure 1:**
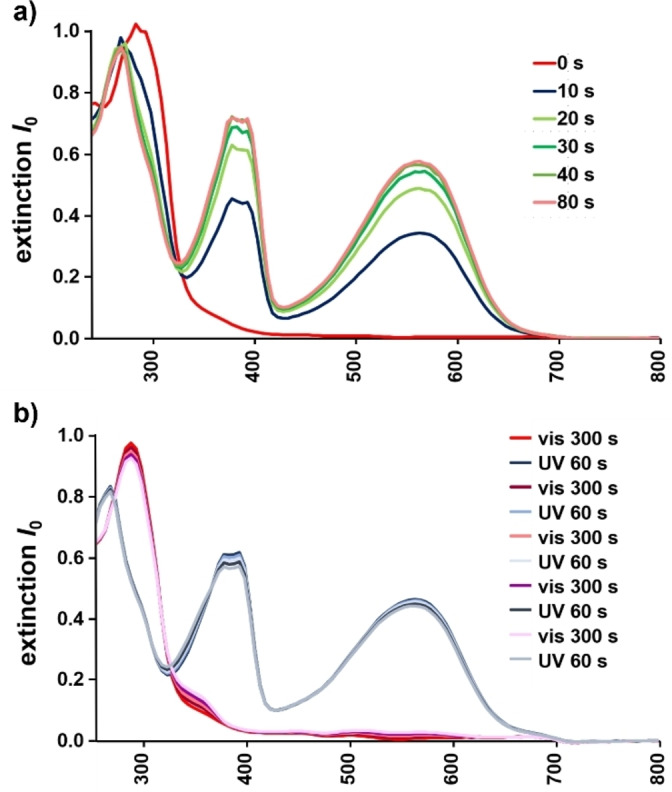
a) UV/Vis spectral changes of **7 o** upon UV irradiation (λ_irr_=313 nm). b) UV/Vis spectral changes of **7 o** upon successive UV (λ_irr_=313 nm, *t*=60 s) and visible‐light irradiation (λ_irr_=520 nm, *t*=300 s). Measured in MeCN ([**1 o**]=7.1×10^−4^ M).

The UV/Vis spectra recorded for **7 o** and **8 o** dissolved in MeCN (Figure 1) are very similar to those of Bielawski's photoswitchable NHCs.^[21]^ Intense absorption bands appear at 285 nm, which can be assigned to the n→π^*^‐ and π→π^*^ transitions of the N‐heterocycle and the thiophene system, respectively. Irradiation of a colorless solution of **7 o** or **8 o** with UV light (313 nm) resulted the development of an intensive purple color, as expected for an extended conjugated π system. Concomitantly, the intensity of the absorption bands at 285 nm decreased and two new bands appear in the UV/Vis spectra at 398 nm and 564 nm.^[34]^ Isosbestic points were detected at 337 nm, indicating that the photoisomerization proceeds without the formation of significant by‐products and is therefore stoichiometric.^[35]^ After 40 s of UV irradiation, the spectral changes subsided. Subsequent irradiation of the solutions containing **7 c** and **8 c** with visible light (520 nm) for 300 s resulted in colorless solutions, and the initial UV/Vis spectra were restored. For fatigue resistance testing, the solution of **7** and **8** were irradiated cyclically with UV and visible radiation. The UV/Vis spectra show only minimal changes when switching between the open (**7 o**) and closed state (**7 c**) over several cycles (Figure 1 B). Similar rapid switching and reversibility of photoisomerization was observed for imine **8** (Figure S39).

**Figure 2:**
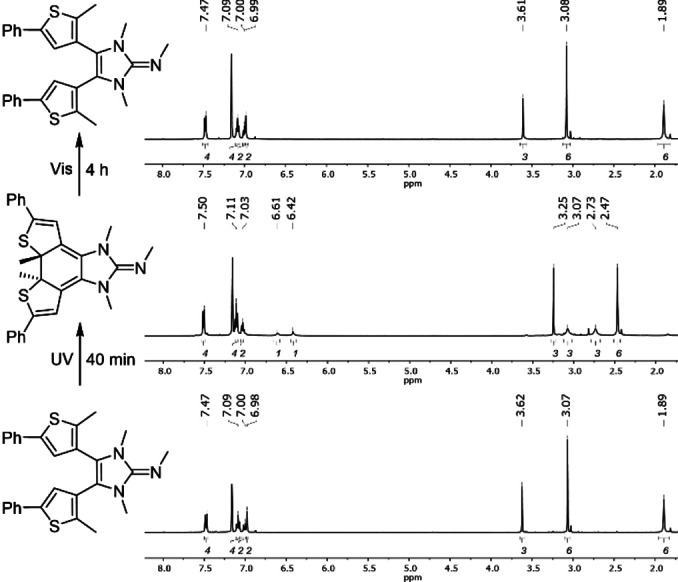
^1^H NMR spectra of **7 o** in C_6_D_6_ (bottom), of **7 c** after irradiation of the solution with UV light (313 nm) for 40 min (middle) and of **7 o** after further irradiation of the solution with visible light (500 nm) for 4 h (top).

The photoisomerizations were further confirmed by ^1^H NMR spectroscopy. In the ^1^H NMR spectrum of **7 o** in C_6_D_6_ the resonance for the thiophene CH protons is detected at 6.98 ppm and the resonance for the methyl groups at the endocyclic N atoms appears at 3.07 ppm (Figure 2). After irradiation of the NMR tube containing the solution at 313 nm for 40 min, the solution turns deep purple and the ^1^H NMR spectrum shows a new set of signals revealing the full conversion to **7 c**. The resonances assigned to the thiophene CH protons (6.61/6.42 ppm) and the methyl groups at the endocyclic N atoms (3.07/2.73 ppm) are no longer magnetically equivalent. In addition, a significant shift was observed for the proton signals of the exocyclic methyl group and the thiophene‐methyl groups from 3.62 ppm to 3.25 ppm and 1.89 ppm to 2.47 ppm, respectively. After exposure of the solution of **7 c** for 4 h with light at 500 nm, the ^1^H NMR spectrum of **7 o** was recovered. Similarly, the photoinduced cyclization of **8** was observed upon irradiation with UV and visible light, and in the ^1^H and ^13^C NMR spectra in MeCN‐*d*_3_ different signals for **8 o** and **8 c** were detected (Figure S38). As shown by the NMR experiments, photoswitching between the two states is almost quantitative. Moreover, the closed form is stable for months in the absence of light at room temperature.

**Figure 3:**
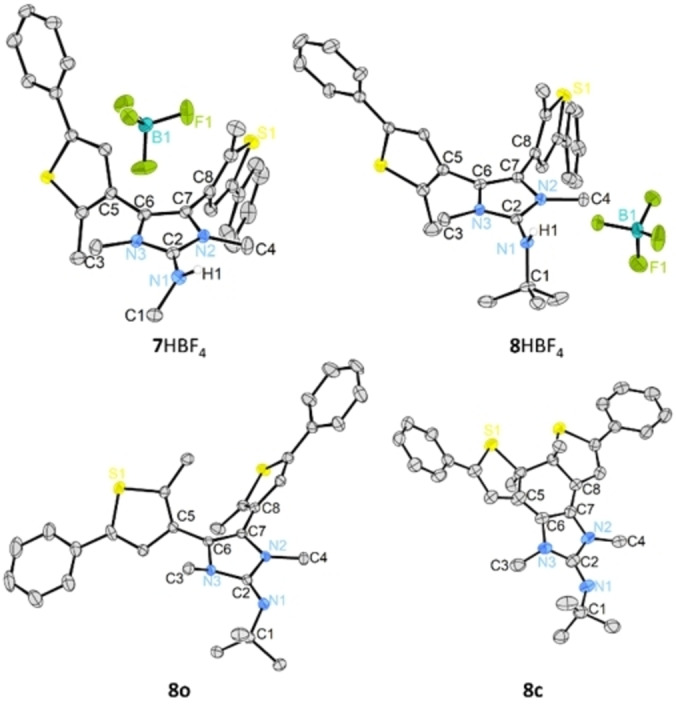
Molecular structure of **7**HBF_4_, **8**HBF_4_, **8 o** and **8 c** with ellipsoids set at 50 % probability. Hydrogen atoms except the NH‐atoms of **7**HBF_4_ and **8**HBF_4_ are omitted for clarity.

The molecular structures of **7**HBF_4_, **8**HBF_4_, **8 o** and **8 c** were established using single‐crystal X‐ray crystallography (Figure 3, Table 1).^[36]^ The thiophene rings of the open structures adopt antiparallel orientations and are almost orthogonal to the plane of the imidazole ring in the case of the iminium ions **7**HBF_4_ (67.8°/75.5°) and **8**HBF_4_ (87.3°/88.3°), while being more tilted towards the plane of the imidazole ring for the neutral NHI **8 o** (36.6°/56.7°). The guanidine unit of the iminium ions exhibit very similar C−N bond lengths (**7**HBF_4_: 1.339–1.356 Å; **8**HBF_4_: 1.344–1.375 Å) rendering the delocalization of the positive charge in the imidazole ring. In contrast, the exocyclic N1−C2 bond in the neutral NHI **8 o** (1.278 Å) is significantly shorter than the C2−N2 (1.384 Å) and C2−N3 (1.411 Å) bonds. This pronounced double bond character leads to steric strain between the methyl and the *tert*‐butyl substituents as indicated by the unusual pyramidalization of the N3 atom (sum of angles: 335.6°). Comparison of the molecular structures of **8**HBF_4_ and **8 o** corroborate the effect described by Kunetskiy that protonation of the imine N atom of NHIs leads to steric relaxation in the molecule and thus increased basicity of the NHIs.^[33]^

**Table 1:** Selected bond lengths [Å] and angles [°] of **7**HBF_4_, **8**HBF_4_, **8 o** and **8 c**.

Entry	**7**HBF_4_	**8**HBF_4_	**8 o**	**8 c**
N1‐C2	1.356(2)	1.375(3)	1.278(3)	1.282(7)
C2‐N2	1.342(2)	1.350(4)	1.384(3)	1.406(9)
C2‐N3	1.339(2)	1.344(3)	1.411(3)	1.379(8)
C1‐N1‐C2	122.17(14)	120.1(2)	127.5(2)	132.6(6)
N1‐C2‐N2	125.11(14)	127.4(3)	121.0(2)	118.1(6)
N1‐C2‐N3	126.6(2)	125.0(2)	134.0(2)	136.0(6)
N1‐C2‐N2‐C4	9.3(2)	1.7(5)	2.7(4)	5.6(9)
N1‐C2‐N3‐C3	6.1(3)	7.5(5)	43.5(5)	25.3(10)
C1‐N1‐C2‐N3	45.8(2)	83.8(4)	21.6(5)	9.9(10)
C5‐C6‐C7‐C8	167.6(3)	178.8(5)	0.7(6)	4.2(10)

Single crystals of **8 c** were obtained by storing a saturated acetonitrile solution in the absence of light. The molecular structure confirms that the two thiophene rings in **8 c** are connected via a C−C single bond of 1.533 Å. Changes in bond length and angles from **8 o** to **8 c** are consistent with the aforementioned photoisomerization reaction and formation of a delocalized π system. Most notably, the elongation and reduced double bond character of the C6−C7 bond (**8 c**: 1.444 Å, **8 o**: 1.352 Å) hampers the stabilization of a positive charge in the imidazole ring via formation of a 6π system, which should translate into a significantly lower basicity of the closed form compared to the open structure.

To elucidate the influence of the photocyclization reaction on the basicity, the gas phase basicity (GB), proton affinity (PA) and p*K*_BH_^+^(MeCN) values of **7 o**, **7 c**, **8 o**, **8 c** and of Bielawski's carbene (Figure 1 E) **NHC‐o** and **NHC‐c** were computed (Table 2). The predicted p*K*_BH_^+^ values were referenced to the experimental value of an NHI (see the SI for details).^[33]^ In line with our previous study, the substituent at the exocyclic nitrogen atom has a significant influence on the basicity of the NHI as shown by the increased dissociation constant of **8 o** compared to **7 o** by a factor of 200. Upon photocyclization to the closed form, both NHIs **7** and **8** exhibit a substantially lower basicity with p*K*_a_ shifts of 6.1 and 8.7 units, respectively. In comparison, the p*K*_a_ value of the corresponding photoswitchable NHC is reduced by only 2.9 units upon cyclization of the dithienylethene backbone.

**Table 2:** Calculated gas phase basicity (GB), proton affinity (PA) and predicted p*K*_a_ values of the corresponding protonated bases BH^+^ in CH_3_CN (p*K*_BH_^+^(MeCN)), using PW6B95‐D3//TPSS‐D3/def2‐TZVP + COSMO‐RS.

Compound	GB [kcal mol^−1^]	PA [kcal mol^−1^]	p*K*_BH_^+^(MeCN)
**7 o**	251.1	258.8	23.4
**7 c**	241.1	248.4	17.3
**8 o**	256.6	264.7	25.7
**8 c**	245.6	253.3	17.0
**NHC‐o**	263.8	271.4	32.0
**NHC‐c**	258.9	266.3	29.1

We previously showed that the minimum basicity of nitrogen bases required for the formation of isolable CO_2_ adducts is in the range of that of DBN (1,5‐Diazabicyclo[4.3.0]non‐5‐en, GB=249.5 kcal mol^−1^). The DBN‐CO_2_ adduct is stable in the solid state at 21 °C under an CO_2_ atmosphere but shows a fluxional behavior in solution.^[27]^ The two distinct electronic states of NHIs **7** and **8** could therefore be suitable for the photo‐triggered capture and release of CO_2_.

**Figure 4:**
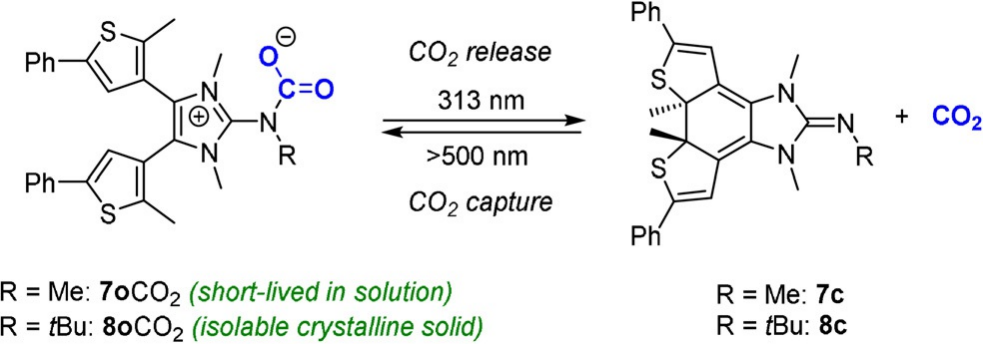
Light‐controlled CO_2_ activation.

To investigate the formation of NHI‐CO_2_ adducts, MeCN‐*d*_3_ solutions of **7 o** and **8 o** were pressurized with 2 bar ^13^C‐enriched ^13^CO_2_ under strictly anhydrous condition and analyzed by ^1^H and ^13^C NMR spectroscopy. Upon addition of ^13^CO_2_ to the acetonitrile solution of **7 o** the ^1^H NMR spectrum at 298 K shows slightly shifted signals for the nitrogen‐bound methyl groups from 3.13 and 3.28 to 3.21 and 3.49 ppm (Figure S36). However, the characteristic signal for the N‐CO_2_ unit in the range of 156 ppm^[27]^ was not detected in the ^13^C{^1^H} NMR spectrum. Instead, the ^13^C resonance of ^13^CO_2_ appeared as broad signal at 126.6 ppm slightly downfield to the chemical shift of free CO_2_ (125.9 ppm)^[37]^ indicating fluxional behavior involving short‐lived **7 o**CO_2_ complexes (Figure 4). A variable‐temperature NMR study revealed that the ^13^CO_2_ carbon resonance is sensitive to temperature and splits into two sharp signals at 154.4 ppm and 125.3 ppm that are unambiguously assigned to **7 o**CO_2_ and free CO_2_, respectively (Figure S37). An attempt to precipitate the CO_2_ adduct from a nonpolar solvent by exposing a solution of **7 o** in *n*‐hexane solution with 2 bar CO_2_ was unsuccessful. By contrast, exposing a solution of **8 o** in MeCN‐*d*_3_ with 2 bar ^13^CO_2_ leads to the quantitative formation of **8 o**CO_2_ as evident by a new set of signals in the ^1^H and ^13^C NMR spectrum including the indicative ^13^CO_2_ carbon resonance of the adduct **8 o**CO_2_ at 154.6 ppm (Figure S35).

Owing to the low solubility of **8 o**CO_2_ in nonpolar solvents, the adduct readily precipitates as white solid from Et_2_O solutions under an atmosphere of CO_2_. The decarboxylation temperature of **8 o**CO_2_ was estimated by heating the suspension in a closed system showing gas evolution at 70 °C concomitant with the formation of a clear solution. **8 o**CO_2_ can be stored in the absence of light under an argon atmosphere for months without noticeable decomposition. In the FT‐IR spectrum of **8 o**CO_2_, the CO_2_ stretching band ν_as_(CO) is detected at 1650 cm^−1^ consistent with that of other NHI‐CO_2_ adducts (1650–1673 cm^−1^).^[27]^

**Figure 5:**
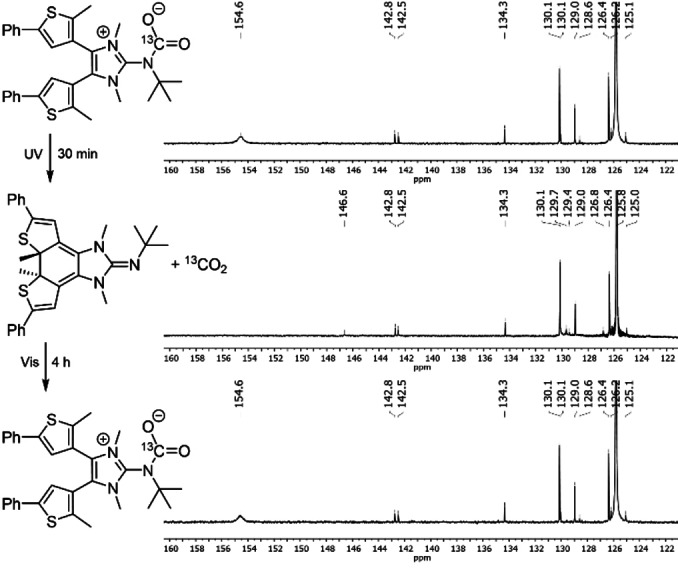
^13^C{^1^H} NMR spectra of **8CO_2_** in MeCN‐*d*_3_ (top), after irradiation of the NMR tube with UV light (313 nm) for 30 min showing the signals of **8 c** (middle), and after further irradiation with visible light (500 nm) for 4 h (bottom).

We next investigated the possibility of photo‐triggered capture and subsequent release of CO_2_ by utilizing the two distinct electronic states of **8** over the course of a single experiment (Figure 5). Using ^13^C‐enriched ^13^CO_2_, a solution of **8 o**CO_2_ in MeCN‐*d*_3_ was prepared in an NMR tube and sealed with a Teflon screw cap. The ^13^C{^1^H} NMR spectrum showed the characteristic ^13^CO_2_ signal of the adduct **8 o**CO_2_ at 154.6 ppm. Subsequently, the same solution was subjected to UV irradiation (313 nm) for 30 min, which resulted in a color change from colorless to dark purple. The ^13^C{^1^H} NMR spectrum of the purple solution showed the signal of free CO_2_ at 125.8 ppm, but no signal for the carboxylate carbon atom at 154.6 ppm was detected. Further visible‐light irradiation (500 nm) of the solution for 4 h resulted in decoloration of the solution and the ^13^C{^1^H} NMR spectrum matched that previously observed, confirming the reversibility of the photo‐driven CO_2_ capture and release process.

## Conclusion

We report the synthesis and properties of the first photoswitchable nitrogen superbases, namely N‐heterocyclic imines (NHI), outfitted with a photochromic dithienylethene unit. The NHIs **7 o** and **8 o** were found to undergo reversible photoinduced electrocyclization to their ring‐closed isomer **7 c** and **8 c** as evidenced by UV/Vis and NMR spectroscopy as well as XRD studies. The closed form is stable for months in the absence of light at room temperature. Successive exposure of the NHIs to UV (λ_irr_=313 nm) and visible (λ_irr_=520 nm) irradiation led to near quantitative switching between their ring‐opened and ring‐closed states over multiple cycles. Computational studies reveal that the changes in basicity between the two electronic states are significantly higher for NHIs (**7**: Δp*K*_a_=6.1, **8**: Δp*K*_a_=8.7) than for the corresponding NHC (Δp*K*_a_=2.9). This substantial p*K*_a_ difference provides new opportunities for light‐controlled chemical reactions as demonstrated by the reversible capture and release of CO_2_ using **8** over the course of a single experiment. Given the ubiquity of the guanidine function in many fields of research, access to photoswitchable NHIs that exhibit profound light‐triggered basicity alterations is expected to drive the development of new photoswitchable transformations.

## Conflict of interest

The authors declare no conflict of interest.

## Supporting information

As a service to our authors and readers, this journal provides supporting information supplied by the authors. Such materials are peer reviewed and may be re‐organized for online delivery, but are not copy‐edited or typeset. Technical support issues arising from supporting information (other than missing files) should be addressed to the authors.

Supporting Information

Supporting Information
