# Supplementary material for: Photoswitchable Nitrogen Superbases: Using Light for Reversible Carbon Dioxide Capture
Source: Angew Chem Int Ed Engl. 2021 Nov 26;61(3):e202112344. doi: 10.1002/anie.202112344 (PMC9299603; doi:10.1002/anie.202112344)
Supplement: Supplementary file 2 — Supporting Information [file ANIE-61-0-s002.pdf]

Angewandte Chemie
Eine Zeitschrift der Gesellschaft Deutscher Chemiker

Supporting Information

Photoswitchable Nitrogen Superbases: Using Light for Reversible Carbon Dioxide Capture

Lukas F. B. Wilm, Mowpriya Das, Daniel Janssen-Müller, Christian Mück-Lichtenfeld, Frank Glorius, and Fabian Dielmann**

anie_202112344_sm_miscellaneous_information.pdf
anie_202112344_sm_cif.zip

CONTENTS:

Synthetic Details 3

3,5-Dibromo-2-methylthiophene (1) 3

3-Bromo-2-methyl-5-phenylthiophene (2) 5

(2-Methyl-5-phenylthiophen-3-yl)boronic acid (3) 6

4,5-Bis(2-methyl-5-phenylthiophen-3-yl)-1H-imidazole (4) 8

1,3-Dimethyl-4,5-bis(2-methyl-5-phenylthiophen-3-yl)-1H-imidazol-3-ium tetrafluoroborate (5) 9

Preparation of 2-chloro-1,3-dimethyl-4,5-bis(2-methyl-5-phenylthiophen)imidazolium tetrafluoroborate (6) 12

Preparation of 7HBF4 15

Preparation of 8HBF4 18

Preparation of 7 21

Preparation of 8 22

Preparation of 8CO_2 23

Variable-Temperature NMR Study towards the formation of 7CO_2 25

Photoinduced cyclization of 8 26

UV/vis Spectroscopic Data 27

Photoswitchable CO2 activation 28

X-ray Diffraction Studies 29

Single-crystal X-ray structure analysis of 7HBF_4: 30

Single-crystal X-ray structure analysis of 8HBF_4 31

Single-crystal X-ray structure analysis of 8o 32

Single-crystal X-ray structure analysis of 8c 33

DFT Calculations 34

References 63

Synthetic Details

General remarks: All manipulations were performed under an inert atmosphere of dry argon, using standard Schlenk and drybox techniques. Dry and oxygen-free solvents were employed. ^{1}H, ^{13}C and ^{15}N spectra were recorded at 300 K on Bruker AVANCE I 400, Bruker AVANCE III 400 or Bruker AVANCE II 200 spectrometers. Low temperature NMR spectra were recorded on a Bruker AVANCE III 400 spectrometer at the temperatures indicated. All other spectra were obtained at 25 °C in the solvent indicated. Chemical shifts are given in parts per million (ppm) relative to $SiMe_4$ (^{1}H, ^{13}C) or NH_3 (^{15}N) and were referenced internally to the residual solvent signals. NMR multiplicities are abbreviated as follows: s = singlet, d = doublet, t = triplet, sept = septet, m = multiplet, br = broad signal. Mass spectra were obtained with an Orbitrap LTQ XL (Thermo Scientific) spectrometers IR spectra were obtained on a Bruker ALPHA II FT-IR Spectrometer. Carbon dioxide was purchased from Westfalen AG (Münster) as carbon dioxide 4.5 (99.995%). All other compounds were purchased from commercial sources.

3,5-Dibromo-2-methylthiophene **(1)**

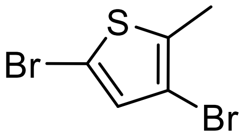

Following a known procedure[1] 2-methylthiophene (2.5g, 25.5mmol, 1.0 eq) was dissolved in acetic acid (50 mL). The mixture was cooled to 0 °C, then bromine (2.9 mL, 56.6 mmol, 2.2 eq) was added dropwise. The reaction was stirred at room temperature for overnight. The reaction was stopped by adding saturated sodium sulfite solution and water. The aqueous phase was extracted with diethyl ether. The collected organic phases were washed with saturated Na_2CO_3 solution. After drying over magnesium sulphate, the solvent was evaporated under reduce pressure. The resulting oil was filtrated through a plug of silica by using *n*-pentane. 3,5-dibromo-2-methylthiophene was isolated as a brown oil with a yield of 5.62 g (86%).

^{1}H NMR (400 MHz, $CDCl_3$) δ 6.85 (s, 1H), 2.34 (s, 3H).

^{13}C NMR (101 MHz, $CDCl_3$) δ 136.1, 132.1, 108.8, 108.6, 16.5, 15.0.

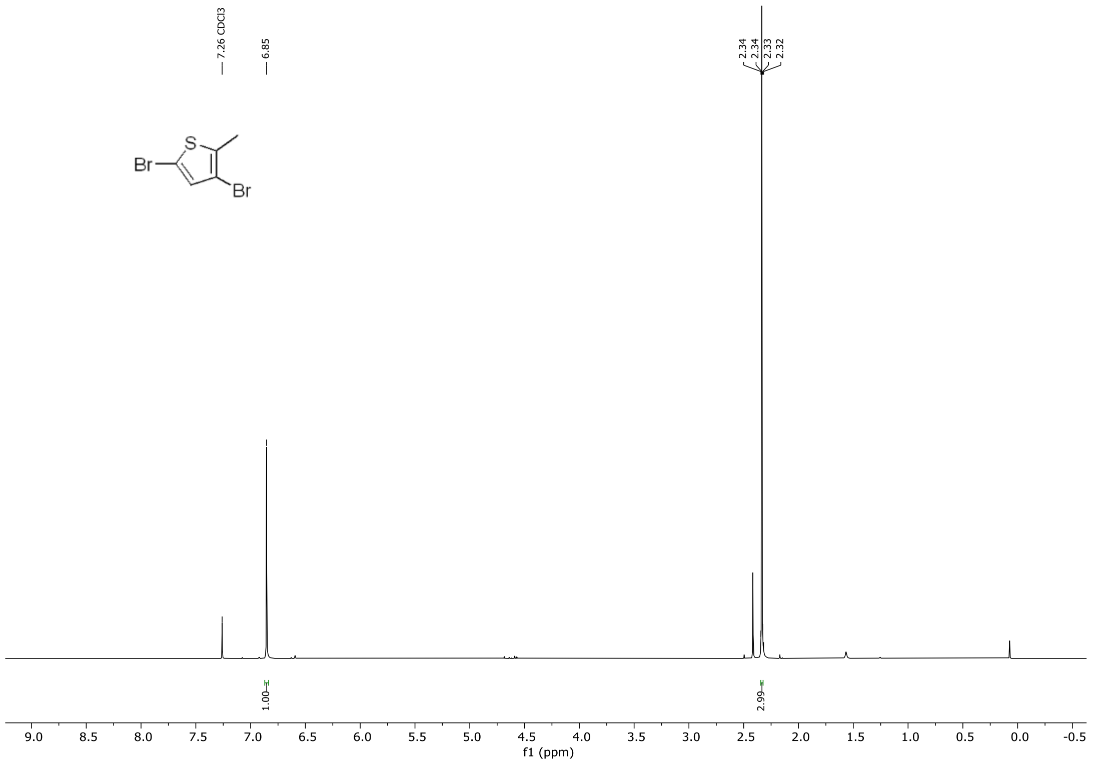

Figure S1: ^{1}H NMR spectrum (in $CDCl_3$, 400 MHz) of **1**.

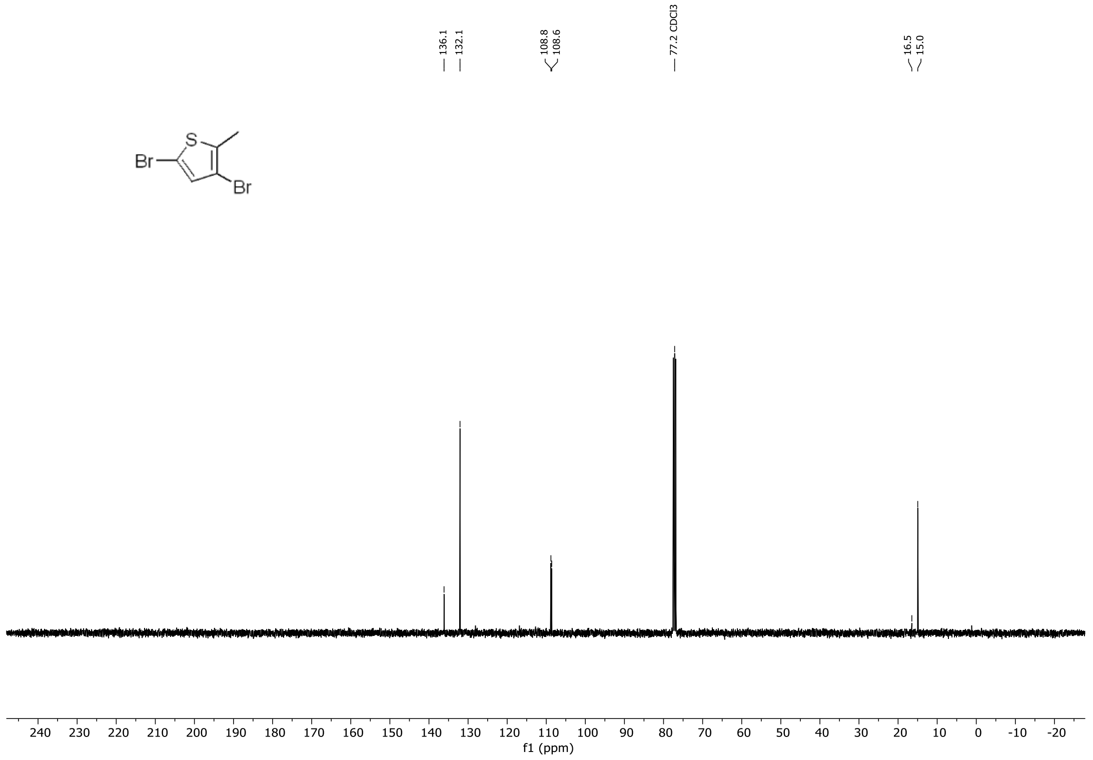

Figure S2: $^{13}C\{^{1}H\}$ NMR spectrum (in $CDCl_3$, 101 MHz) of **1**.

3-Bromo-2-methyl-5-phenylthiophene **(2)**

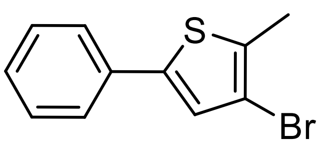

According to the procedure[2], a stirred solution of 3,5-dibromo-2-methylthiophene (3.7g, 14.4 mmol, 1.0 eq) in dry THF (40 mL) was cooled to –78 °C. n-BuLi (1.6 M solution in hexane, 10mL, 16 mmol, 1.1 eq) was added dropwise, after which stirring was continued for 30 min. Tributyl borate (4.2 mL, 15.9 mmol, 1.1 eq) was added dropwise and stirring was continued for 30 min, after which the reaction mixture was allowed to slowly warm up to room temperature. At room temperature, the mixture was stirred for 1 h. Meanwhile, in a separate flask, a stirred mixture of iodobenzene (4.8 mL, 43.1 mmol, 3.0 eq), $Pd(PPh_3)_4$ (0.23g, 0.20 mmol, 0.014 eq), ethylene glycol (1.1 mL), aqueous Na_2CO_3 (2 M, 37.0 mL), and THF (40 mL) was heated to 80 °C. The crude boronic ester from first flask was added via cannula and the resulting mixture was heated at 80 °C for 16 h. After cooling to room temperature, the mixture was diluted with 20mL of *n*-pentane. The organic phase was washed with water and then dried over magnesium sulfate. The solvent was evaporated under reduce pressure. The resulting oil was dissolved in n-pentane until all triphenylphosphine oxide has precipitated and the resulting suspension was filtered through a 5 cm plug of silica (n-pentane). The resulting solid was recrystallized from 20 mL MeOH and dried under high vacuum to afford 3-bromo-2-methyl-5-phenylthiophene as a white solid in a yield of 68% (2.50 g, 9.87 mmol). For some batches, column chromatography (SiO_2, pentane, Rf = 0.45) was used to further purify the product if the product was impure after recrystallization.

^{1}H NMR (400 MHz, $CDCl_3$) δ 7.53 – 7.49 (m, 2H), 7.40 – 7.34 (m, 2H), 7.32 – 7.26 (m, 1H), 7.11 (s, 1H), 2.42 (s, 3H).

^{13}C NMR (101 MHz, $CDCl_3$) δ 141.3, 133.8, 133.6, 129.1, 127.9, 125.7, 125.5, 110.0, 15.0.

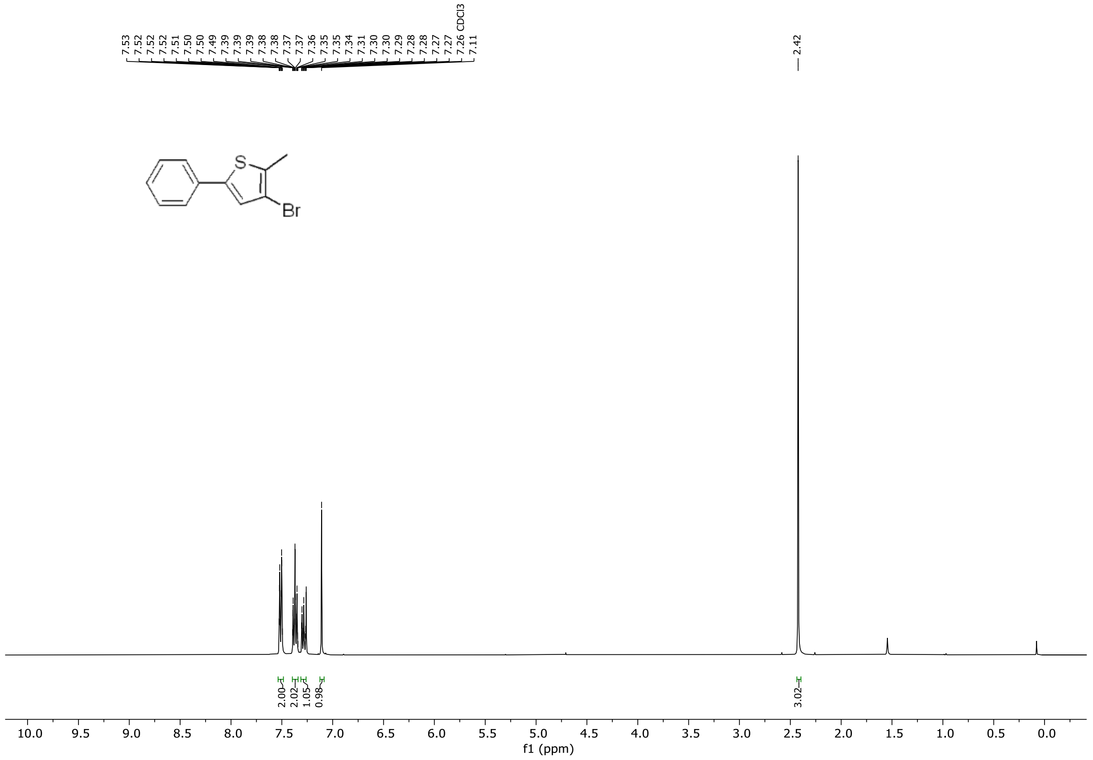

Figure S3: ^{1}H NMR spectrum (in $CDCl_3$, 400 MHz) of **2**.

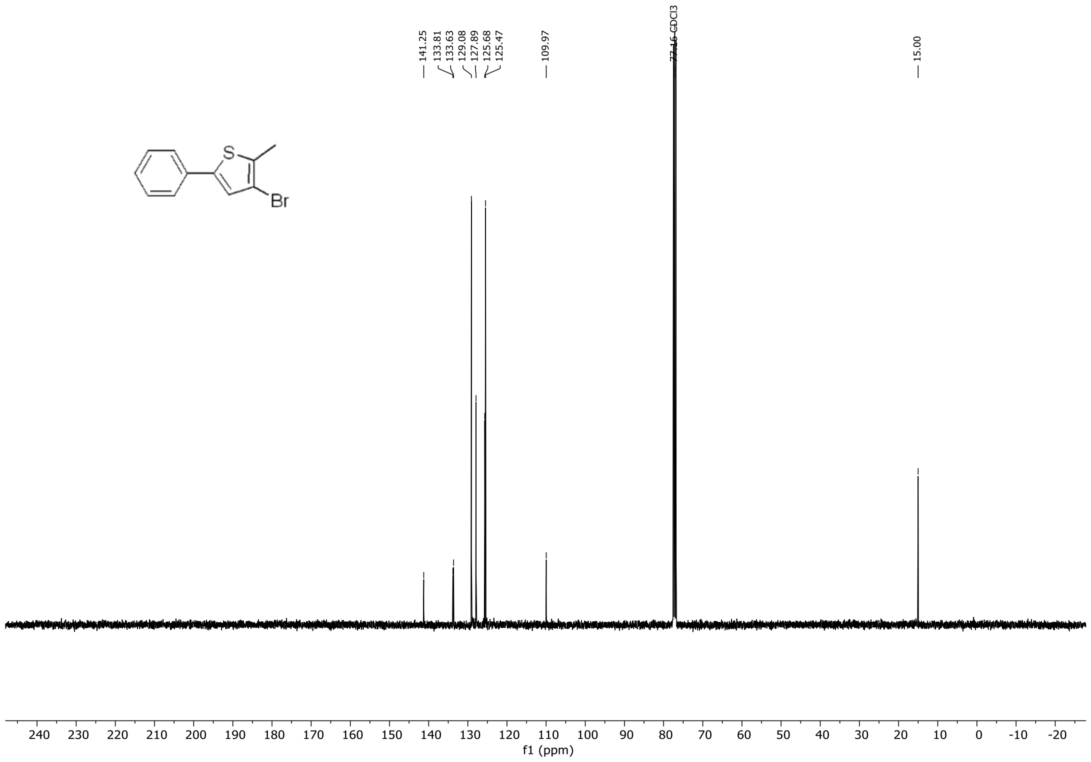

Figure S4: $^{13}C\{^{1}H\}$ NMR spectrum (in $CDCl_3$, 101 MHz) of **2**.

(2-Methyl-5-phenylthiophen-3-yl)boronic acid **(3)**

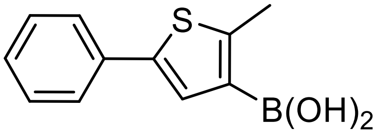

3-bromo-2-methyl-5-phenylthiophene (2.0 g, 7.9 mmol, 1 eq) was dissolved in THF (40 mL) and cooled down to -78 °C. n-BuLi (5.5 mL, 1.6 M solution in hexane, 8.7 mmol, 1.1 eq) was added dropwise. The reaction mixture was stirred for 1 h at -78 °C. Tributyl borate (2.76 mL, 10 mmol, 1.3 eq) was added rapidly, then the solution was stirred for 15 min at -78 °C. The reaction was warmed up to room temperature and was stirred for 30 min at room temperature. The reaction was stopped by adding HCl solution (20 mL, 1 M). The phases were separated and the organic phase was washed with 1 M NaOH solution. The collected aqueous phases were evaporated to remove the rest of THF. Then solution was acidified at pH 1 with 6 N HCl at 0 °C. The resulting solid was filtrated and washed with *n*-pentane. After drying the product was isolated as a white solid with a yield of 93% (1.6 g, 7.33 mmol).

^{1}H NMR (400 MHz, $CDCl_3$) δ 7.60 (s, 1H), 7.57 – 7.45 (m, 2H), 7.34 (m, 2H), 7.27 – 7.16 (m, 1H), 2.87 (s, 3H), 2.64 (s, 1H, one proton is missing probably due to deuterium exchange of hydroxyl proton).

^{13}C NMR (101 MHz, $CDCl_3$) δ 155.3, 140.9, 134.4, 129.6, 129.0, 129.0, 127.9, 127.4, 126.0, 125.8, 16.5.
HRMS (ESI): m/z calculated for $C_{11}H_{11}SBO_2Cl[M+Cl]^-$, 253.0268; found 253.0266.

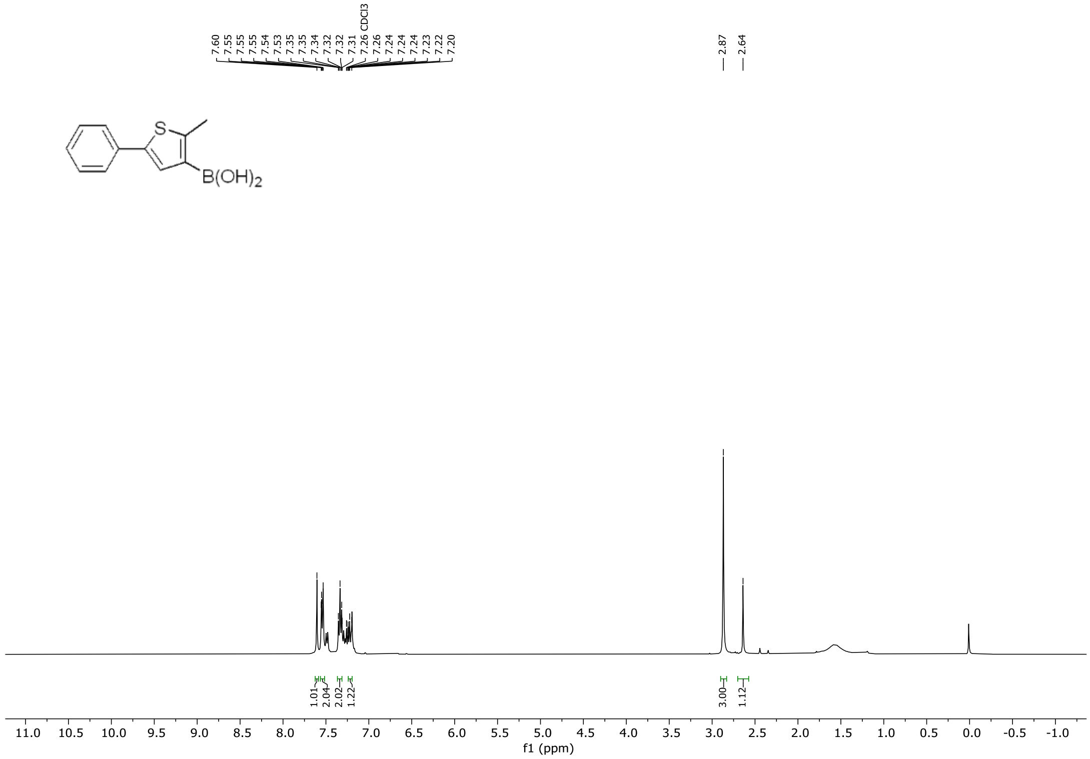

Figure S5: ^{1}H NMR spectrum (in $CDCl_3$, 400 MHz) of **3**.

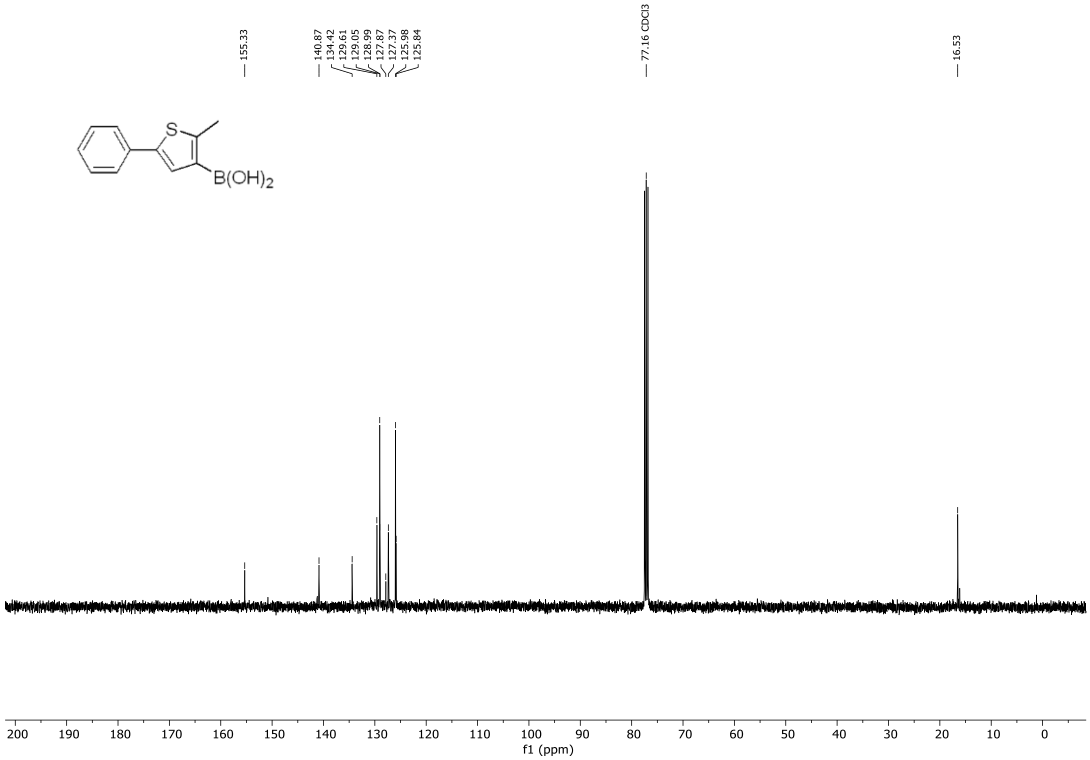

Figure S6: $^{13}C\{^{1}H\}$ NMR spectrum (in $CDCl_3$, 101 MHz) of **3**.

4,5-Bis(2-methyl-5-phenylthiophen-3-yl)-1H-imidazole **(4)**

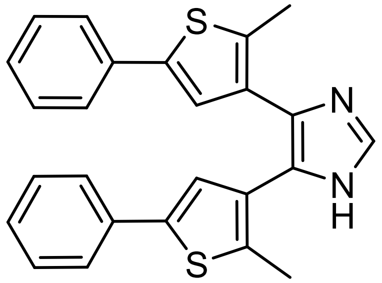

In analogy to a known procedure[3] (2-methyl-5-phenylthiophen-3-yl)boronic acid (1.31 g, 6.0 mmol, 3.0 eq.), 4,5-diiodo-2,5-dihydro-1H-imidazole (0.64 g, 2.0 mmol, 1.0 eq.), K_2HPO_4 (1.4 g, 8.0 mmol, 4.0 eq.), tetrabutyl ammonium bromide (64.5 mg, 0.2mmol, 0.1 eq.) and tetrakis(triphenylphosphine)palladium (138.7 mg, 0.12 mmol, 0.06 eq.) was dissolved in methanol (24 mL) and water (6 mL). The reaction was heated up to 120 °C and stirred overnight. After cooling down to room temperature the reaction was stopped by adding 20 mL water, 5 mL of 1 M sodium hydroxide solution and 40 mL DCM. The aqueous phase was extracted with a mixture of DCM and methanol (9:1). The collected organic phases were evaporated and DCM was added. The solution was stored in the fridge overnight for crystallization. The solid was filtrated and washed with *n*-pentane. After drying under vacuum the afford product was isolated as a yellow solid with a yield of 84% (690 mg, 1.67 mmol).

^{1}H NMR (300 MHz, CD_2Cl_2) δ 7.86 (s, 1H), 7.55 – 7.46 (m, 4H), 7.39 – 7.29 (m, 5H), 7.29 – 7.22 (m, 2H), 7.19 (s, 2H), 2.18 (s, 6H).

^{13}C NMR (101 MHz, CD_2Cl_2) δ 155.7, 141.0, 134.6, 129.9, 129.3, 129.3, 127.6, 127.5, 126.1, 125.9, 16.6.

HRMS (ESI): m/z calculated for $C_{27}H_{25}N_2S_2$ $[M+H]^+$, 413.1141; found 413.1142.

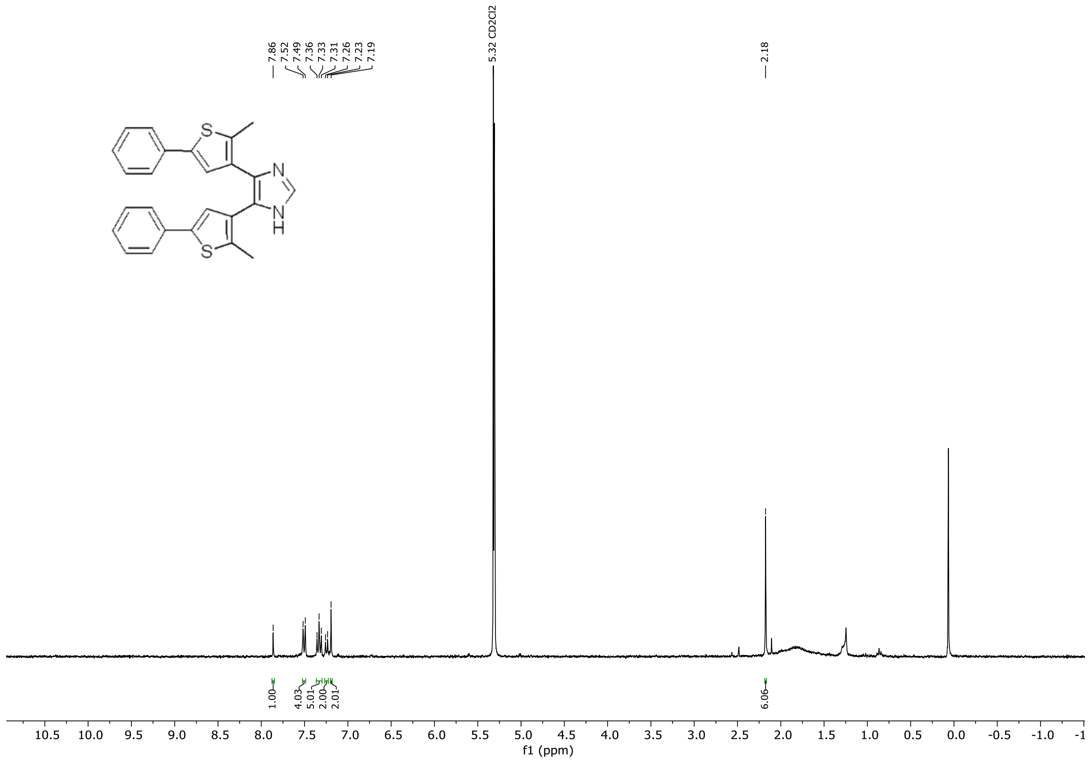

Figure S7: ^{1}H NMR spectrum (in CD_2Cl_2, 300 MHz) of **4**.

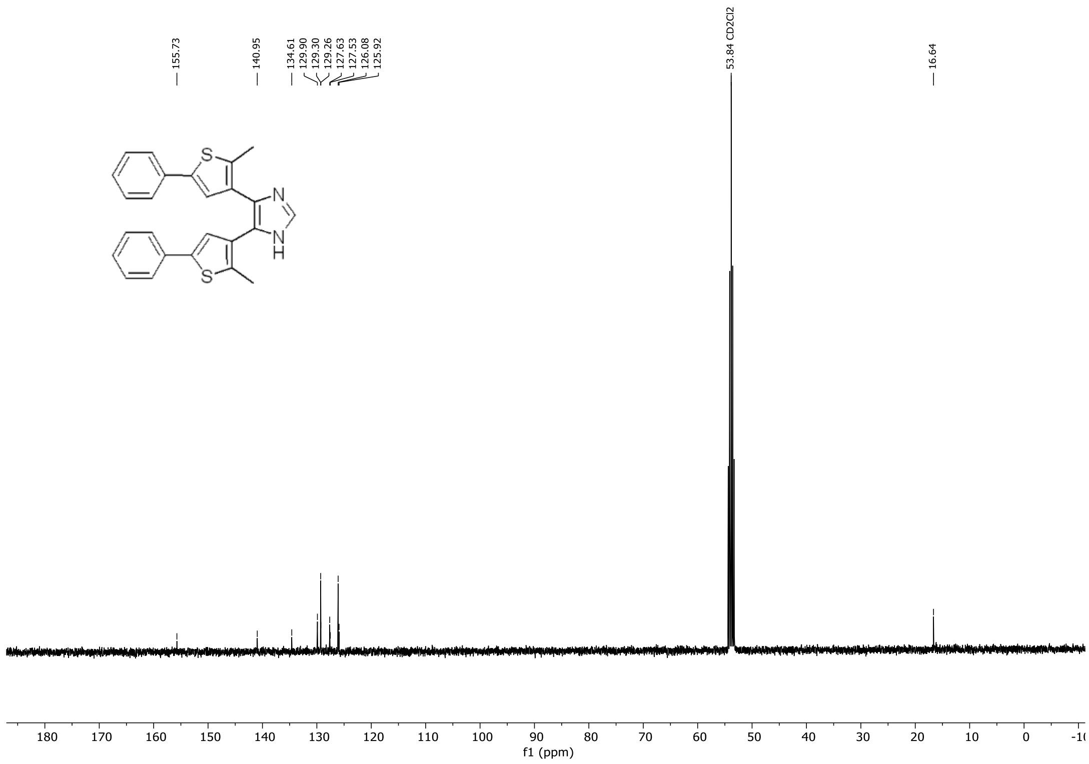

Figure S8: $^{13}C\{^1H\}$ NMR spectrum (in CD_2Cl_2, 101 MHz) of **4**.

1,3-Dimethyl-4,5-bis(2-methyl-5-phenylthiophen-3-yl)-1H-imidazol-3-ium tetrafluoroborate **(5)**

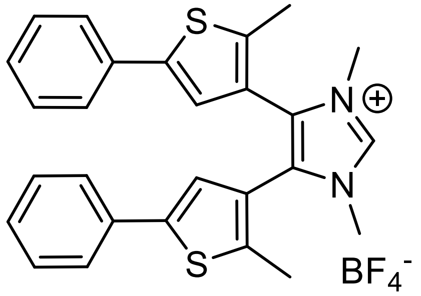

In analogy to a procedure by BIELAWSKI[4] 4,5-bis(2-methyl-5-phenylthiophen-3-yl)-2,5-dihydro-1H-imidazole (0.78 g, 1.9 mmol, 1.0 eq), methyl iodide (0.6 mL, 9.5 mmol, 5.0 eq) and potassium carbonate (0.97 g, 7.0 mmol, 3.7 eq) was dissolved in 50 mL acetonitrile. The reaction mixture was heated up to 80 °C and was stirred for 16 h. After cooling down to room temperature the mixture was filtrated over celite and the celite was flushed with DCM. The solvent was removed under reduce pressure and the resulting solid was taken up in a mixture of 20mL ethanol and 25mL water. The suspension was heated up to 80 °C and filtrated hot to remove solid impurities. A solution of sodium tetrafluoroborate (0.42 g, 3.8 mmol, 2.0 eq) in 1.2mL ethanol and 1.2mL water was added dropwise at 80 °C. After cooling down to room temperature the resulting solid was filtrated (if solid is not formed then cool it in ice bath) and washed with water an *n*-pentane. Then the solid was dissolved in DCM and dried using $MgSO_4$. After removing of the solvent and drying under reduce pressure the product was isolated as a white solid with a yield of 70% (700 mg, 1.32 mmol).

^{1}H NMR (400 MHz, DMSO-*d*$_6$) δ 2.06 (s, 6H), 3.81 (s, 6H), 7.27 – 7.37 (m, 2H), 7.38 – 7.47 (m, 4H), 7.59 – 7.66 (m, 6H), 9.41 (s, 1H).

^{13}C NMR (101 MHz, DMSO-*d*$_6$) δ 13.4, 34.2, 122.8, 124.3, 125.0, 127.1, 127.8, 129.0, 132.6, 137.5, 140.8, 141.0.

^{19}F NMR (377 MHz, DMSO-*d*$_6$) δ -148.4.

HRMS (ESI): m/z calculated for $C_{27}H_{25}N_2S_2$ [M-BF_4]$^+$, 441.1454; found 441.1468.

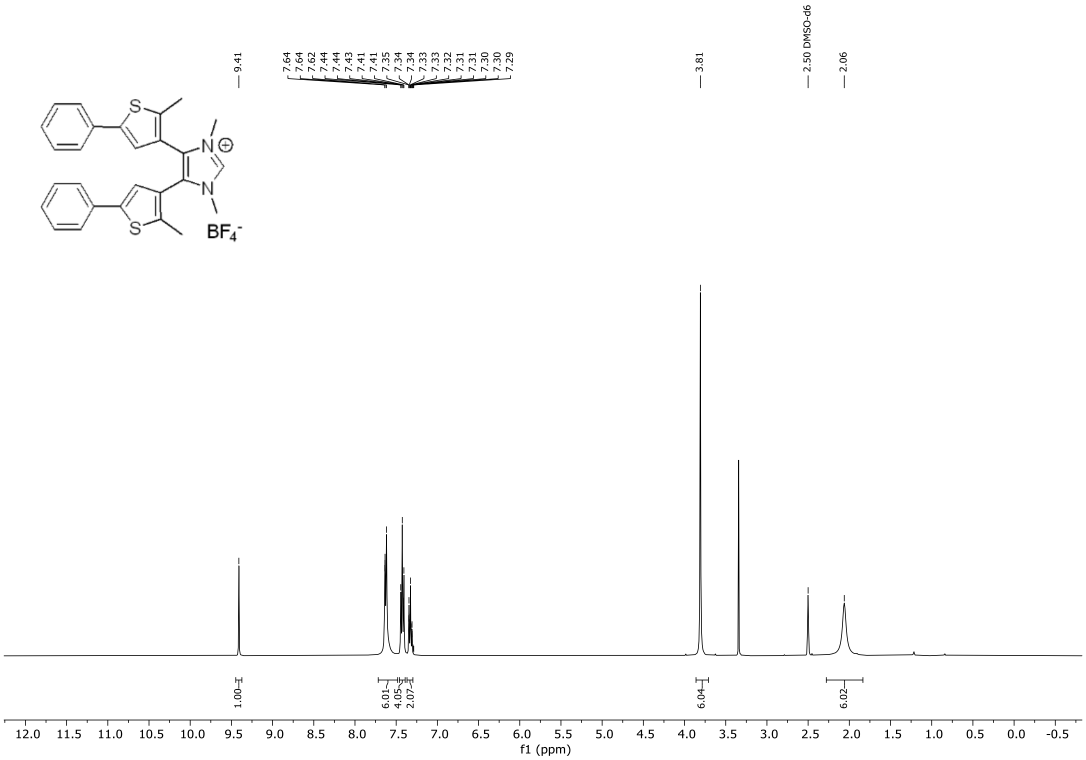

Figure S9: ^{1}H NMR spectrum (in DMSO-d$_6$, 400 MHz) of **5**.

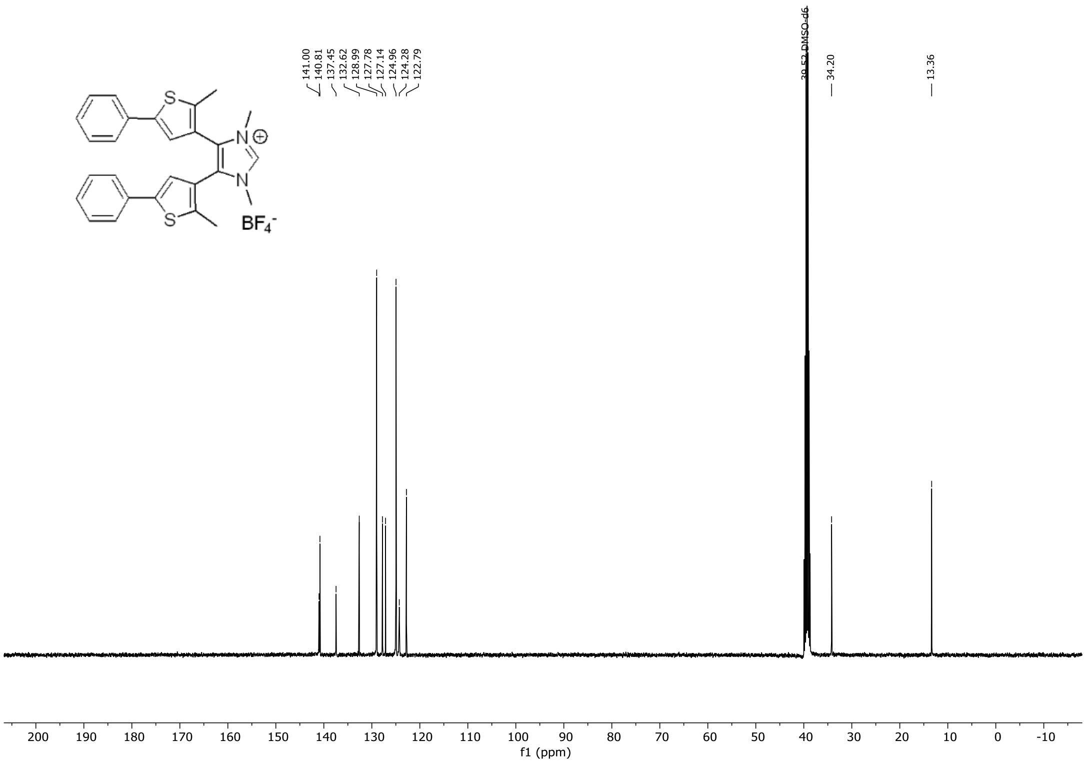

Figure S10: $^{13}C\{^{1}H\}$ NMR spectrum (in $CDCl_3$, 101 MHz) of **5**.

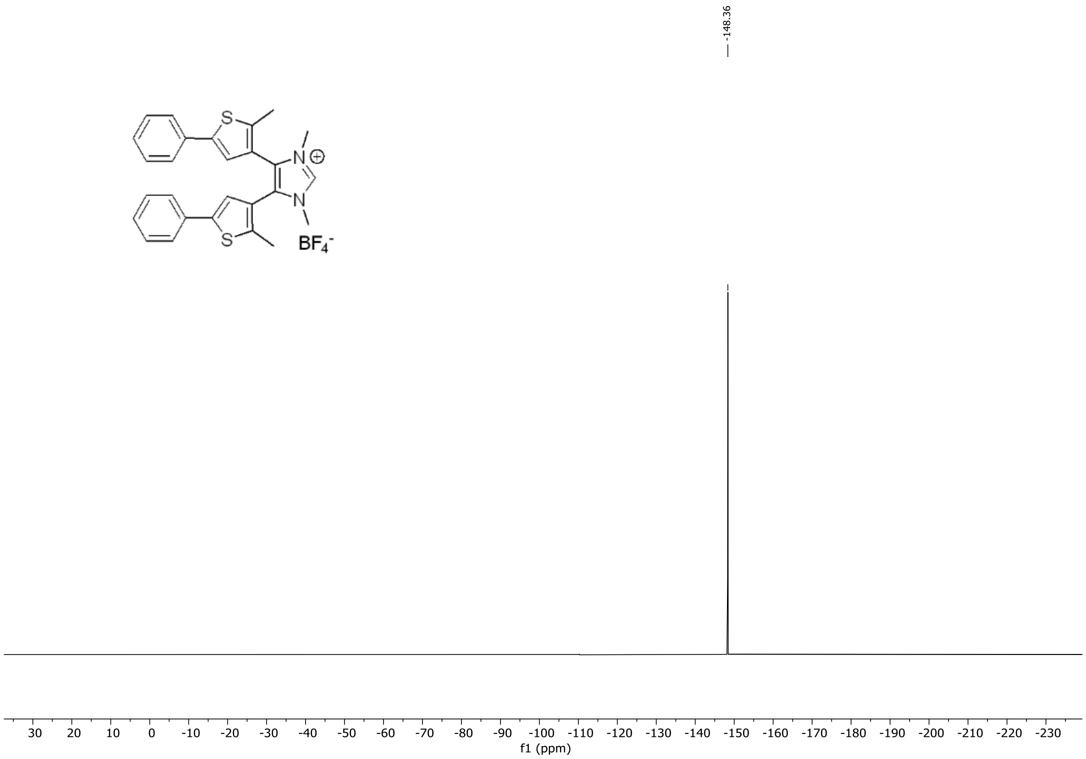

Figure S11: ^{19}F NMR spectrum (in DMSO-d_6, 377 MHz) of **5**.

Preparation of 2-chloro-1,3-dimethyl-4,5-bis(2-methyl-5-phenylthiophen)imidazolium tetrafluoroborate (6)

A suspension of 1,3-dimethyl-4,5-bis(2-methyl-5-phenylthiophene)imidazolium tetrafluoroborate (**5**) (700 mg, 1.32 mmol, 1.00 eq.) in THF (5 mL) was cooled to -78 °C and a solution of NaHMDS in THF (1.46 mL, 1.46 mmol, 1 M in THF, 1.10 eq.) was added dropwise. The reaction mixture was stirred at room temperature for 2 h and the resulting carbene solution was directly filtered to a cooled (–78 °C) solution of hexachloroethane (420 mg, 1.78 mmol, 1.34 eq.) in THF (5 mL). The resulting suspension was stirred at room temperature overnight, toluene was added (30 mL) and the white precipitate was filtered off. The solid was dried under reduced pressure, suspended in $CHCl_3$ and $NaBF_4$ (435 mg, 3.96 mmol, 3.00 eq.) was added. The volatiles were removed under reduced pressure and the solid was dried in high vacuum at 80 °C overnight yielding **6** as a white solid 93% (692 mg, 1.23 mmol).

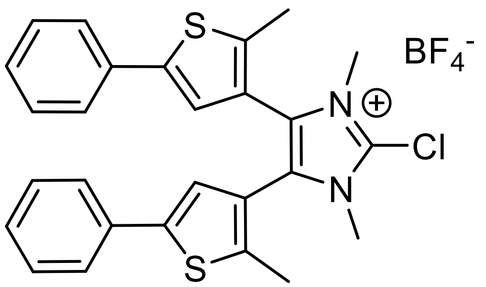

^{1}H-NMR (400 MHz, $CDCl_3$): δ = 7.53 (m, 4H, aryl-H), 7.35 (m, 4H, aryl-H), 7.27 (m, 2H, aryl-H), 7.16 (br, 2H, thiophene-H), 3.76 (s, 6H, NCH_3), 2.35-2.10 (br, 6H, thiophene-CH_3) ppm.

^{13}C{^{1}H}-NMR (101 MHz, $CDCl_3$): δ = 143.0 (br, $SCCH_3$), 142.8 (SCC_{aryl}), 133.3 (C_q), 133.2 ($CClN_2$), 129.2 (aryl-C), 128.7 (imidazole-C_q), 128.2 (aryl-C), 125.7 (aryl-C), 123.5 (thiophene-CH), 122.1 (thiophene-C_q), 34.2 (NCH_3), 14.1 (CH_3) ppm.

^{11}B-NMR (128 MHz, $CDCl_3$): δ = –1.2 ppm.

^{11}B{^{1}H}-NMR (128 MHz, $CDCl_3$): δ = –1.2 ppm.

^{19}F-NMR (376 MHz, $CDCl_3$): δ = –154.5 ppm.

^{19}F{^{1}H}-NMR (376 MHz, $CDCl_3$): δ = –154.5 ppm.

HRMS (ESI): m/z calculated for $[C_{27}H_{24}ClN_2S_2]^+$ $(M)^+$ 475.10639, found 475.10609.

IR (neat): $\tilde{\nu}$ = 692 (vs, ν(C–S)), 711 (w), 756 (vs, ν(C–S)), 857 (w), 878 (m), 907 (w), 948 (m), 1053 (vs), 1095 (s), 1172 (w), 1244 (w), 1325 (w), 1413 (m), 1442 (s), 1468 (m), 1508 (m), 1531 (s), 1598 (m), 2917 (w), 3022 (w) cm^{-1}.

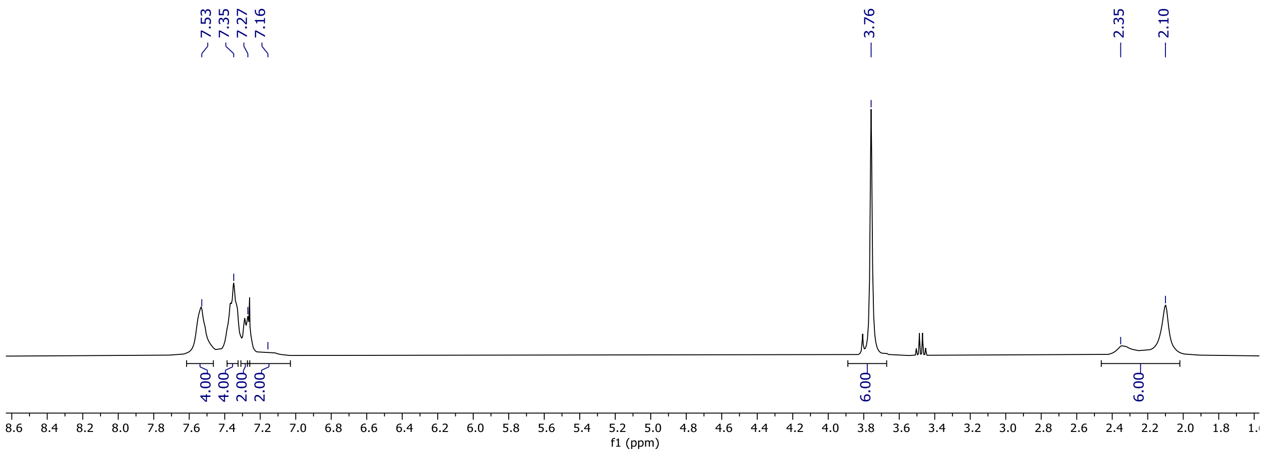

Figure S32: ^{1}H NMR spectrum (in $CDCl_3$, 300 K, 400 MHz) of **6**.

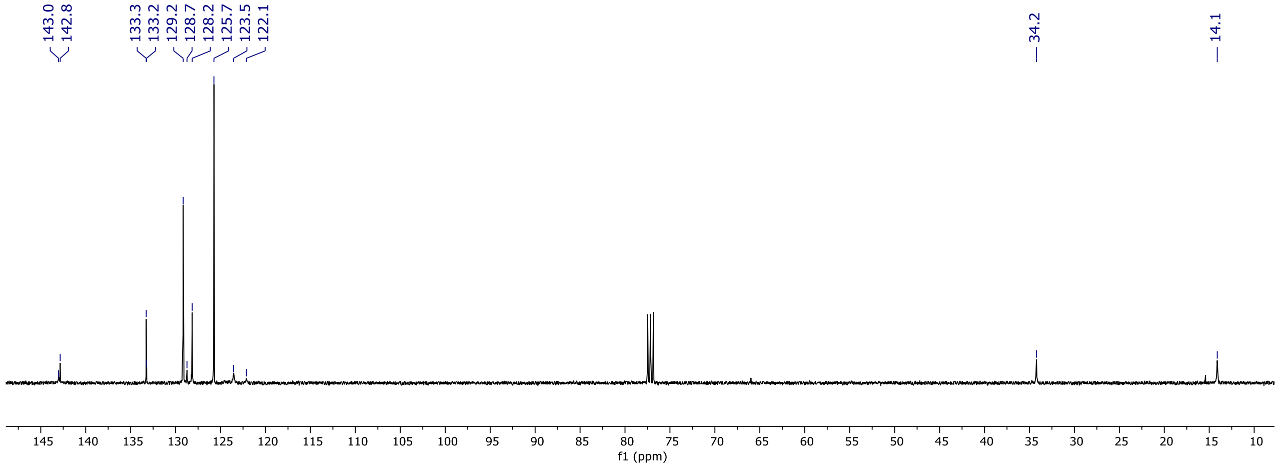

Figure S13: $^{13}C\{^{1}H\}$ NMR spectrum (in $CDCl_3$, 300 K, 101 MHz) of **6**.

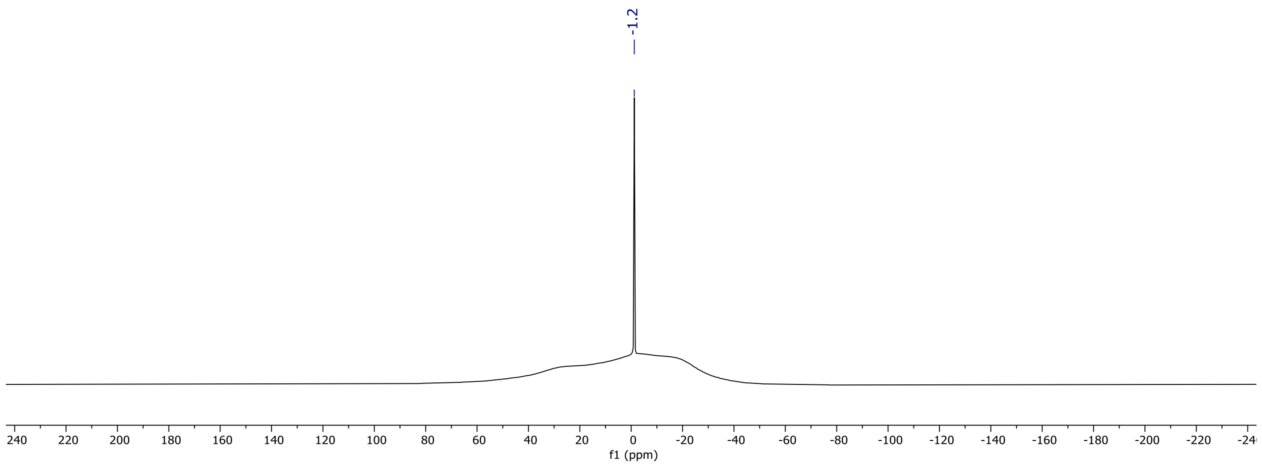

Figure S14: ^{11}B NMR spectrum (in $CDCl_3$, 300 K, 128 MHz) of **6**.

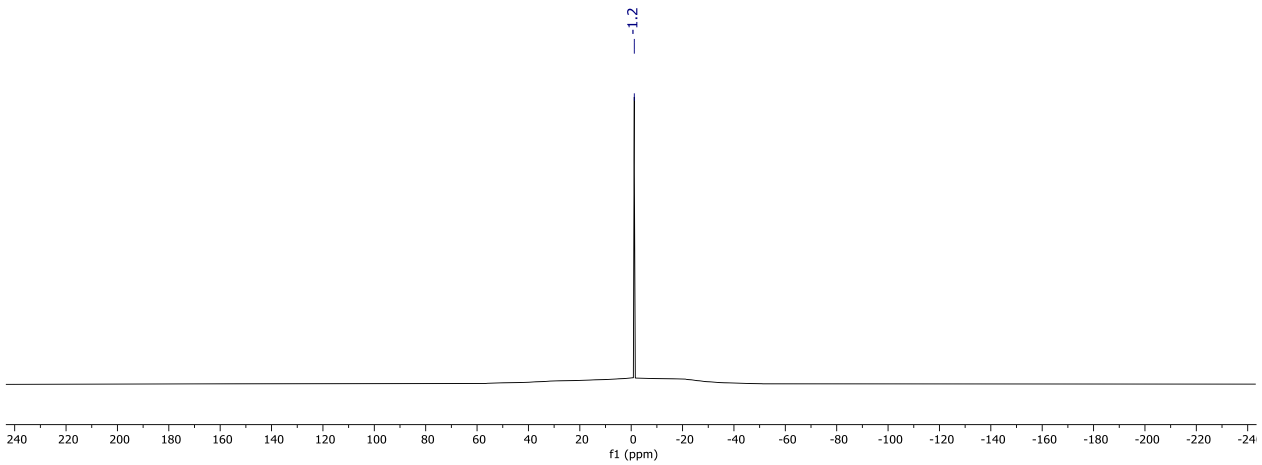

Figure S15: $^{11}B\{H\}$ NMR spectrum (in $CDCl_3$, 300 K, 128 MHz) of **6**.

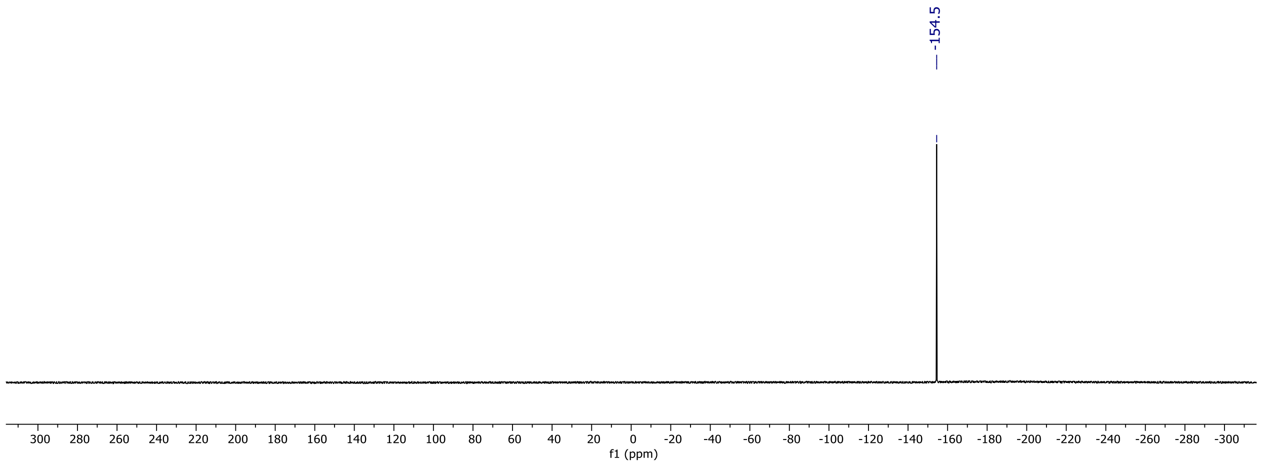

Figure S16: ^{19}F NMR spectrum (in $CDCl_3$, 300 K, 376 MHz) of **6**.

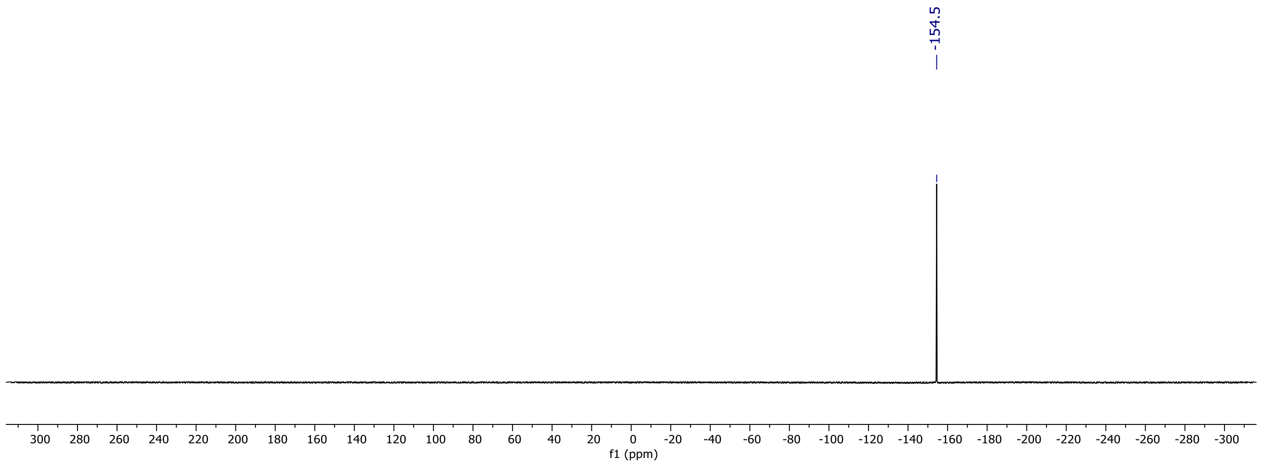

Figure S17: $^{19}F\{H\}$ NMR spectrum (in $CDCl_3$, 300 K, 376 MHz) of **6**.

Preparation of 7HBF4

A Schlenk flask was charged with **6** (300 mg, 533 µmol, 1.00 eq.), anhydrous KF (186 mg, 3.20 mmol, 6.00 eq.) and $MeNH_3Cl$ (72.0 mg, 1.07 mmol, 2.00 eq.). MeCN (5 mL) was added and the resulting suspension was stirred at room temperature for 2 d. $CHCl_3$ (5 mL) was added and the suspension was stirred for another 5 min at room temperature. The solid components were filtered off and the solution was transferred into a separation funnel. A diluted aq. solution of $NaBF_4$ (293 mg, 2.67 mmol, 5.00 eq.) was added to the MeCN/$CHCl_3$ suspension followed by vigorous shaking. The organic phase was separated and the aqueous phase was extracted once with a small amount of $CHCl_3$. The combined organic fractions were dried (anhydrous $MgSO_4$), filtered and the solvent was removed under reduced pressure to afford a colorless solid. After drying at 80 °C in vacuo overnight, **7**HBF_4 was obtained in 90% yield as a white solid (268 mg, 481 µmol).

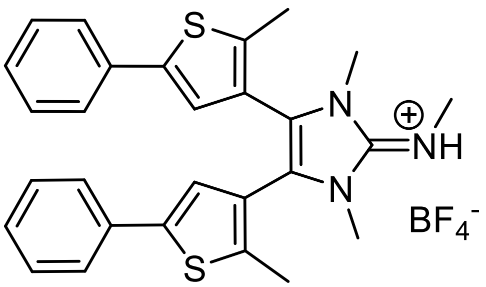

^{1}H-NMR (400 MHz, DMSO-d_6): δ = 7.59 (m, 4H, aryl-H), 7.57 (m, 2H, thiophene-H), 7.42 (m, 4H, aryl-H), 7.32 (m, 2H, aryl-H), 3.81 (br, 1H, NH), 3.49 (s, 6H, NCH_3), 3.20 (d, $^3J_{HH}$ = 5.0 Hz 3H, NCH_3), 2.07-1.99 (br, 6H, thiophene-CH_3) ppm.

^{13}C{^{1}H}-NMR (101 MHz, DMSO-d_6): δ = 147.7 (NCN_2), 140.7 (SCC_{aryl}), 140.1 ($SCCH_3$), 132.9 (C_q), 129.2 (aryl-CH), 127.9 (aryl-CH), 125.1 (aryl-CH), 124.5 (br, thiophene-CH), 124.2 (thiophene-C_q), 121.8 (imidazole-C_q), 32.3 (2 x NCH_3), 31.4 (NCH_3), 13.5 (CH_3) ppm.

^{11}B-NMR (128 MHz, DMSO-d_6): δ = –1.3 ppm.

^{11}B{^{1}H}-NMR (128 MHz, DMSO-d_6): δ = –1.3 ppm.

^{19}F-NMR (376 MHz, DMSO-d_6): δ = –148.4 ppm.

^{19}F{^{1}H}-NMR (376 MHz, DMSO-d_6): δ = –148.4 ppm.

HRMS (ESI): m/z calculated for $[C_{28}H_{28}N_3S_2]^+$ $(M)^+$ 470.17192, found 470.17179.

IR (neat): $\tilde{\nu}$ = 689 (vs, ν(C–S)), 724 (w), 755 (vs, ν(C–S)), 852 (m), 948 (w), 1032 (vs), 1053 (vs), 1157 (w), 1184 (w), 1203 (w), 1332 (w), 1396 (m), 1407 (m), 1445 (m), 1468 (m), 1507 (m), 1567 (m), 1617 (s), 2856 (w), 2921 (m), 3064 (w), 3363 (m) cm^{-1}.

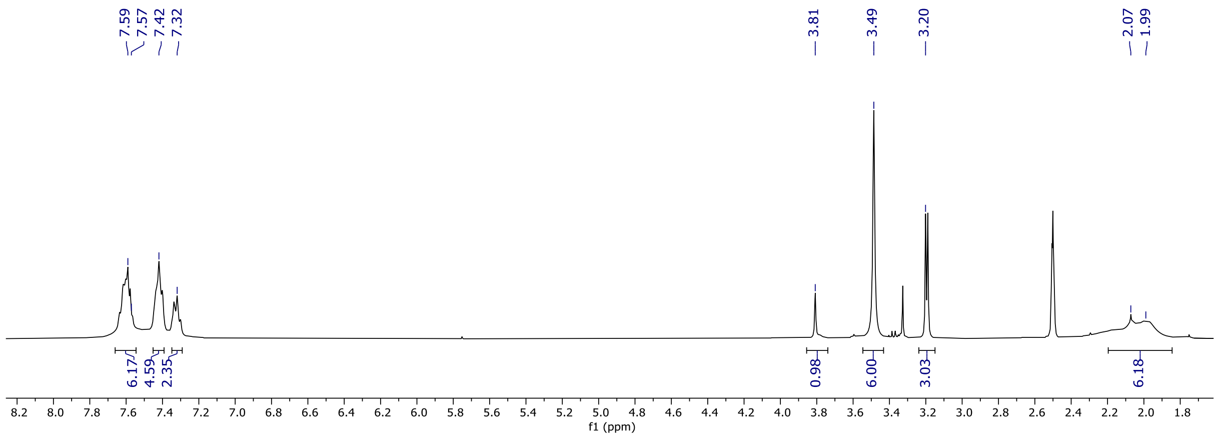

Figure S18: ^{1}H NMR spectrum (in DMSO-d_6, 300 K, 400 MHz) of **7**HBF_4.

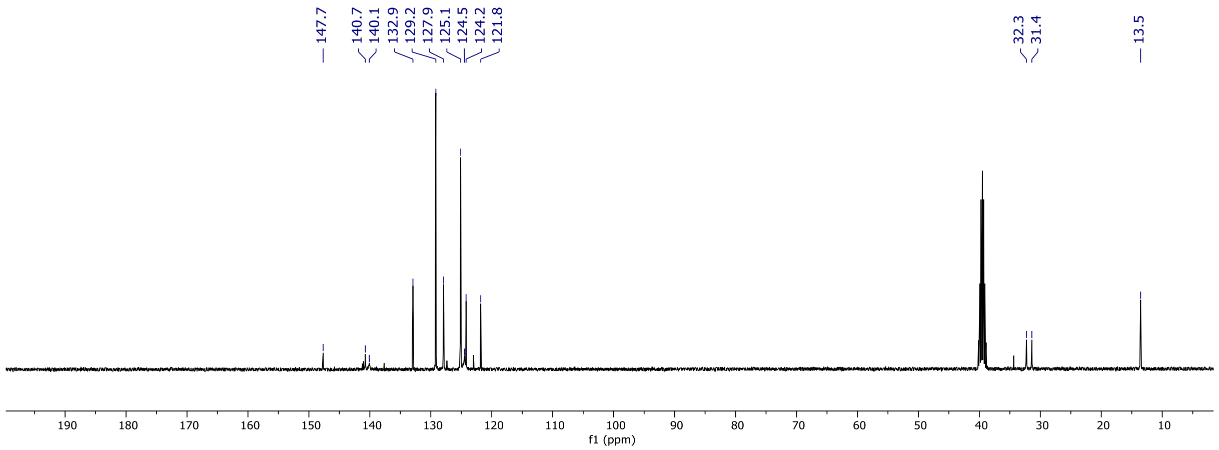

Figure S19: $^{13}C\{^{1}H\}$ NMR spectrum (in DMSO-d_6, 300 K, 101 MHz) of **7**HBF_4.

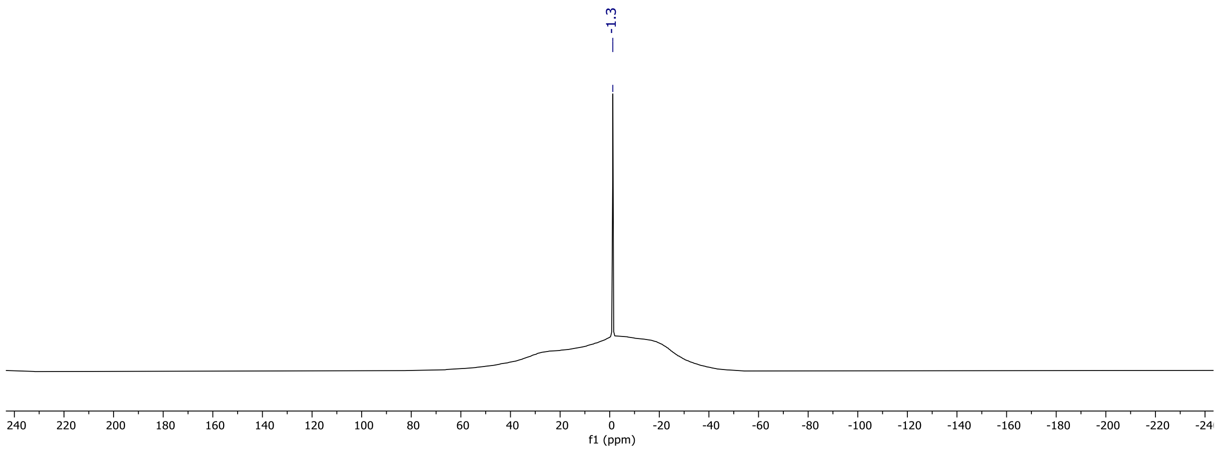

Figure S20: ^{11}B NMR spectrum (in DMSO-d_6, 300 K, 128 MHz) of **7**HBF_4.

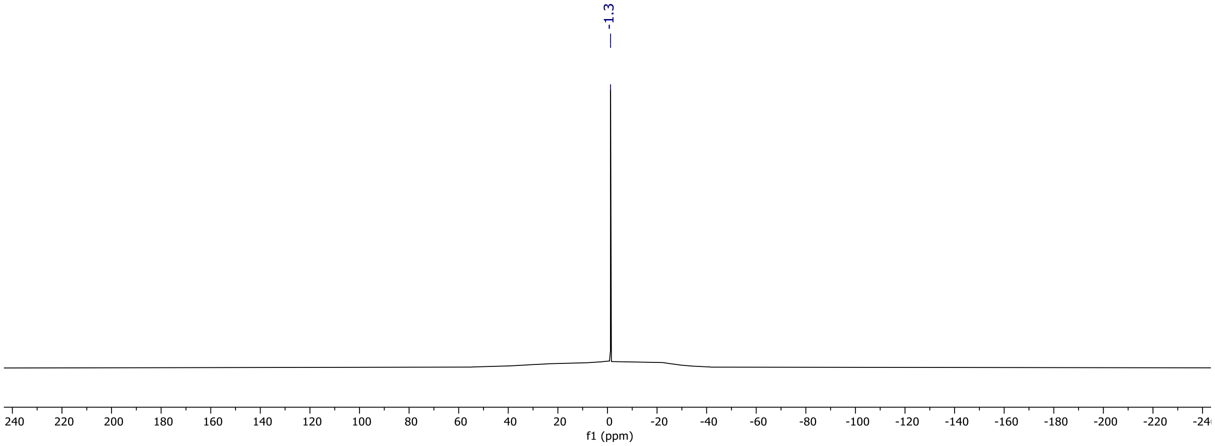

Figure S21: $^{11}B\{H\}$ NMR spectrum (in DMSO-d_6, 300 K, 128 MHz) of **7**HBF_4.

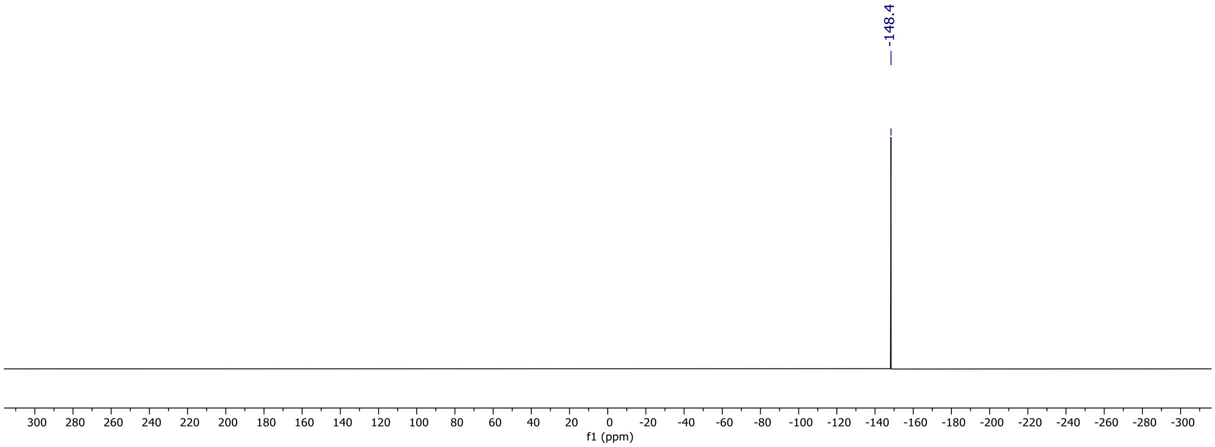

Figure S22: ^{19}F NMR spectrum (in DMSO-d_6, 300 K, 376 MHz) of **7**HBF_4.

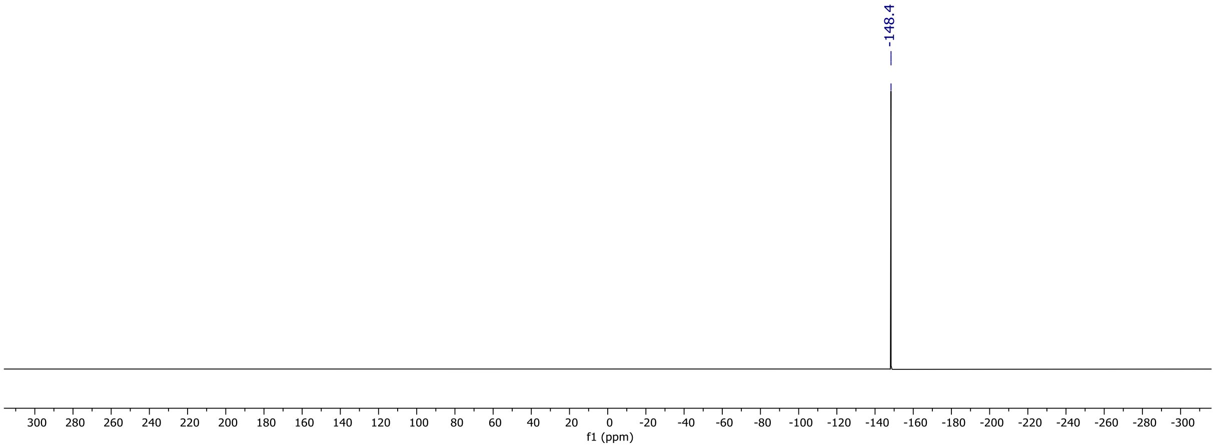

Figure S23: $^{19}F\{H\}$ NMR spectrum (in DMSO-d_6, 300 K, 376 MHz) of **7**HBF_4.

Preparation of 8HBF4

A Schlenk flask was charged with **6** (160 mg, 284 µmol, 1.00 eq.*)*, anhydrous KF (98.8 mg, 1.70 mmol, 6.00 eq.) and tert-butylamine (62.3 mg, 43.6 µL, 852 µmol, 3.00 eq.). MeCN (5 mL) was added and the resulting suspension was stirred at room temperature for 3 d. $CHCl_3$ (5 mL) was added and the suspension was stirred for another 5 min at room temperature. The solid components were filtered off and the solution was transferred into a separation funnel. A diluted aq. solution of $NaBF_4$ (156 mg, 1.42 mmol, 5.00 eq.) was added to the MeCN/$CHCl_3$ suspension followed by vigorous shaking. The organic phase was separated and the aqueous phase was extracted once with a small amount of $CHCl_3$. The combined organic fractions were dried (anhydrous $MgSO_4$), filtered and the solvent was removed under reduced pressure to afford a colorless solid. After drying at 80 °C in vacuo overnight, **8**HBF_4 was obtained in 82% yield as a white solid (143 mg, 239 µmol).

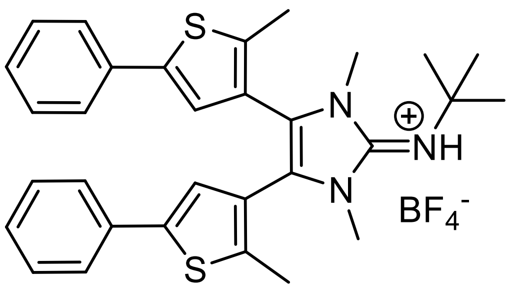

^{1}H-NMR (400 MHz, $CDCl_3$): δ = 7.53 (d, $^3J_{HH}$ = 7.6 Hz, 4H, aryl-H), 7.37 (m, $^3J_{HH}$ = 7.5 Hz, 4H, aryl-H), 7.29 (t, $^3J_{HH}$ = 7.4 Hz, 2H, aryl-CH), 7.17 (br, 2H, thiophene-H), 4.80 (s, 1H, NH), 3.65 (s, 6H, NCH_3), 2.13 (br, 6H, thiophene-CH_3), 1.42 (s, 9H, $C(CH_3)_3$) ppm.

^{13}C{^{1}H}-NMR (101 MHz, $CDCl_3$): δ = 145.3 (NCN_2), 142.8 (SCC_{aryl}), 141.4 ($SCCH_3$), 133.3 (C_q), 129.2 (aryl-CH), 128.2 (aryl-CH), 125.7 (aryl-CH), 125.6 (thiophene-CH), 125.4 (thiophene-C_q), 123.4 (imidazole-C_q), 57.5 ($NC(CH_3)_3$), 33.9 (NCH_3), 31.1 (3 x CH_3), 14.1 (2 x CH_3) ppm.

^{11}B-NMR (128 MHz, $CDCl_3$): δ = -1.0 ppm.

^{11}B{^{1}H}-NMR (128 MHz, $CDCl_3$): δ = -1.0 ppm.

^{19}F-NMR (376 MHz, $CDCl_3$): δ = -152.7 ppm.

^{19}F{^{1}H}-NMR (376 MHz, $CDCl_3$): δ = -152.7 ppm.

HRMS (ESI): m/z calculated for $[C_{31}H_{34}N_3S_2]^+$ $(M)^+$ 512.21887, found 512.21836.

IR (neat): $\tilde{\nu}$ = 688 (vs, ν(C–S)), 755 (vs, ν(C–S)), 855 (m), 946 (w), 1016 (vs), 1061 (vs), 1186 (m), 1224 (m), 1370 (w), 1388 (m), 1435 (m), 1444 (m), 1467 (m), 1506 (w), 1536 (m), 1558 (m), 1598 (m), 1652 (w), 2924 (w), 2973 (m), 3064 (w), 3319 (s) cm^{-1}.

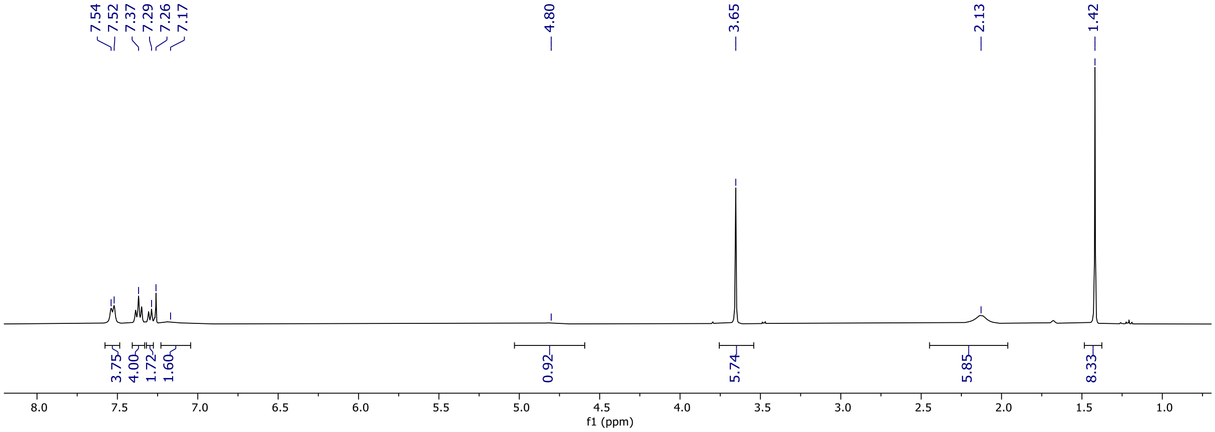

Figure S24: ^{1}H NMR spectrum (in $CDCl_3$, 300 K, 400 MHz) of **8**HBF_4.

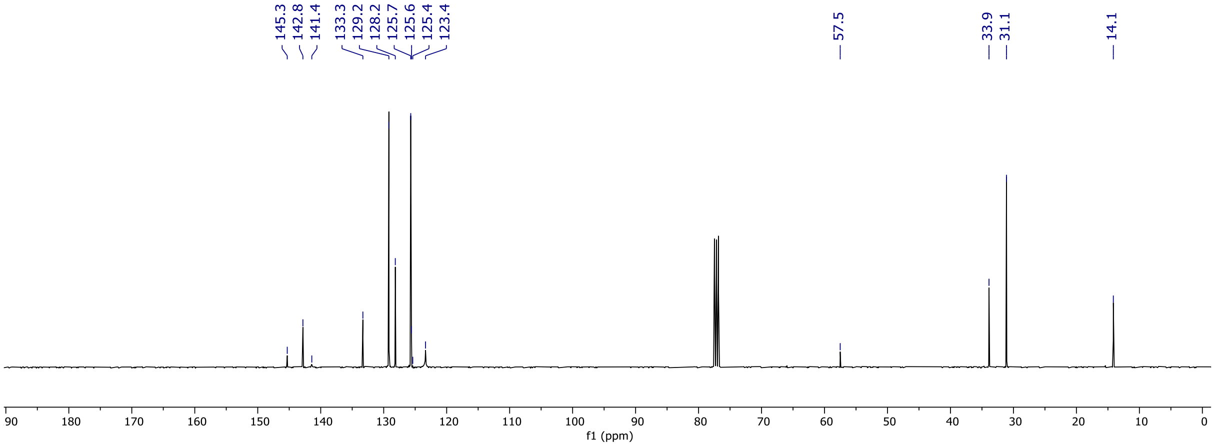

Figure S25: $^{13}C\{^{1}H\}$ NMR spectrum (in $CDCl_3$, 300 K, 101 MHz) of **8**HBF_4.

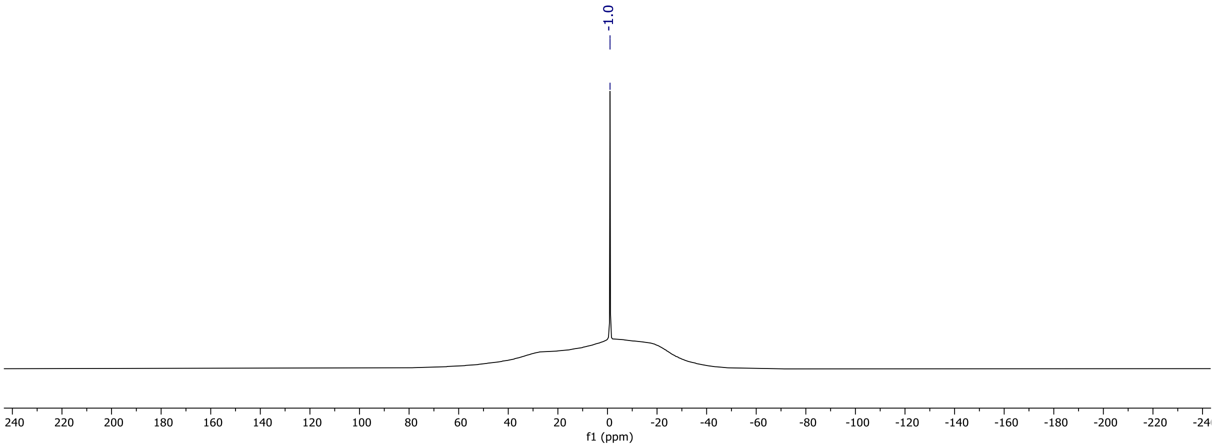

Figure S26: ^{11}B NMR spectrum (in $CDCl_3$, 300 K, 128 MHz) of **8**HBF_4.

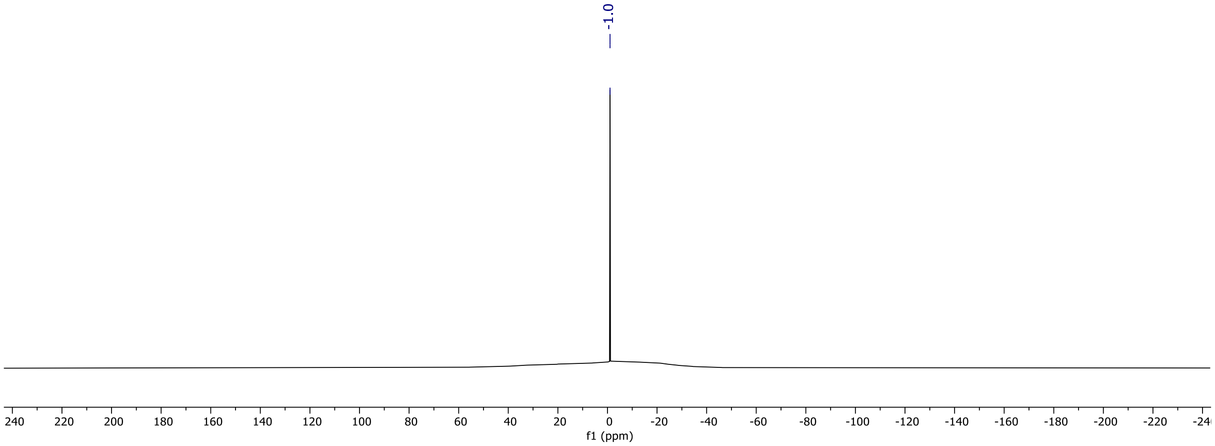

Figure S27: $^{11}B\{H\}$ NMR spectrum (in $CDCl_3$, 300 K, 128 MHz) of **8**HBF_4.

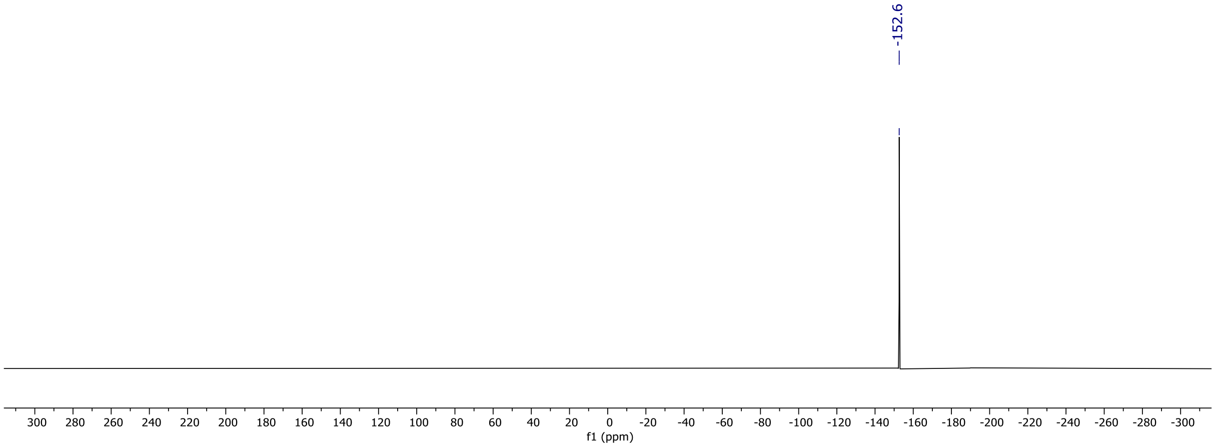

Figure S28: ^{19}F NMR spectrum (in $CDCl_3$, 300 K, 376 MHz) of **8**HBF_4.

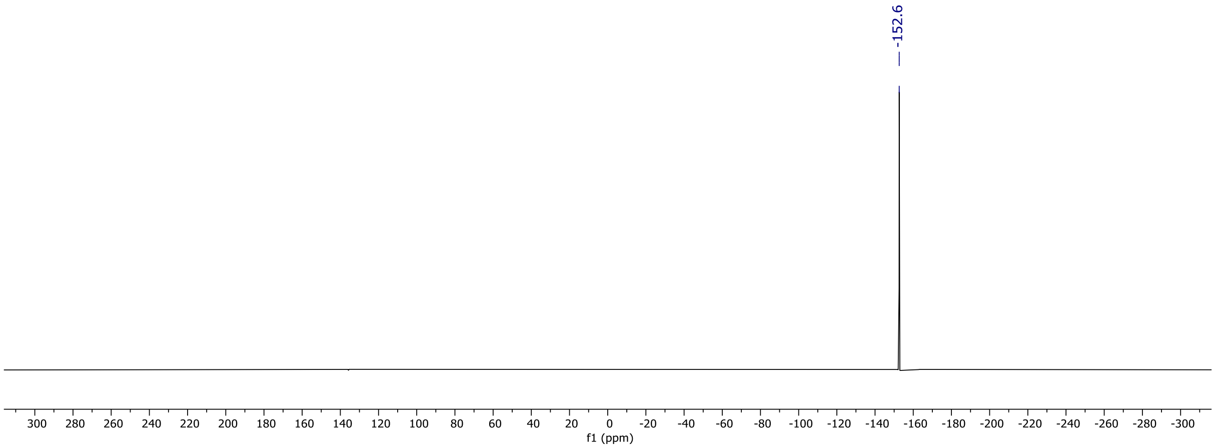

Figure S29: $^{19}F\{H\}$ NMR spectrum (in $CDCl_3$, 300 K, 376 MHz) of **8**HBF_4.

Preparation of 7

A Schlenk flask was charged with a solution of the **7**HBF4 (75.0 mg, 135 µmol, 1.00 eq.) in THF (5.00 mL). A diluted THF solution of KOtBu (14.8 mg, 132 µmol, 0.98 eq.) was added dropwise and the resulting suspension was stirred at room temperature for 2h. The volatiles were evaporated and **7** was extracted twice with n-hexane. The solvent was removed under reduced pressure to afford **7** in 94% yield as a pale yellow oil (59.2 mg, 126 µmol).

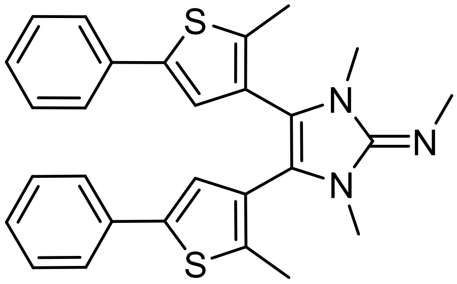

^{1}H-NMR (500 MHz, MeCN-d_3): δ = 7.56 (d, $^3J_{HH}$ = 7.2, 4H, aryl-H), 7.37 (t, $^3J_{HH}$ = 7.7 Hz, 4H, aryl-H), 7.28 (t, $^3J_{HH}$ = 7.4, 2H, aryl-H), 7.17 (s, 2H, thiophene-H), 3.28 (s, 3H, NCH_3), 3.13 (s, 6H, $2xNCH_3$), 2.09 (s, 6H, $2xCH_3$) ppm.

^{13}C{^{1}H}-NMR (126 MHz, MeCN-d_3): δ = 151.7 (NCN_2), 141.3 (SCC_{aryl}), 138.8 ($SCCH_3$), 134.9 (C_q), 130.0 (aryl-CH), 129.1 (thiophene-C_q), 128.5 (aryl-CH), 126.2 (aryl-CH), 126.1 (thiophene-CH), 120.2 (imidazole-C_q), 35.3 (NCH_3), 32.2 ($2xNCH_3$), 14.3 ($2xCH_3$) ppm.

HRMS (ESI): m/z calculated for $[C_{28}H_{28}N_3S_2]^+$ $(M+H)^+$ 470.17192, found 470.17171.

IR (neat): $\tilde{\nu}$ = 689 (vs, ν(C–S)), 755 (vs, ν(C–S)) 844 (m), 905 (w), 947 (m), 983 (s), 983 (s), 994 (s), 1031 (w), 1057 (m), 1124 (s), 1183 (s), 1236 (vs), 1305 (s), 1364 (s), 1401 (s), 1434 (m), 1468 (m), 1504 (m), 1600 (m), 1633 (vs), 1656 (vs, ν(C=N)), 2854 (w), 2913 (w) cm^{-1}.

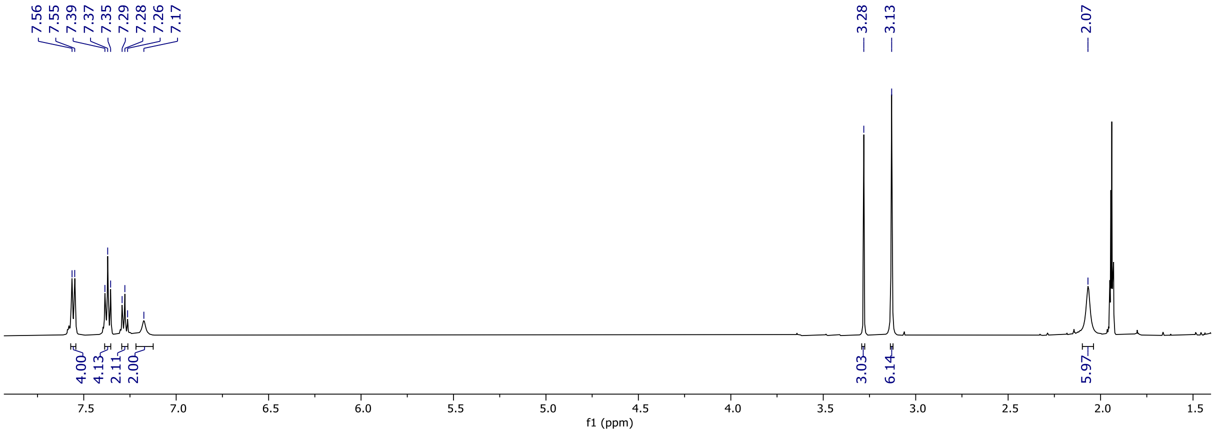

Figure S30: ^{1}H NMR spectrum (in MeCN-d_3, 300 K, 500 MHz) of **7**.

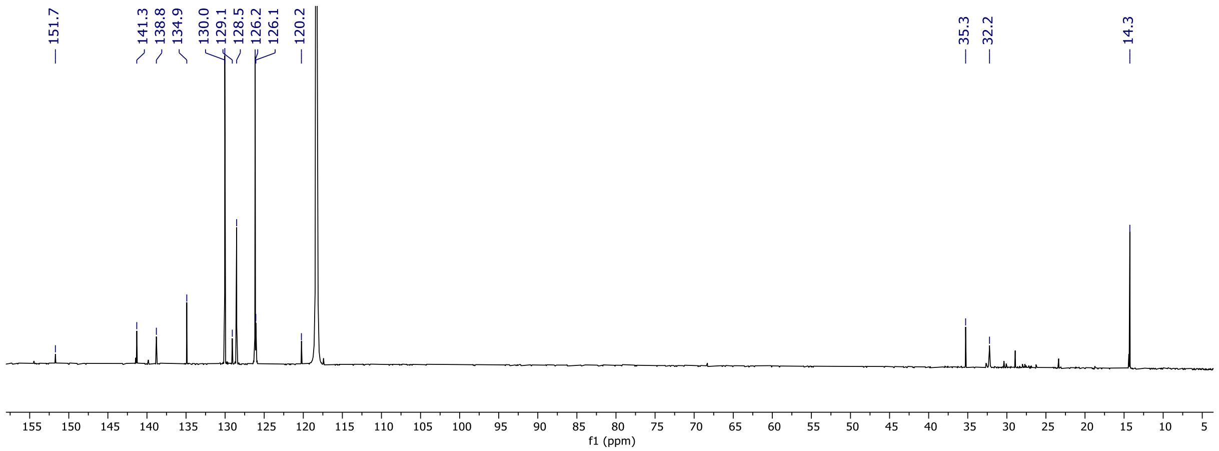

Figure S31: $^{13}C\{^1H\}$ NMR spectrum (in MeCN-d_3, 300 K, 126 MHz) of **7**.

Preparation of 8

A Schlenk flask was charged with a solution of the **8**HBF4 (130 mg, 217 µmol, 1.00 eq.) in THF (5.00 mL). A diluted THF solution of KOtBu (23.9 mg, 213 µmol, 0.98 eq.) was added dropwise and the resulting suspension was stirred at room temperature for 2h. The volatiles were evaporated and **8** was extracted twice with n-hexane. The solvent was removed under reduced pressure to afford **8** in 94% yield as a white solid (103 mg, 201 µmol).

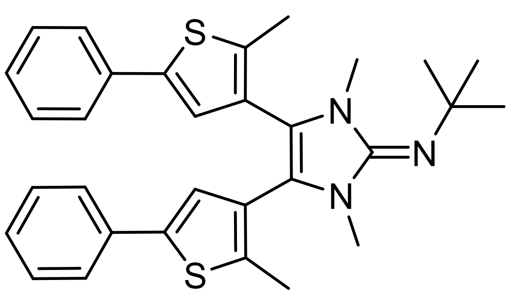

^{1}H-NMR (400 MHz, MeCN-d$_3$): δ = 7.55 (d, $^3J_{HH}$ = 7.2, 4H, aryl-H), 7.36 (t, $^3J_{HH}$ = 7.6 Hz, 4H, aryl-H), 7.27 (t, $^3J_{HH}$ = 7.4, 2H, aryl-H), 7.14 (s, 2H, Thiophen-H), 3.05 (s, 6H, NCH_3), 2.05 (s, 6H, CH_3), 1.39 (s, 9H, 3xCH_3) ppm.

^{13}C{^{1}H}-NMR (101 MHz, MeCN-d$_3$): δ = 147.8 (NCN_2), 141.3 (SCC_{Aryl}), 138.1 ($SCCH_3$), 134.9 (C_q), 130.0 (Aryl-CH), 129.7 (Thiophen-C_q), 128.5 (Aryl-CH), 126.2 (Aryl-CH), 126.0 (Thiophen-CH), 120.6 (Imidazol-C_q), 51.6 ($NC(CH_3)$), 34.1 (3xCH_3), 33.9 (2xNCH_3), 14.2 (2xCH_3) ppm.

HRMS (ESI): m/z calculated for $[C_{31}H_{34}N_3S_2]^+$ $(M+H)^+$ 512.21887, found 512.21920.

IR (neat): $\tilde{\nu}$ = 689 (vs, ν(C–S)), 755 (vs, ν(C–S)) 840 (w), 888 (w), 905 (w), 947 (w), 1011 (m), 1031 (m), 1057 (w), 1116 (w), 1155 (w), 1197 (m), 1277 (m), 1361 (m), 1383 (s), 1437 (s), 1504 (m), 1550 (m), 1600 (m), 1685 (vs, ν(C=N)), 2914 (w), 2962 (w), 3060 (w) cm^{-1}.

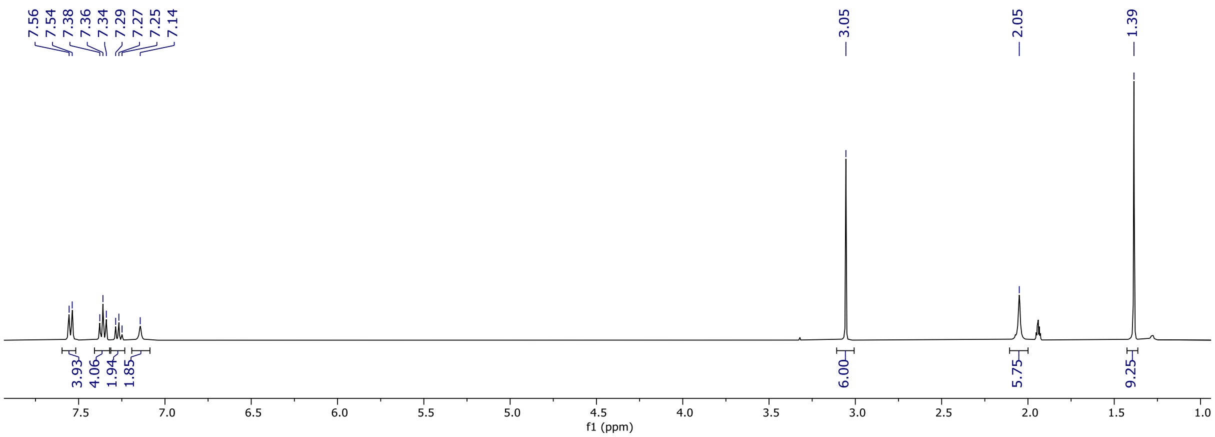

Figure S32: ^{1}H NMR spectrum (in MeCN-d_3, 300 K, 400 MHz) of **8**.

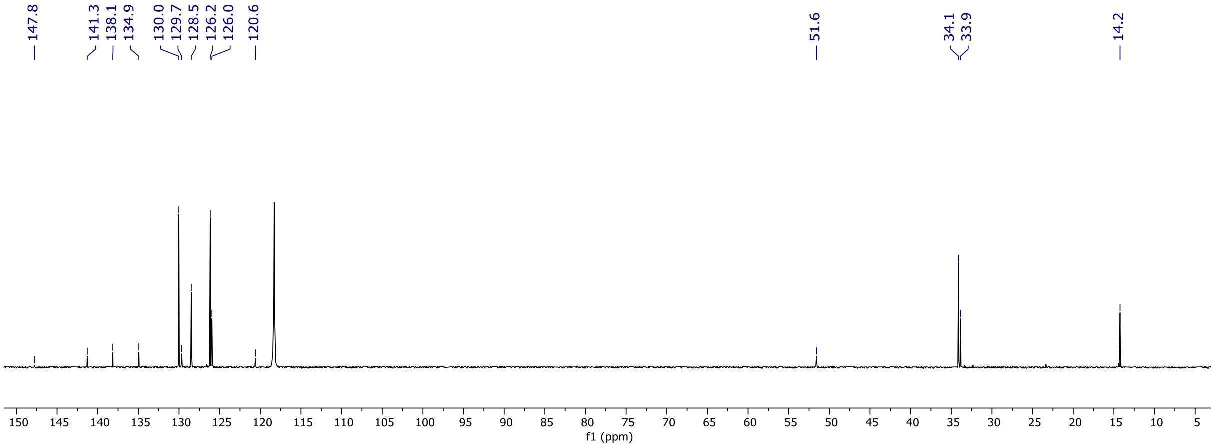

Figure S33: ^{13}C{^{1}H} NMR spectrum (in MeCN-d_3, 300 K, 101 MHz) of **8**.

Preparation of 8CO$_2$

8 (8.00 mg, 15.6 µmol) was dissolved in MeCN-d_3 and the NMR tube was pressurized with two bar $^{13}CO_2$ pressure.

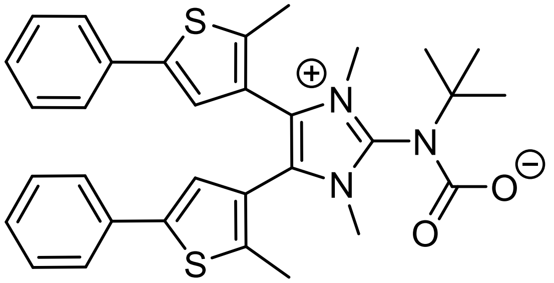

^{1}H-NMR (500 MHz, MeCN-d_3): δ = 7.60 (d, $^{3}J_{HH}$ = 7.8 Hz, 4H, aryl-H), 7.39 (t, $^{3}J_{HH}$ = 7.8 Hz, 4H, aryl-H), 7.31 (d, $^{3}J_{HH}$ = 7.4 Hz, 2H, aryl-CH), 7.29 (s, 2H, thiophene-H), 3.58 (s, 6H, 2xNCH$_3$), 2.14 (br, 6H, 2xCH$_3$), 1.48 (s, 9H, C(CH$_3$)$_3$) ppm.

^{13}C{^{1}H}-NMR (126 MHz, MeCN-d_3): δ = 154.6 (NCO$_2$), 142.8 (NCN$_2$), 142.5 (SCC$_{aryl}$), 134.3 (SCCH$_3$), 130.1 (aryl-CH), 130.1 (C$_q$), 129.0 (aryl-CH), 128.6 (thiophene-C$_q$), 126.4 (aryl-CH), 126.2 (thiophene-CH), 125.1 (imidazole-C$_q$), 57.5 (NC(CH$_3$)), 30.0 (3xCH$_3$), 28.9 (2xNCH$_3$), 14.4 (2xCH$_3$) ppm.

IR (neat): $\tilde{\nu}$ = 689 (vs, ν(C–S)), 753 (vs, ν(C–S)) 840 (m), 905 (w), 969 (w), 1007 (s), 1031 (m), 1052 (w), 1072 (w), 1115 (m), 1153 (w), 1196 (s), 1260 (w), 1351 (s), 1375 (m), 1415 (m), 1468 (w), 1505 (m), 1601 (s), 1626 (vs), 1650 (s, ν(C–O)), 1688 (w), 2863 (w), 2914 (w), 2961 (m) cm^{-1}.

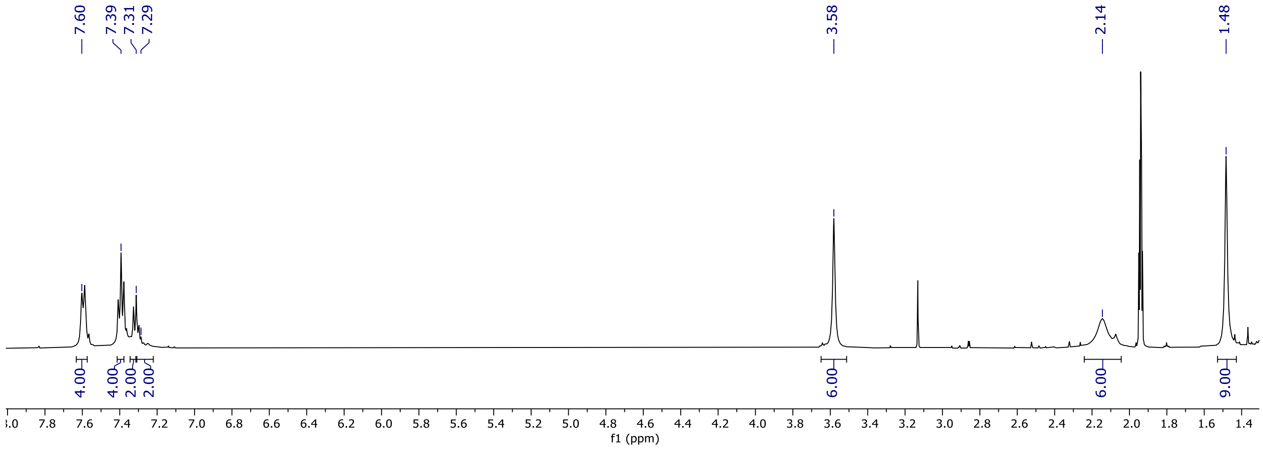

Figure S34: ^{1}H NMR spectrum (in MeCN-d_3, 300 K, 500 MHz) of **$8CO_2$**.

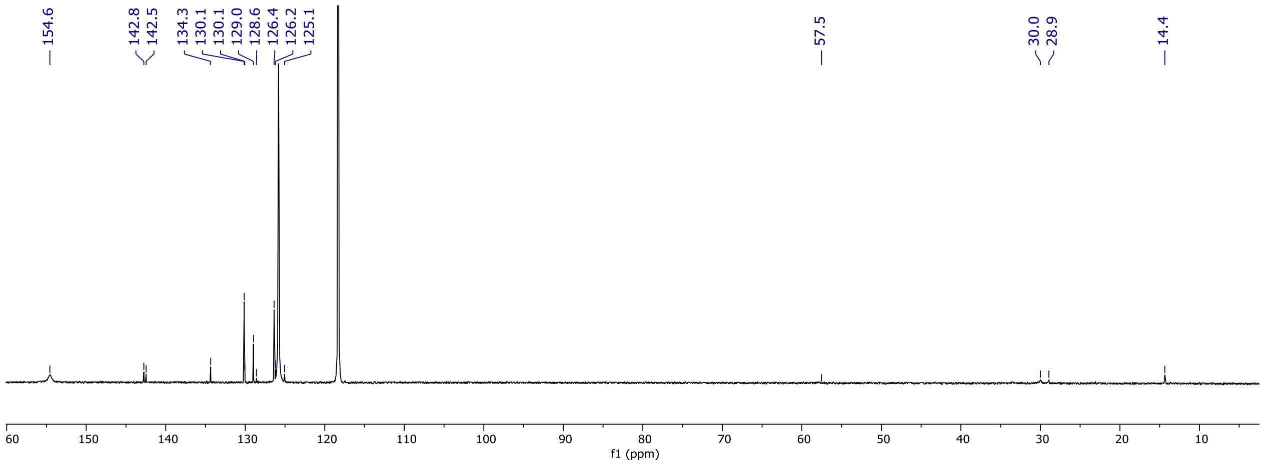

Figure S35: ^{13}C{^{1}H} NMR spectrum (in MeCN-d_3, 300 K, 126 MHz) of **$8CO_2$**.

Variable-temperature NMR Study towards the formation of **7**CO_2

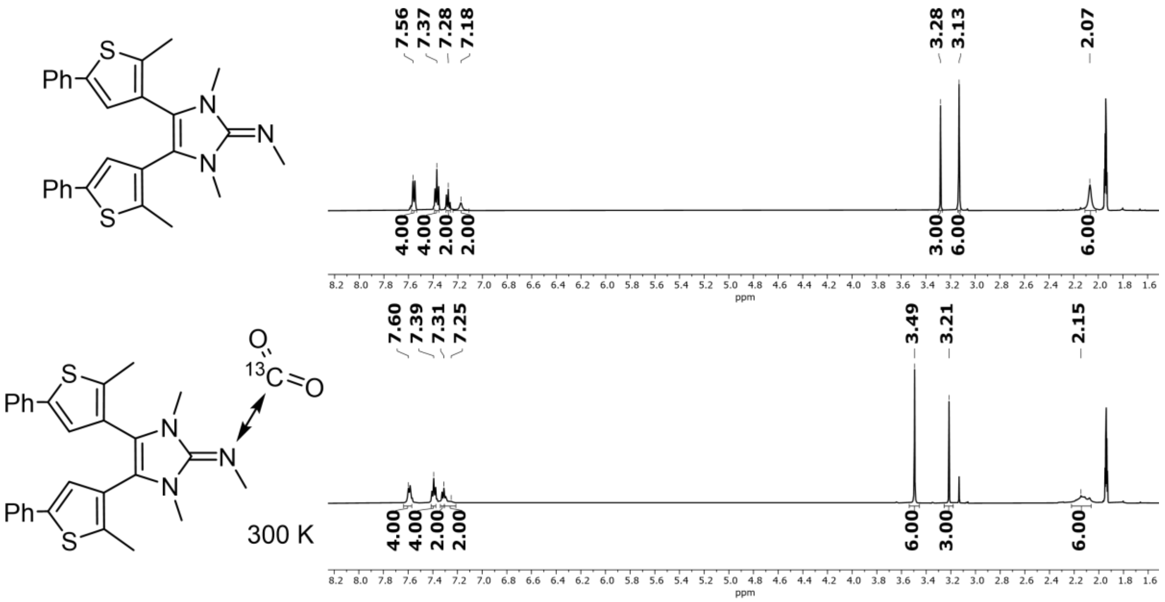

Figure S36: ^{1}H NMR spectra (MeCN-d_3, 300 K) of a solution of **7c** (top) and of the same solution after the addition of with 1 bar $^{13}CO_2$ (bottom).

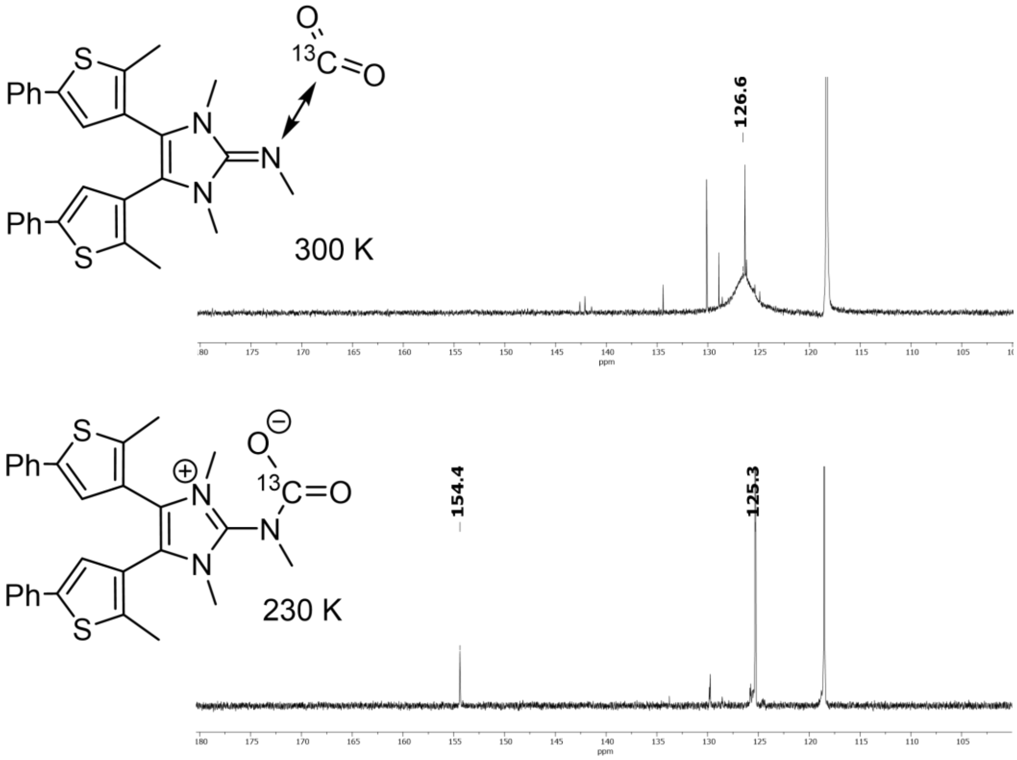

Figure S37: ^{13}C NMR spectra of **7o** in MeCN-d_3 with 2 bar ^{13}C-enriched $^{13}CO_2$ at 300K (top) and 230K (bottom), respectively.

Photoinduced cyclization of 8

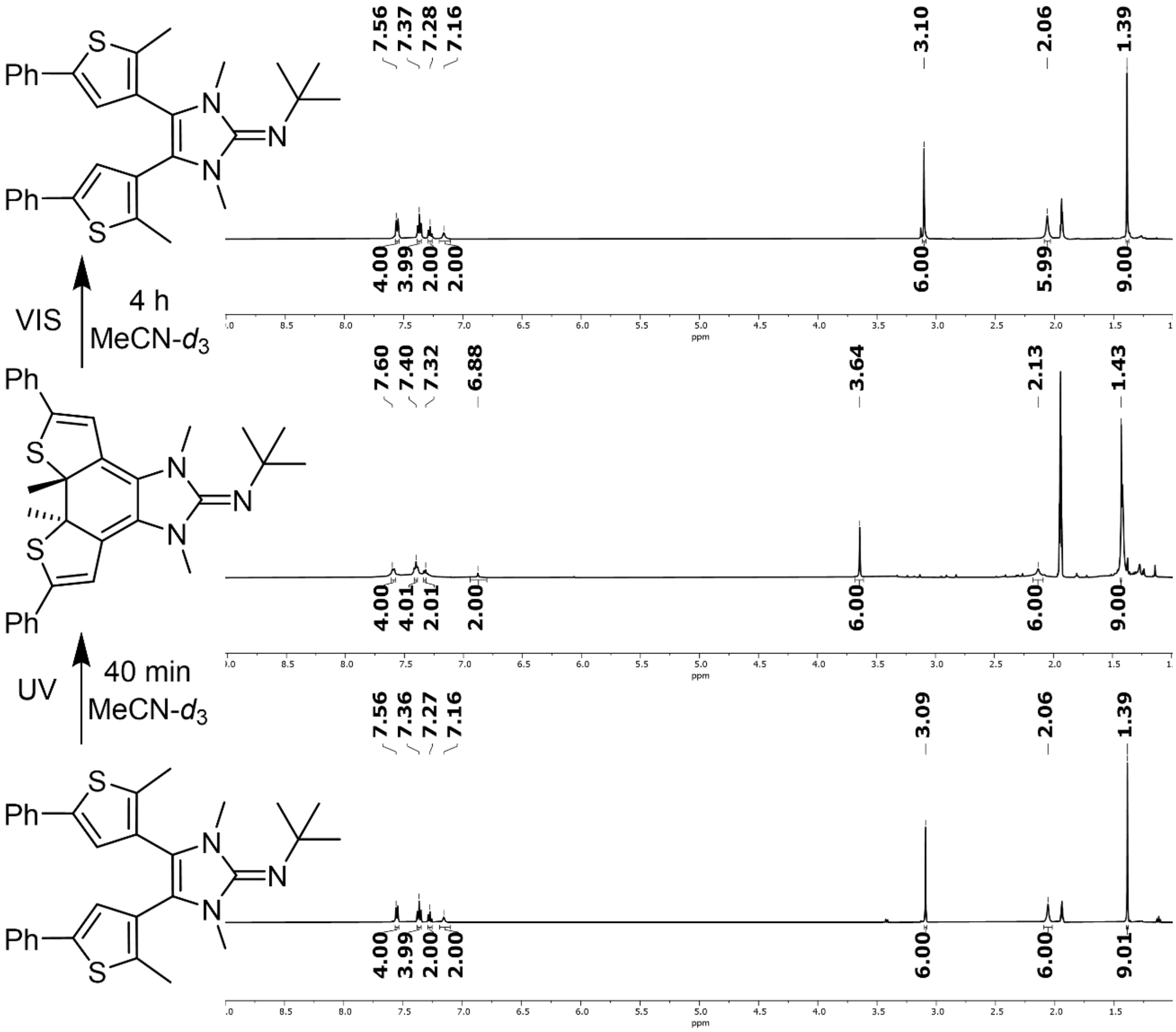

Figure S38: ^{1}H NMR spectra of **8o** in MeCN-d_3 (bottom), of **8c** after irradiation of the solution with UV light (313 nm) for 40 min (middle) and of **8o** after further irradiation of the solution with visible light (500 nm) for 4 h (top).

UV/vis Spectroscopic Data

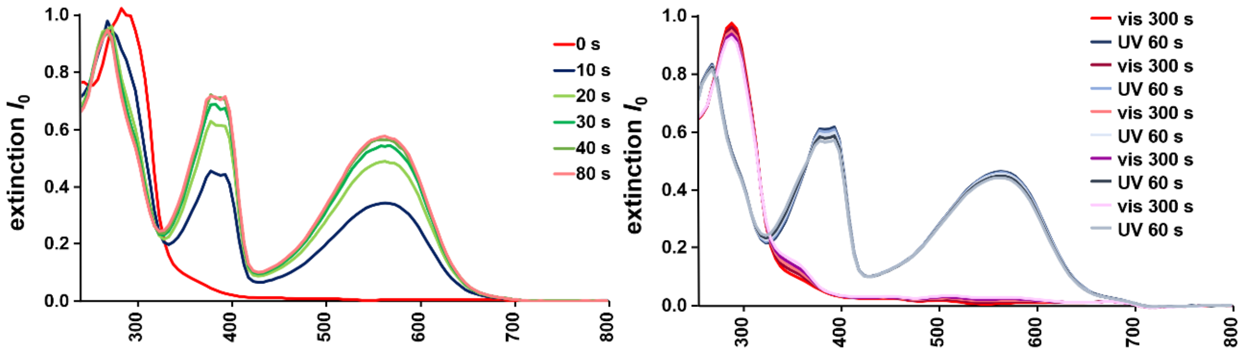

Figure S39: Left: UV/vis spectral changes of **7o** upon UV irradiation (λ_{Irr} = 313 nm). The spectra were recorded after 10, 20, 30, 40 and 80 s. Right: UV/VIS spectral changes of **7o** upon successive UV (λ_{irr} = 254 nm, t = 60 s) and visible-light irradiation (λ_{irr} = 520 nm, t = 300 s). Measured in MeCN ([**7o**] = 7.1 x 10^{-4} M).

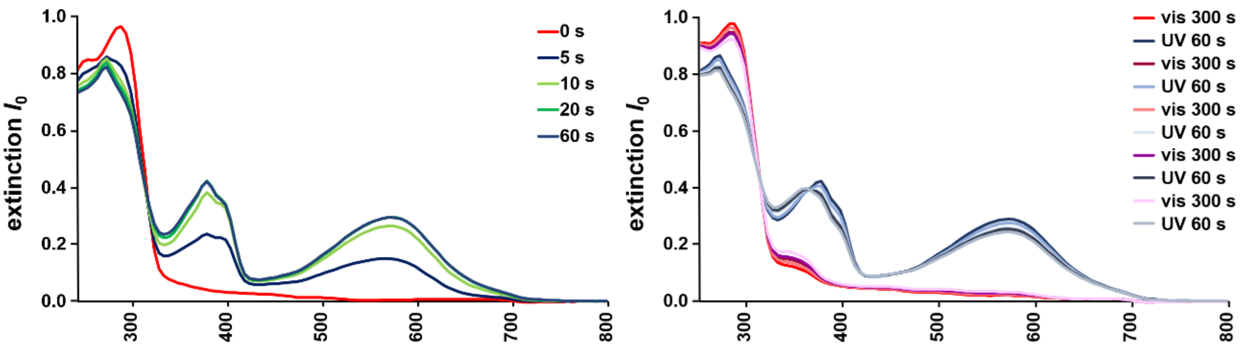

Figure S40: Left: UV/vis spectral changes of **8o** upon UV irradiation (λ_{Irr} = 313 nm). The spectra were recorded after 5, 10, 20, 30 and 60 s. Right: UV/VIS spectral changes of **7o** upon successive UV (λ_{irr} = 254 nm, t = 60 s) and visible-light irradiation (λ_{irr} = 520 nm, t = 300 s). Measured in MeCN ([**8o**] = 3.2 x 10^{-4} M).

Photoswitchable CO2 activation

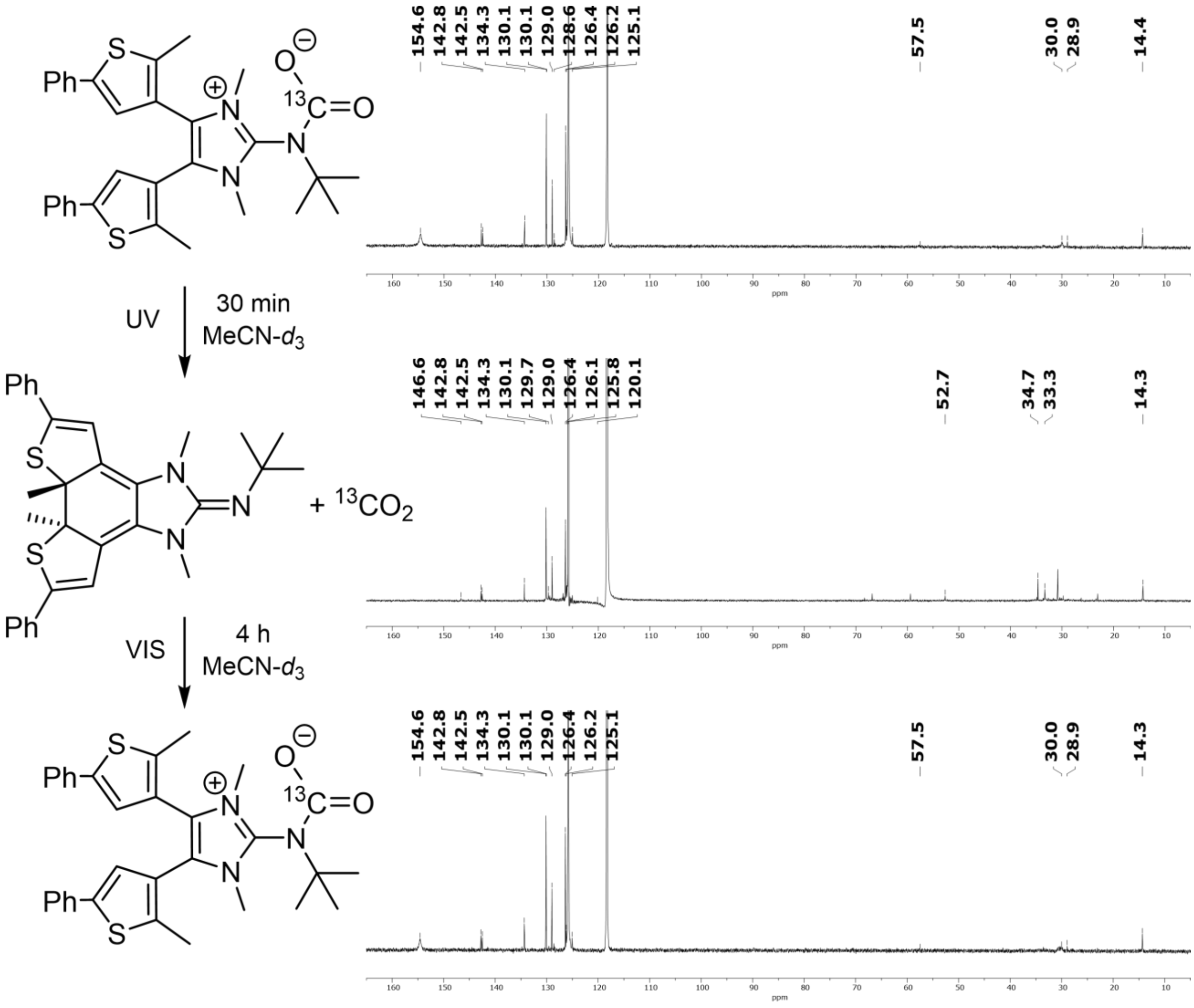

Figure S41: $^{13}C\{^{1}H\}$ NMR spectra (MeCN-*d*$_3$, 300 K, 126 MHz) of **8CO$_2$** (top), of the same solution after irradiation with UV light (313 nm, 30 min) (middle) and of the same solution after a further irradiation with VIS light (540 nm, 4 h) (bottom).

X-ray Diffraction Studies

General: Single-crystal X-ray diffraction data were collected on a Bruker AXS detector using Mo-K_α radiation (λ = 0.71073 Å). Crystals were selected under oil, mounted on nylon loops and then immediately placed in a cold stream of N_2 on a diffractometer. Using Olex2,[5] the structures were solved with the Superflip[6] structure solution program using Charge Flipping and refined with the ShelXL[7] refinement package using Least Squares minimisation.

Crystallographic data have been deposited with the Cambridge Crystallographic Data Centre as supplementary publication no. CCDC-2108444 (**7**HBF_4), CCDC-2108445 (**8**HBF_4), CCDC-2108446 (**8o**) and CCDC-2108447 (**8c**). These data can be obtained free of charge via www.ccdc.cam.uk/data_request/cif (or from the CCDC, 12 Union Road, Cambridge CB2 1EZ, UK; fax: (+44) 1223-336-033; or deposit@ccdc.cam.ac.uk).

Single-crystal X-ray structure analysis of 7HBF$_4$:

Single crystals were obtained by cooling down a hot and saturated $CHCl_3$ solution of **7**HBF$_4$. A Single-crystal X-ray structure analysis revealed that **7**HBF$_4$ crystallizes in the monoclinic space group $P2_1/c$. The asymmetric unit contains one molecule of **7**HBF$_4$.

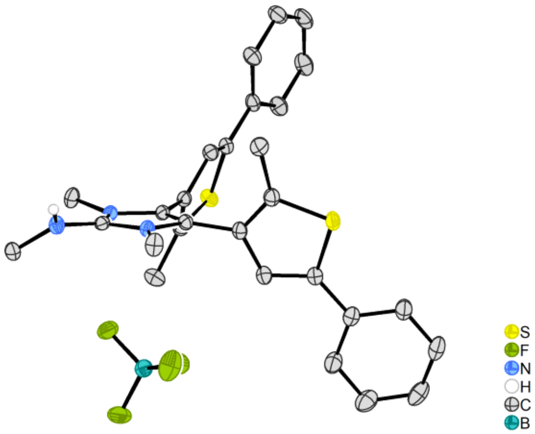

Figure S42: Molecular view of **7**HBF$_4$ in the solid state with thermal ellipsoid plot at the 50% levels of probability. Hydrogen atoms except N–H-Proton are omitted for clarity.

Table S1. Crystal data and structure refinement for **7**HBF$_4$.

CCDC number	2108444	ρ_{calc}g/cm^3	1.384
Empirical formula	$C_{28}H_{28}BF_4N_3S_2$	μ/mm^{-1}	0.250
Formula weight	557.46	F(000)	1160.0
Temperature/K	100	Crystal size/mm^3	0.301 × 0.248 × 0.19
Crystal system	monoclinic	Radiation	MoKα (λ = 0.71073)
Space group	$P2_1/c$	2Θ range for data collection/°	4.49 to 56.712
a/Å	17.9520(4)	Index ranges	$-23 \le h \le 23$, $-10 \le k \le 10$, $-24 \le l \le 24$
b/Å	8.2126(2)	Reflections collected	40921
c/Å	18.5866(4)	Independent reflections	6676 [R_{int} = 0.0589, R_{sigma} = 0.0389]
α/°	90	Data/restraints/parameters	6676/0/352
β/°	102.5250(10)	Goodness-of-fit on F^2	1.030
γ/°	90	Final R indexes [I>=2σ (I)]	R_1 = 0.0411, wR_2 = 0.0958
Volume/Å^3	2675.06(11)	Final R indexes [all data]	R_1 = 0.0544, wR_2 = 0.1041
Z	4	Largest diff. peak/hole / e Å^{-3}	0.63/-0.47

Single-crystal X-ray structure analysis of 8HBF$_4$

Single crystals were obtained by the diffusion of Et_2O into a $CHCl_3$ solution of **8**HBF$_4$. A Single-crystal X-ray structure analysis revealed that **8**HBF$_4$ crystallizes in the monoclinic space group $P2_1$. The asymmetric unit contains one molecule of **8**HBF$_4$.

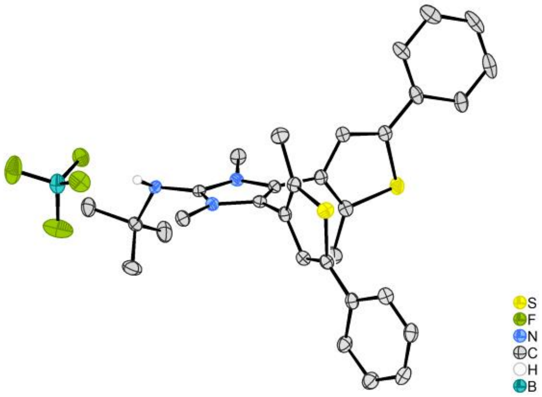

Figure S43: Molecular view of **8**HBF$_4$ in the solid state with thermal ellipsoid plot at the 50% levels of probability. Hydrogen atoms except N–H-Proton are omitted for clarity.

Table S2. Crystal data and structure refinement for **8**HBF$_4$**.**

CCDC number	2108445	μ/mm^{-1}	0.232
Empirical formula	$C_{31}H_{34}BF_4N_3S_2$	F(000)	628.0
Formula weight	599.54	Crystal size/mm^3	0.367 × 0.1 × 0.063
Temperature/K	100	Radiation	MoKα (λ = 0.71073)
Crystal system	monoclinic	2Θ range for data collection/°	4.278 to 52.788
Space group	$P2_1$	Index ranges	$-12 \leq h \leq 12$, $-11 \leq k \leq 11$, $-20 \leq l \leq 20$
a/Å	9.9733(3)	Reflections collected	20375
b/Å	9.2535(3)	Independent reflections	6040 [R_{int} = 0.0448, R_{sigma} = 0.0437]
c/Å	16.7764(5)	Data/restraints/parameters	6040/1/381
α/°	90	Goodness-of-fit on F^2	1.039
β/°	107.283(2)	Final R indexes [I>=2σ (I)]	R_1 = 0.0332, wR_2 = 0.0770
γ/°	90	Final R indexes [all data]	R_1 = 0.0364, wR_2 = 0.0792
Volume/Å^3	1478.35(8)	Largest diff. peak/hole / e Å^{-3}	0.34/-0.20
Z	2	Flack parameter	0.02(3)
ρ_{calc}g/cm^3	1.347		

Single-crystal X-ray structure analysis of 8o

Single crystals were obtained by cooling down a saturated *n*-pentane solution of **8o** to –34 °C. A Single-crystal X-ray structure analysis revealed that **8o** crystallizes in the triclinic space group *P*-1. The asymmetric unit contains one molecule of **8o** and a disordered *n*-pentane molecule.

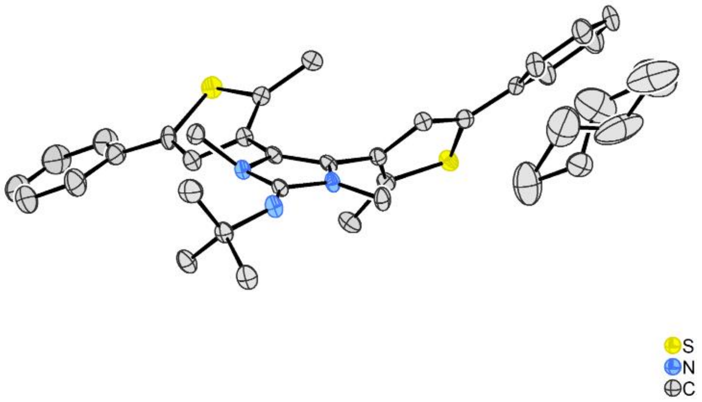

Figure S44: Molecular view of **8o** in the solid state with thermal ellipsoid plot at the 50% levels of probability. Hydrogen atoms are omitted for clarity.

Table S3. Crystal data and structure refinement for **8o.**

CCDC number	2108446	ρ_{calc}g/cm^3	1.195
Empirical formula	$C_{36}H_{45}N_3S_2$	μ/mm^{-1}	0.193
Formula weight	583.87	*F*(000)	628.0
Temperature/K	100	Crystal size/mm^3	0.428 × 0.172 × 0.029
Crystal system	triclinic	Radiation	MoKα (λ = 0.71073)
Space group	*P*-1	2Θ range for data collection/°	3.32 to 52.822
a/Å	9.6183(5)	Index ranges	$-12 \le h \le 12$, $-17 \le k \le 17$, $-18 \le l \le 18$
b/Å	13.7730(7)	Reflections collected	19984
c/Å	14.5419(7)	Independent reflections	6593 [R_{int} = 0.0542, R_{sigma} = 0.0557]
α/°	63.994(3)	Data/restraints/parameters	6593/12/398
β/°	71.070(3)	Goodness-of-fit on F^2	1.048
γ/°	88.295(3)	Final *R* indexes [I>=2σ (I)]	R_1 = 0.0637, wR_2 = 0.1482
Volume/Å^3	1623.32(15)	Final *R* indexes [all data]	R_1 = 0.0812, wR_2 = 0.1566
Z	2	Largest diff. peak/hole / e Å^{-3}	0.88/-0.56

Single-crystal X-ray structure analysis of 8c

Single crystals were obtained by storing a MeCN solution of **8c** at room temperature for two months. A Single-crystal X-ray structure analysis revealed that **8c** crystallizes in the orthorhombic space group *P*na2_1. The asymmetric unit contains one molecule of **8c**.

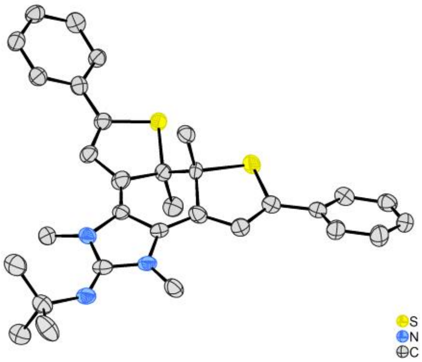

Figure S45: Molecular view of **8c** in the solid state with thermal ellipsoid plot at the 50% levels of probability. Hydrogen atoms are omitted for clarity.

Table S4. Crystal data and structure refinement for **8c.**

CCDC number	2108447	μ/mm^{-1}	0.227
Empirical formula	$C_{31}H_{33}N_3S_2$	*F*(000)	1088.0
Formula weight	511.72	Crystal size/mm^3	0.423 × 0.389 × 0.049
Temperature/K	100	Radiation	MoKα (λ = 0.71073)
Crystal system	orthorhombic	2Θ range for data collection/°	2.986 to 51.928
Space group	*P*na2_1	Index ranges	-33 ≤ h ≤ 33, -9 ≤ k ≤ 10, -13 ≤ l ≤ 14
a/Å	27.286(4)	Reflections collected	14580
b/Å	8.3958(13)	Independent reflections	5020 [R_{int} = 0.0992, R_{sigma} = 0.0881]
c/Å	11.5623(16)	Data/restraints/parameters	5020/1/371
α/°	90	Goodness-of-fit on F^2	1.102
β/°	90	Final *R* indexes [*I*>=2σ (*I*)]	R_1 = 0.0638, wR_2 = 0.1533
γ/°	90	Final *R* indexes [all data]	R_1 = 0.0816, wR_2 = 0.1635
Volume/Å^3	2648.8(6)	Largest diff. peak/hole / e Å^{-3}	0.72/-0.35
Z	4	Flack parameter	-0.20(7)
ρ_{calc}g/cm^3	1.283		

DFT Calculations

All structures were optimized without geometry constraints using the TPSS meta GGA functional[8] and an atom-pairwise dispersion correction (D3)[9]. A flexible triple zeta basis set (def2-TZVP)[10] was used in all calculations. The minimum character of all optimized stationary points was proven by the absence of imaginary vibrational frequencies. For the calculation of the free energy contributions from vibrational frequencies using the rigid-rotor-harmonic-oscillator approach (G^{RRHO}(298K)), a rotor approximation was applied to vibrational modes with wave numbers below 100 cm^{-1}.[11] Electronic energies were recalculated with the hybrid functional PW6B95(-D3)[12] using the structures optimized with TPSS-D3. Solvation free energies were obtained with the COSMO-RS model[13] for implicit solvation with CH_3CN as solvent at 298.15 K. All quantum chemical calculations were performed with the TURBOMOLE 7.5 program.[14]

Gas phase basicities (GB) were calculated from the values in Table S5 (with $G_{298}(H^+)$=-6.22 kcal/mol):

$$GB = -\{ [E_{PW6B95\text{-}D3}(\mathbf{X\text{-}H}^+)-E_{PW6B95\text{-}D3}(\mathbf{X})] + [G_{298}^{RRHO}(\mathbf{X\text{-}H}^+)-G_{298}^{RRHO}(\mathbf{X})-G_{298}(H^+)]\}$$

Proton affinities (PA) were calculated from the values in Table S5 (with $H_{298}(H^+)$=1.48 kcal/mol):

$$PA = -\{[E_{PW6B95\text{-}D3}(\mathbf{X\text{-}H}^+)-E_{PW6B95\text{-}D3}(\mathbf{X})] + [H_{298}^{RRHO}(\mathbf{X\text{-}H}^+)-H_{298}^{RRHO}(\mathbf{X})-H_{298}(H^+)]\}$$

Predicted p*K*a values for the corresponding Brønsted acids of compounds **7o**, **7c**, **8o**, **8c, NHC-o,** and **NHC-c** were obtained based on the calculated free energy of the proton exchange equilibrium with **NHI^{Me}** in acetonitrile:

$$\mathbf{X\text{-}H}^+ + \mathbf{NHI^{Me}} \rightarrow \mathbf{X} + \mathbf{NHI^{Me}\text{-}H}^+ \qquad \Delta G_{prot\text{-}trans}$$

$$\Delta G_{prot\text{-}trans} = \Delta E_{prot\text{-}trans}(PW6B95\text{-}D3) + \Delta G^{rrho}_{prot\text{-}trans} + \Delta G(COSMO\text{-}RS)_{prot\text{-}trans}$$

From this we obtain the relative pKa value of **X-H** vs. **NHI^{Me}-H**:

$$pKa(rel) = \Delta G_{prot\text{-}trans} / (RT \ln(10)),$$

and the pKa value of **X-H**$^+$ using the experimental value of 27.2 for **NHI^{Me}** as reference:[15]

$$pKa(\mathbf{X\text{-}H}^+) = 27.2 + pKa(rel) = 27.2 + \Delta G_{prot\text{-}trans} / (RT \ln(10)).$$

Calculated GB, PA and pKa values are compiled in Table S6.

Table S5 Calculated DFT Energies (E(TPSS-D3) and E(PW6B95-D3) in E_h), thermostatistical corrections for translation, rotation and vibration (H_{298}^{RRHO}, G_{298}^{RRHO} in kcal/mol) in the gas phase (*vac*) for all molecules in the DFT study. COSMO-RS solvation free energies in kcal/mol using acetonitrile as solvent at 298 K. The def2-TZVP basis set was used in all calculations.

	**E(TPSS-D3)** [E_h]	**G_{298}^{RRHO}** [kcal/mol]	**H_{298}^{RRHO}** [kcal/mol]	**E(PW6B95-D3)** [E_h]	**ΔG(COSMO-RS)** [kcal/mol]
**7o**	-2044.732248	260.515	318.531	-2046.676000	-19.127
**7o-H**	-2045.156896	269.562	327.650	-2047.100542	-47.599
**7c**	-2044.715084	262.844	318.590	-2046.652594	-19.750
**7c-H**	-2045.123797	271.372	327.563	-2047.060347	-49.908
**8o**	-2162.743686	309.752	372.820	-2164.811054	-18.946
**8o-H**	-2163.176655	319.199	381.851	-2165.244885	-45.115
**8c**	-2162.726206	312.259	372.899	-2164.787430	-19.909
**8c-H**	-2163.143060	321.152	381.773	-2165.202916	-46.485
**NHC-o**	-1949.984848	235.306	289.359	-1951.833386	-19.805
**NHC-o-H**	-1950.431619	244.176	297.937	-1952.277240	-47.681
**NHC-c**	-1949.955738	237.026	289.021	-1951.796445	-19.280
**NHC-c-H**	-1950.396494	245.860	297.728	-1952.232386	-48.143
**NHI^{Me}**	-635.762138	182.871	222.112	-636.425147	-4.817
**NHI^{Me}-H**	-636.190499	191.435	231.391	-636.852960	-35.896

Table S6 Calculated gas phase basicity (GB), proton affinity (PA), free energy of proton transfer and predicted p*K*a values (in CH_3CN), using the energies from Table S5.

Compound	GB [kcal/mol]	PA [kcal/mol]	$\Delta G_{prot\text{-}trans}$ [kcal/mol]	p*K*a(**XH^+**) (CH_3CN)
**7o**	251.1	258.8	-5.1	23.4
**7c**	241.1	248.4	-13.5	17.3
**8o**	256.6	264.7	-2.0	25.7
**8c**	245.6	253.3	-13.9	17.0
**NHC-o**	263.8	271.4	+6.6	32.0
**NHC-c**	258.9	266.3	+2.6	29.1
**NHI^{Me}**	253.7	260.7	0.0	*27.2* (exp.)[15]

Figure S46 Optimized molecular structures (TPSS-D3/def2-TZVP) of the imines, NHCs and protonated bases investigated in this DFT study.

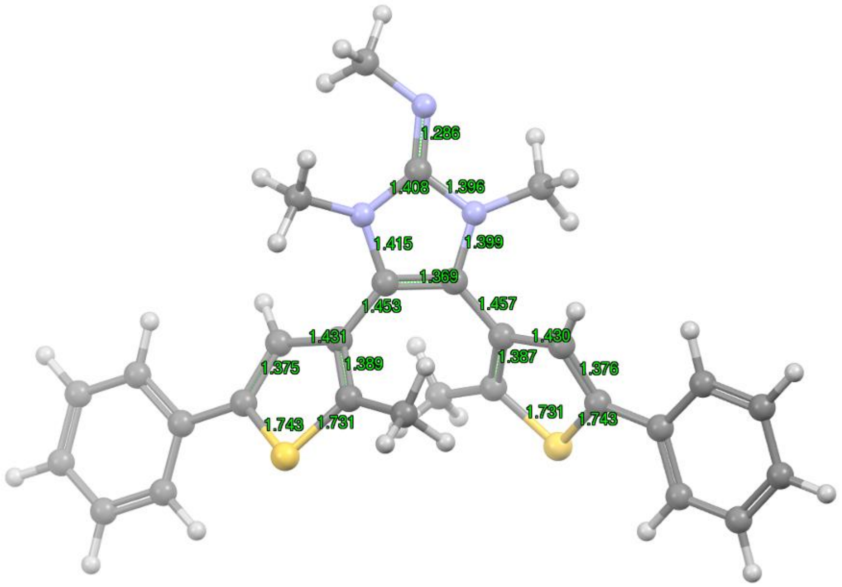

7o

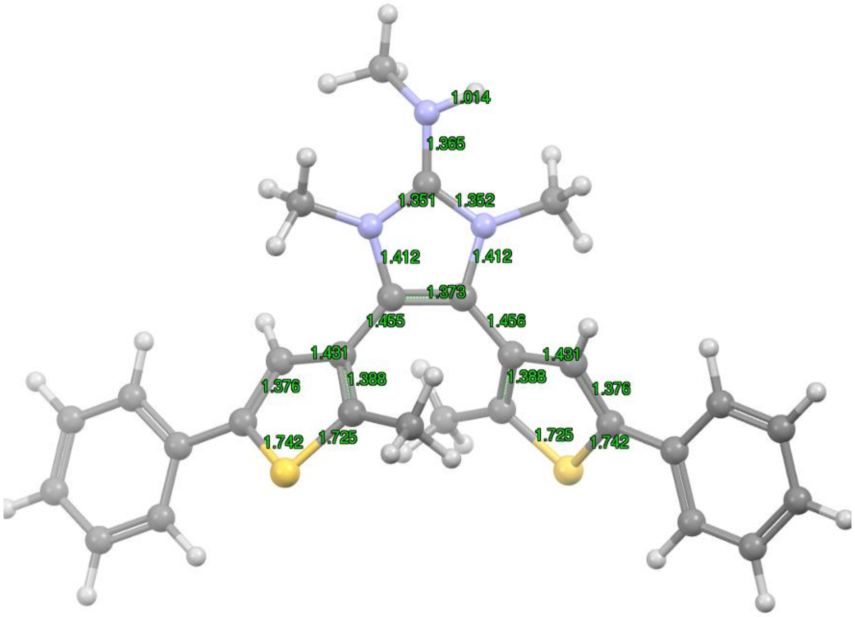

7o-H

Figure S46 (continued) Optimized molecular structures (TPSS-D3/def2-TZVP) of the imines, NHCs and protonated bases investigated in this DFT study.

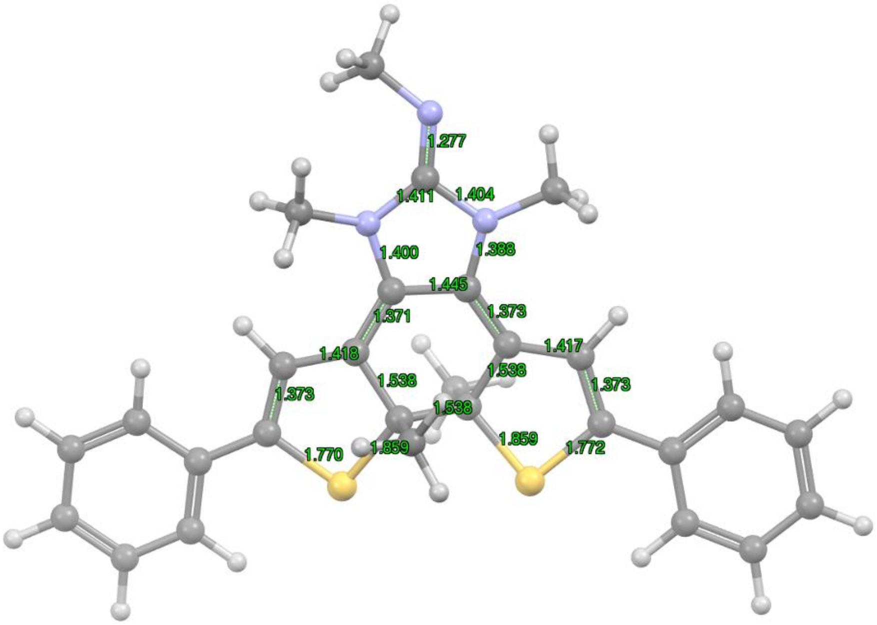

7c

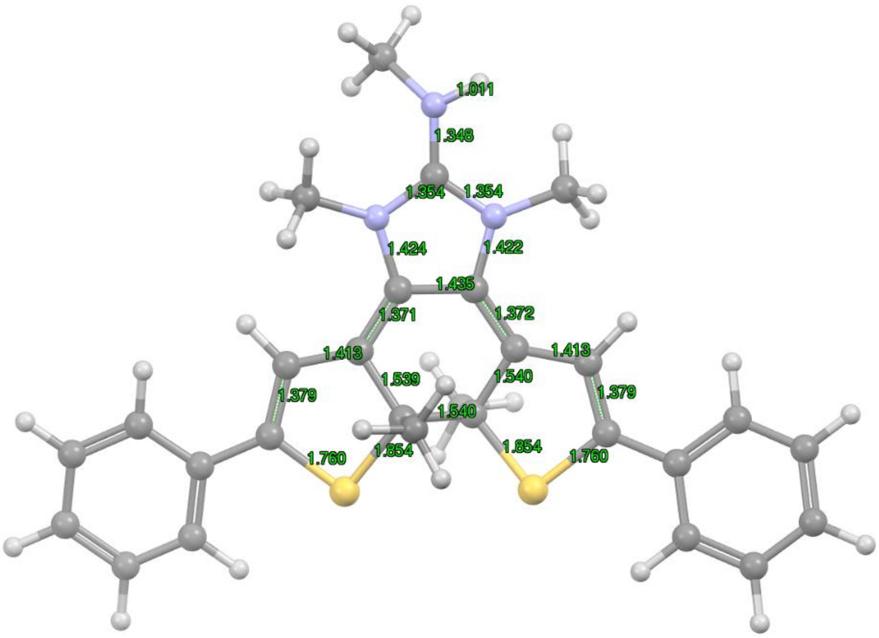

7c-H

Figure S46 (continued) Optimized molecular structures (TPSS-D3/def2-TZVP) of the imines, NHCs and protonated bases investigated in this DFT study.

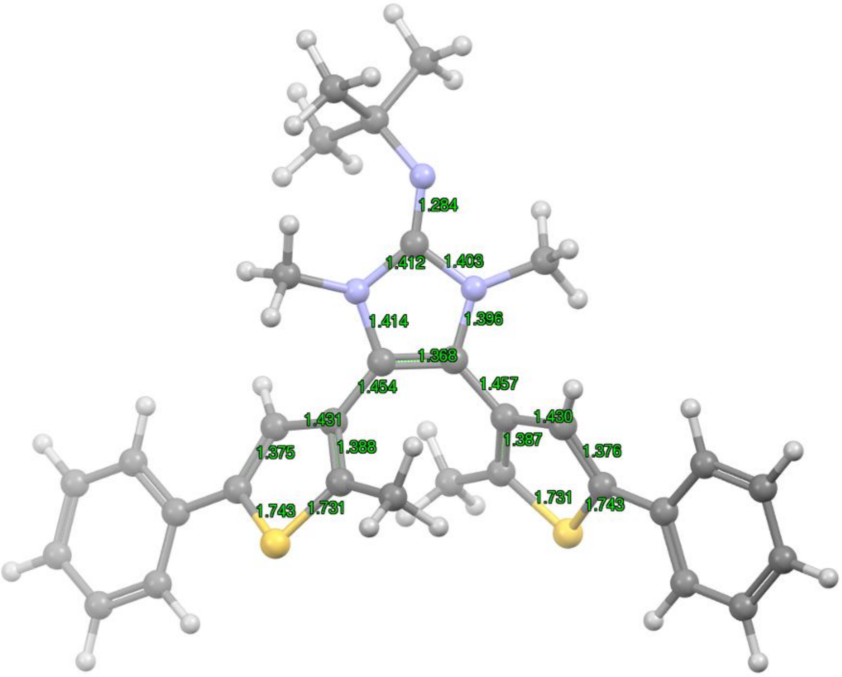

8o

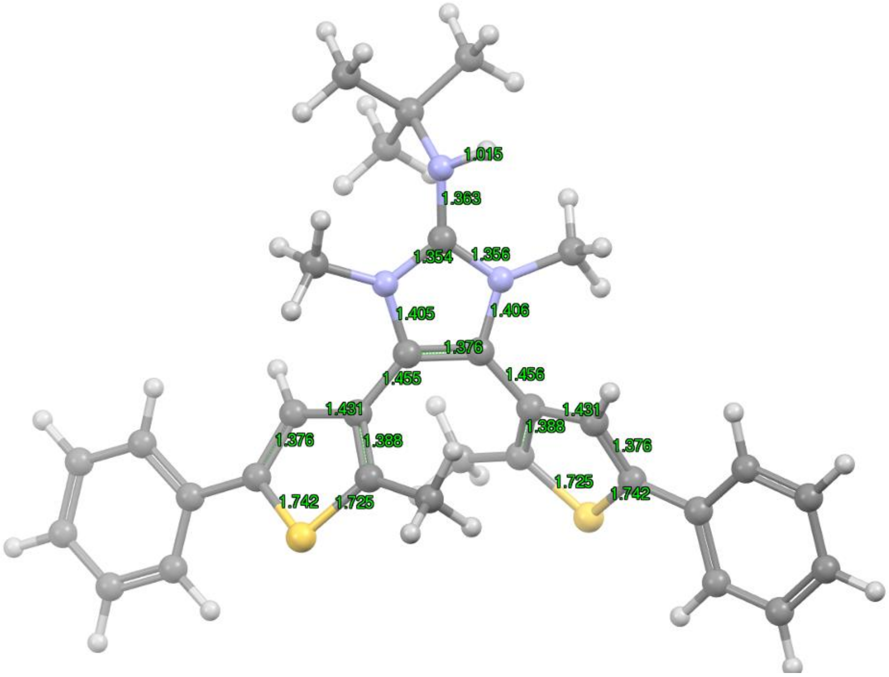

8o-H

Figure S46 (continued) Optimized molecular structures (TPSS-D3/def2-TZVP) of the imines, NHCs and protonated bases investigated in this DFT study.

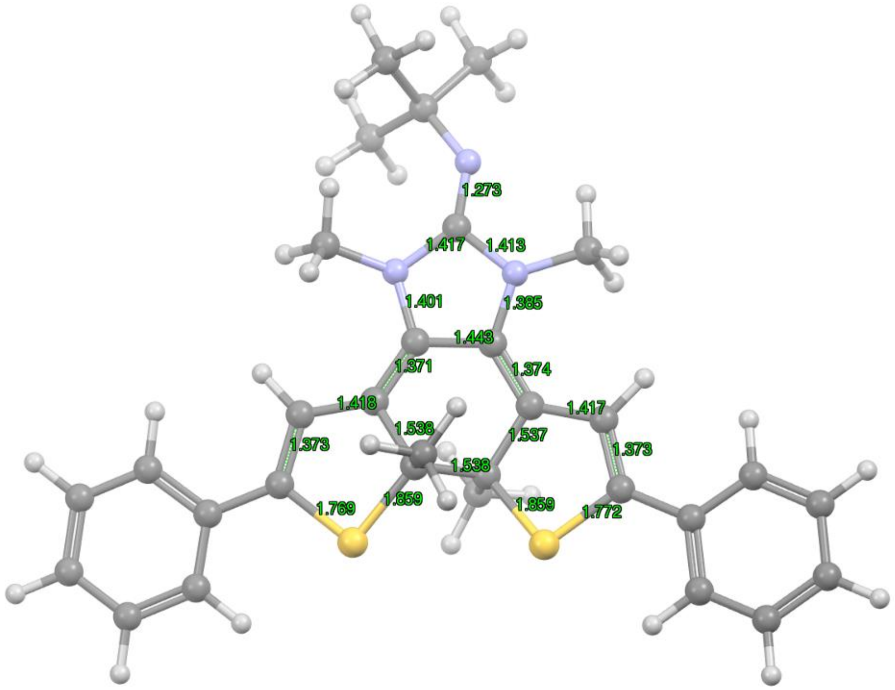

8c

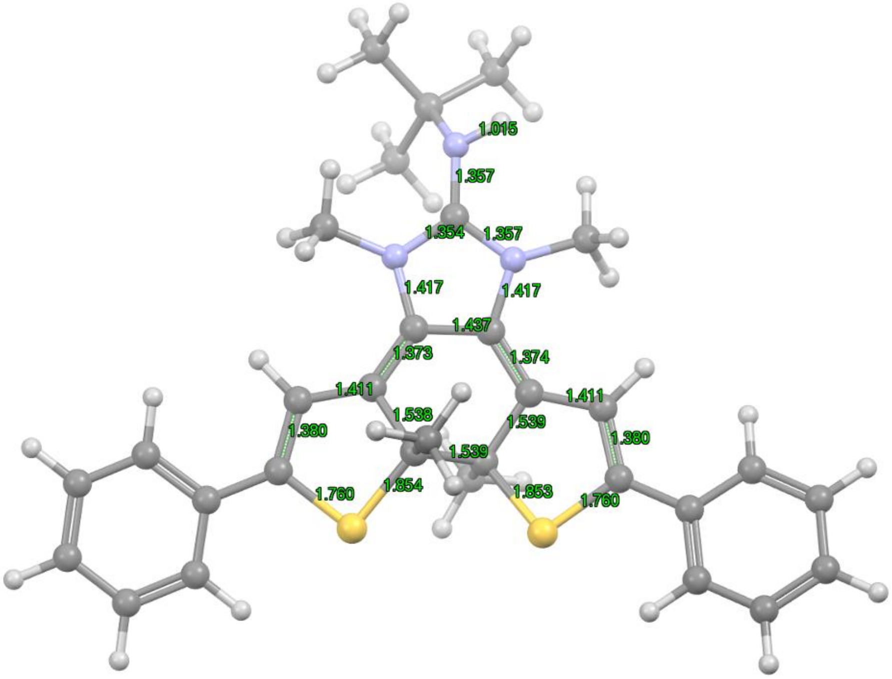

8c-H

Figure S46 (continued) Optimized molecular structures (TPSS-D3/def2-TZVP) of the imines, NHCs and protonated bases investigated in this DFT study.

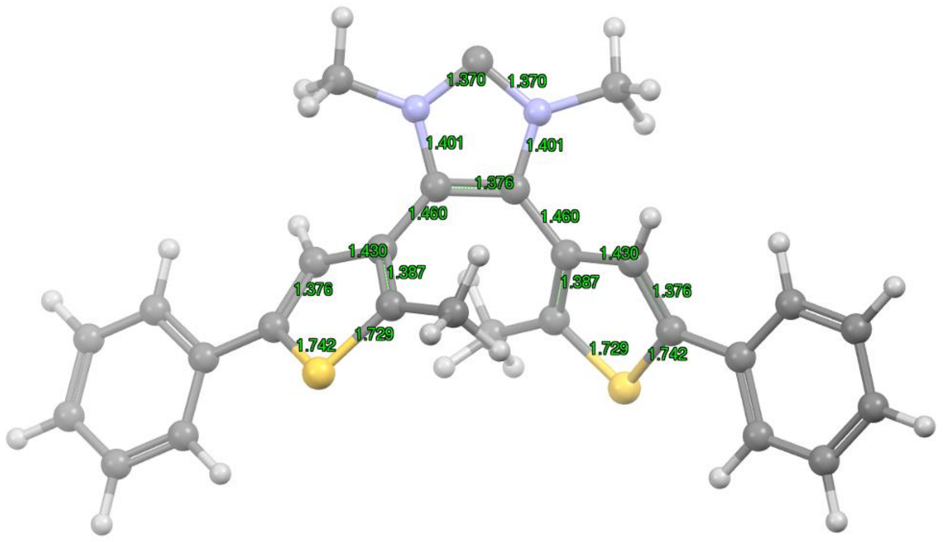

NHC-o

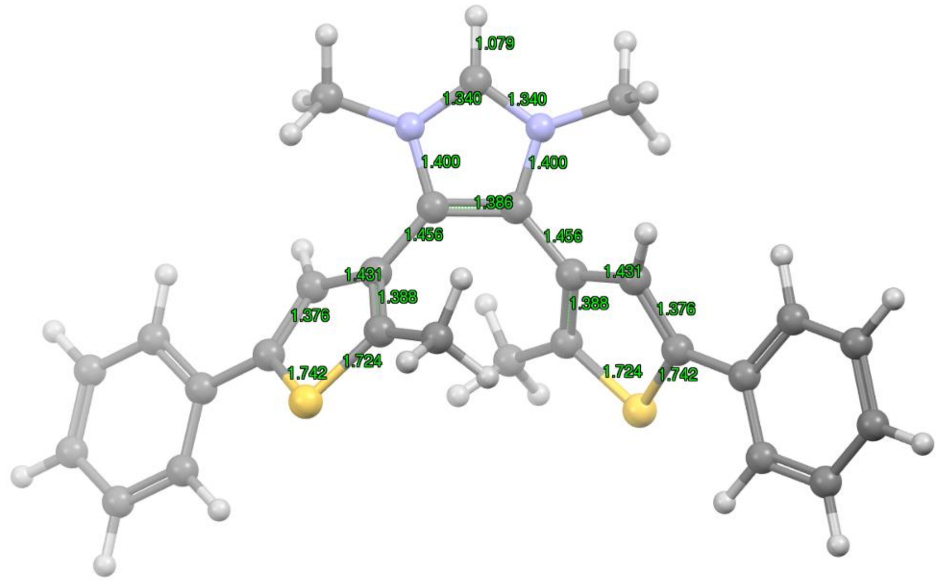

NHC-o-H

Figure S46 (continued) Optimized molecular structures (TPSS-D3/def2-TZVP) of the imines, NHCs and protonated bases investigated in this DFT study.

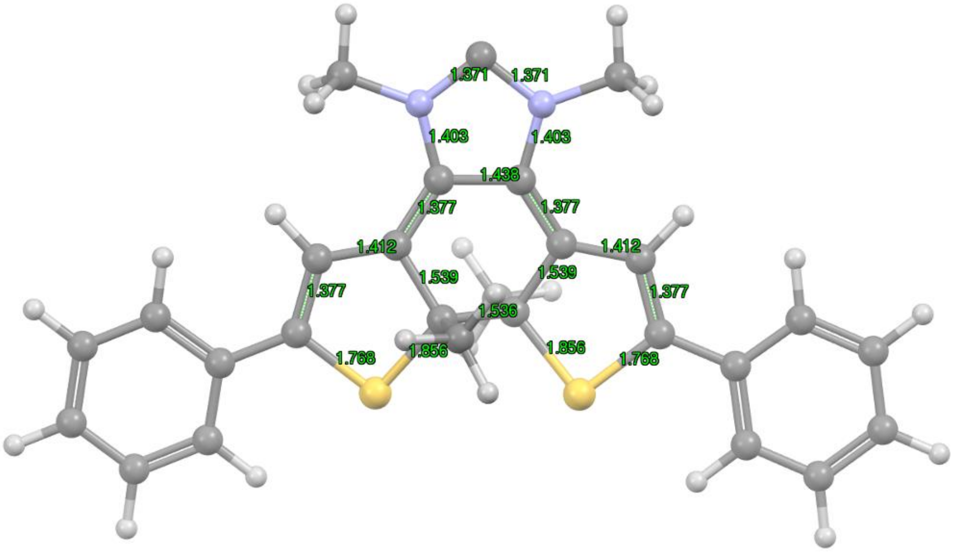

NHC-c

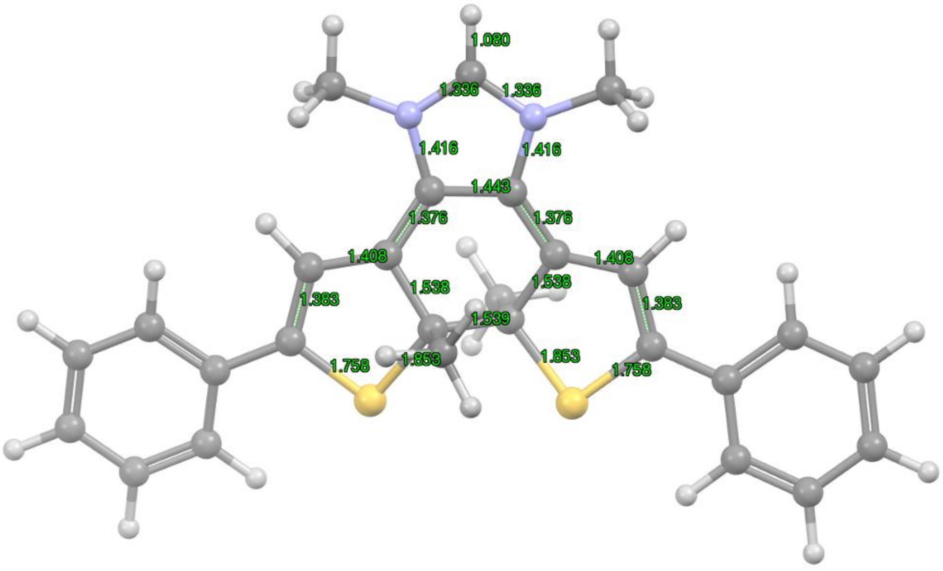

NHC-c-H

Figure S46 (continued) Optimized molecular structures (TPSS-D3/def2-TZVP) of the imines, NHCs and protonated bases investigated in this DFT study.

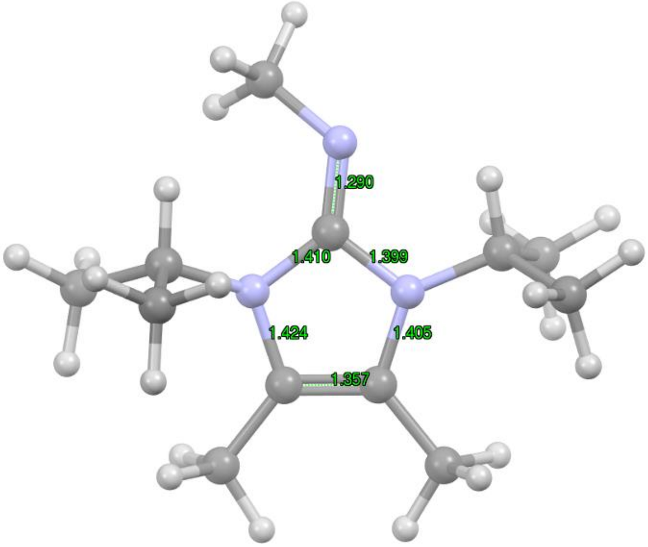

NHI^{Me}

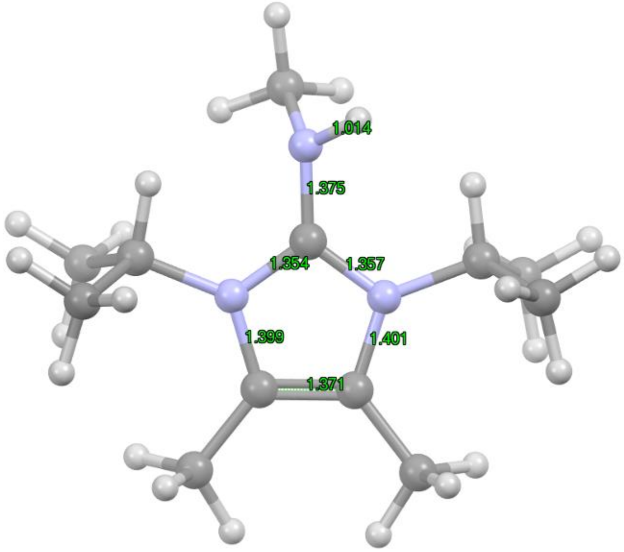

NHI^{Me}-H

Energies and Cartesian Coordinates of all structures reported in Table S5

7o

```
E(TPSS-D3/def2-TZVP) = -2044.732248016 (conv)
Lowest Freq. =    14.44 cm^-1
60
7o (001c1/opt)
C     0.1473239    1.3179084    0.5871185
C    -0.0680498    0.1982895    1.3450368
N    -0.1689973    0.6077656    2.6954667
C     0.0143027    2.0011965    2.7789354
N     0.2172298    2.4093188    1.4596732
C     0.2635592    3.8142566    1.1000216
C     0.1188295   -0.2986128    3.8065878
N     0.0010236    2.8380271    3.7553827
C    -0.4709013    2.4526535    5.0747149
C     0.2486102    1.4559837   -0.8592708
C    -0.1974377   -1.1930473    0.9450728
C    -0.6386901    0.8815199   -1.7579659
S    -0.1439955    1.1939011   -3.3866746
C     1.2483642    2.1156251   -2.8874518
C     1.3063720    2.1618112   -1.5138156
C    -1.2464689   -2.0485180    1.4101998
C    -1.2253773   -3.3128049    0.8689070
S     0.1194293   -3.4596220   -0.2301626
C     0.6471933   -1.8353395    0.0494879
C    -1.8791619    0.0942566   -1.4776890
C     1.8708092   -1.3059631   -0.6278772
C    -2.1458634   -4.4243570    1.1012735
C    -2.2625852   -5.4836270    0.1844240
C    -3.1456358   -6.5345317    0.4145802
C    -3.9394307   -6.5493950    1.5622996
C    -3.8339893   -5.5035680    2.4816430
C    -2.9449969   -4.4569631    2.2588578
C     2.1648106    2.7039548   -3.8625399
C     2.2360576    2.2266268   -5.1829035
C     3.1162134    2.7922085   -6.1009184
C     3.9519566    3.8431084   -5.7204689
C     3.8916168    4.3273461   -4.4120469
C     3.0057957    3.7704649   -3.4952008
H    -0.1750218    3.9467607    0.1085707
H    -0.3009913    4.3640771    1.8567286
H     1.2923777    4.1933155    1.0955710
H     0.5169823   -1.2287853    3.3962962
H     0.8676668    0.1525066    4.4604889
H    -0.7765950   -0.5271667    4.3918474
```

```
H    -0.8843216    3.3424577    5.5617836
H    -1.2635856    1.6882975    5.0569823
H     0.3383332    2.0848137    5.7230841
H     2.1124605    2.6467093   -0.9748155
H    -2.0203624   -1.7102045    2.0904052
H    -2.2529199    0.3467012   -0.4812939
H    -2.6597496    0.3072155   -2.2149418
H    -1.6858322   -0.9853554   -1.4895168
H     2.2859460   -0.4844776   -0.0364850
H     2.6317357   -2.0850487   -0.7390680
H     1.6448490   -0.9070369   -1.6243310
H    -1.6654834   -5.4707493   -0.7240644
H    -3.2192556   -7.3413277   -0.3092343
H    -4.6307028   -7.3677862    1.7402932
H    -4.4404021   -5.5086898    3.3831547
H    -2.8520451   -3.6635237    2.9946322
H     1.6055515    1.3935099   -5.4835466
H     3.1545144    2.4056313   -7.1154708
H     4.6406253    4.2824669   -6.4360193
H     4.5306029    5.1513979   -4.1070059
H     2.9477066    4.1744174   -2.4887422
```

7o-H

```
E(TPSS-D3/def2-TZVP) = -2045.156896339 (conv)
Lowest Freq. =    14.81 cm^-1
61
7o-H (001c1_H/opt)
C     0.1938631    1.2359869    0.5556875
C     0.0133773    0.1060374    1.3146229
N     0.0069400    0.5159940    2.6659059
C     0.1686676    1.8560722    2.7240280
N     0.2807004    2.3184195    1.4586970
C     0.3478251    3.7328289    1.0868011
C     0.0893049   -0.3901543    3.8147361
N     0.2813720    2.5975905    3.8639200
C    -0.7640673    2.5735762    4.9115543
C     0.2610148    1.4079614   -0.8881720
C    -0.1321525   -1.2874651    0.9214671
C    -0.6720809    0.8687036   -1.7624228
S    -0.2255489    1.2113326   -3.3932574
C     1.2158639    2.0731172   -2.9299934
C     1.3255309    2.0908183   -1.5580836
C    -1.1985148   -2.1288429    1.3715482
C    -1.1759316   -3.3902538    0.8218684
S     0.1873166   -3.5389956   -0.2526619
```

C	0.7162994	-1.9203728	0.0238667
C	-1.9117195	0.0886716	-1.4548361
C	1.9341423	-1.3848692	-0.6616744
C	-2.1041884	-4.5012028	1.0304730
C	-2.2453244	-5.5203923	0.0738707
C	-3.1363141	-6.5693123	0.2798787
C	-3.9081512	-6.6173982	1.4414054
C	-3.7765365	-5.6107409	2.3999793
C	-2.8803118	-4.5656642	2.2009996
C	2.1147372	2.6454015	-3.9318831
C	2.1439391	2.1543488	-5.2479196
C	3.0080660	2.7021373	-6.1912103
C	3.8640355	3.7458329	-5.8372709
C	3.8440369	4.2430030	-4.5326635
C	2.9751625	3.7034211	-3.5898579
H	0.0056943	3.8227055	0.0559566
H	-0.3097443	4.3096353	1.7416666
H	1.3727313	4.1073984	1.1592073
H	0.4817982	-1.3420635	3.4570796
H	0.7682596	0.0442266	4.5511266
H	-0.8958999	-0.5523890	4.2587068
H	-0.3981047	3.1594570	5.7549627
H	-1.7110228	2.9925760	4.5531537
H	-0.9285937	1.5514298	5.2512983
H	2.1755917	2.5263035	-1.0442226
H	-1.9889545	-1.7921688	2.0333830
H	-2.2628756	0.3228704	-0.4459431
H	-2.7119055	0.3200069	-2.1633763
H	-1.7253580	-0.9909831	-1.4961869
H	2.3625884	-0.5638045	-0.0801280
H	2.6956252	-2.1607942	-0.7796754
H	1.6936756	-0.9933189	-1.6571513
H	-1.6659204	-5.4794633	-0.8452256
H	-3.2332061	-7.3471186	-0.4714236
H	-4.6043914	-7.4348637	1.6007668
H	-4.3659505	-5.6473710	3.3113271
H	-2.7625570	-3.8042621	2.9668920
H	1.4980624	1.3256277	-5.5273677
H	3.0181324	2.3084362	-7.2029989
H	4.5392460	4.1714524	-6.5730214
H	4.4997566	5.0621206	-4.2528038
H	2.9456894	4.1176163	-2.5859891
H	0.6662796	3.5211474	3.7005833

7c

E(TPSS-D3/def2-TZVP) = -2044.715083832 (conv)

Lowest Freq. = 12.57 cm^-1

60

7c (002c2/opt)

C	0.1202510	1.3694768	0.8661683
C	-0.3439141	0.2405652	1.6391129
N	-0.5677860	0.7113684	2.9251968
C	-0.1745807	2.0545083	3.0419939
N	0.2283025	2.4511527	1.7487365
C	0.1583788	3.8317588	1.2786881
C	-0.8582193	-0.0984825	4.0941326
N	-0.1965900	2.6613362	4.1654601
C	0.4104873	3.9637223	4.3735172
C	0.3301818	1.2977261	-0.4870249
C	-0.4566644	-1.0171579	1.0991522
C	-0.1896460	0.0231430	-1.1728378
S	0.6645059	0.0034910	-2.8241966
C	1.1339977	1.7026454	-2.6686530
C	0.9119290	2.2039137	-1.4098023
C	-0.9803517	-2.2302916	1.6114859
C	-1.1010467	-3.2518272	0.7015386
S	-0.6008677	-2.7767307	-0.9304308
C	0.1514585	-1.1980224	-0.3014317
C	-1.7021671	0.1878565	-1.4301187
C	1.6694007	-1.4320205	-0.1469175
C	-1.6130303	-4.5924903	0.9358427
C	-1.7240037	-5.5161514	-0.1221161
C	-2.2272369	-6.7961173	0.0908468
C	-2.6302031	-7.1913631	1.3663179
C	-2.5231047	-6.2883075	2.4291496
C	-2.0227885	-5.0100610	2.2200137
C	1.7280517	2.3922051	-3.8036359
C	1.9825291	1.7082644	-5.0084041
C	2.5620727	2.3573442	-6.0945497
C	2.9013915	3.7075279	-6.0088879
C	2.6522154	4.4029326	-4.8212078
C	2.0735457	3.7584007	-3.7358704
H	-0.4406984	3.8600978	0.3633272
H	-0.3265892	4.4449185	2.0352533
H	1.1538298	4.2375711	1.0700872
H	-0.1292967	-0.9115804	4.1852022
H	-0.7921059	0.5636177	4.9584091
H	-1.8666531	-0.5190566	4.0269153
H	0.7683901	4.0073624	5.4072541
H	1.2648063	4.1688807	3.7122574

H	-0.3199747	4.7774416	4.2568392
H	1.2215894	3.2059573	-1.1385785
H	-1.3054512	-2.3452269	2.6380690
H	-2.2329157	0.2583849	-0.4747206
H	-1.8645230	1.1068241	-1.9988187
H	-2.0996851	-0.6595627	-1.9923710
H	2.1400841	-0.5380868	0.2760351
H	1.8266108	-2.2735195	0.5322356
H	2.1332013	-1.6537378	-1.1103015
H	-1.4057969	-5.2223530	-1.1189418
H	-2.3033918	-7.4876967	-0.7435068
H	-3.0202884	-8.1908545	1.5341308
H	-2.8301767	-6.5862691	3.4279099
H	-1.9438781	-4.3271220	3.0602425
H	1.7204771	0.6564919	-5.0877460
H	2.7481159	1.8057141	-7.0117195
H	3.3505535	4.2158020	-6.8569578
H	2.9084887	5.4560099	-4.7445728
H	1.8787791	4.3196763	-2.8272016

7c-H

E(TPSS-D3/def2-TZVP) = -2045.123797245 (conv)

Lowest Freq. = 18.25 cm^-1

61

7c-H (002c2_H/opt)

C	0.0845537	1.3392889	0.7786673
C	-0.3654239	0.2155442	1.5491100
N	-0.5761876	0.7068172	2.8668084
C	-0.2750492	2.0262334	2.8976170
N	0.1224942	2.4460961	1.6737514
C	0.1047029	3.8406702	1.2237559
C	-0.7795253	-0.1373788	4.0402697
N	-0.3763956	2.8078310	3.9911198
C	0.5515606	3.9019474	4.3304971
C	0.3272152	1.2790818	-0.5695679
C	-0.4666901	-1.0546628	1.0407369
C	-0.1613117	-0.0190225	-1.2369638
S	0.7069520	-0.0519011	-2.8748617
C	1.1431414	1.6482398	-2.7463388
C	0.8974516	2.1788484	-1.4972667
C	-1.0039982	-2.2642387	1.5344011
C	-1.0805670	-3.2870455	0.6122894
S	-0.5077512	-2.8227556	-0.9855312
C	0.1823275	-1.2260760	-0.3451806
C	-1.6758581	0.1280757	-1.5114976

C	1.7021857	-1.4207131	-0.1377589
C	-1.6032147	-4.6270656	0.8269041
C	-1.8023486	-5.5002060	-0.2596609
C	-2.3157181	-6.7772912	-0.0636351
C	-2.6378514	-7.2127692	1.2224513
C	-2.4393599	-6.3608018	2.3130713
C	-1.9274755	-5.0846562	2.1206104
C	1.7356752	2.3269046	-3.8879682
C	2.1382208	1.5992397	-5.0241495
C	2.7180659	2.2407085	-6.1128179
C	2.9043770	3.6236336	-6.0931499
C	2.5030950	4.3617630	-4.9757000
C	1.9246793	3.7241326	-3.8863682
H	-0.4411820	3.8806245	0.2791434
H	-0.4164524	4.4492854	1.9619489
H	1.1194196	4.2190716	1.0778134
H	-0.1720602	-1.0374740	3.9276594
H	-0.4480310	0.3962657	4.9332408
H	-1.8323967	-0.4129064	4.1444268
H	0.9092927	3.7545676	5.3520815
H	1.4082808	3.8705870	3.6567855
H	0.0604344	4.8755415	4.2586917
H	1.1981263	3.1894065	-1.2491772
H	-1.4015194	-2.3833365	2.5347115
H	-2.2191341	0.2181320	-0.5649283
H	-1.8415506	1.0293725	-2.1058653
H	-2.0609053	-0.7354254	-2.0561738
H	2.1371684	-0.5172156	0.3021394
H	1.8616022	-2.2615277	0.5409584
H	2.2031168	-1.6256714	-1.0852114
H	-1.5576256	-5.1690014	-1.2656153
H	-2.4642243	-7.4342244	-0.9149857
H	-3.0359615	-8.2108507	1.3767702
H	-2.6794395	-6.6985548	3.3166909
H	-1.7611782	-4.4434826	2.9807226
H	2.0016196	0.5211118	-5.0465312
H	3.0247667	1.6614685	-6.9781787
H	3.3535084	4.1256987	-6.9444528
H	2.6361258	5.4392768	-4.9603154
H	1.5986016	4.3144268	-3.0355267
H	-0.9323745	2.4363618	4.7495333

8o

```
E(TPSS-D3/def2-TZVP) = -2162.743685924 (conv)
Lowest Freq. =    15.59 cm^-1
69
8o (003c2/opt)
C     0.2910270    0.9142810   -0.1418493
C     0.0974151   -0.2363716    0.5725838
N     0.2268230    0.0816408    1.9443187
C     0.5164607    1.4560749    2.0917953
N     0.5612616    1.9331788    0.7730810
C     0.6535128    3.3521845    0.4871416
C     0.6243608   -0.9388242    2.9073196
N     0.7240373    2.2577142    3.0730091
C     0.3351777    2.1510736    4.4795039
C     0.2056011    1.1390641   -1.5785368
C    -0.1821470   -1.5888813    0.1192479
C    -0.8369336    0.6849857   -2.3732181
S    -0.5458632    1.0660526   -4.0360475
C     0.9685319    1.8554187   -3.6884286
C     1.2159396    1.8104483   -2.3359326
C    -1.2148378   -2.4058328    0.6798266
C    -1.3530691   -3.6340016    0.0762127
S    -0.1821841   -3.7947394   -1.2049652
C     0.4870446   -2.2248242   -0.9179316
C    -2.0830880   -0.0341453   -1.9644902
C     1.6408716   -1.7297617   -1.7302298
C    -2.3072240   -4.7000400    0.3759499
C    -2.6125472   -5.6974434   -0.5665717
C    -3.5254421   -6.7055231   -0.2706804
C    -4.1618086   -6.7377734    0.9709258
C    -3.8683382   -5.7532117    1.9167488
C    -2.9488825   -4.7502198    1.6271272
C     1.7869820    2.4385192   -4.7500030
C     1.6394306    2.0421917   -6.0906970
C     2.4280053    2.6011008   -7.0922024
C     3.3884171    3.5636596   -6.7779999
C     3.5456910    3.9667916   -5.4502697
C     2.7519804    3.4170676   -4.4486409
H     0.1216705    3.5653947   -0.4425179
H     0.2084506    3.8874892    1.3290416
H     1.6971678    3.6742290    0.3918078
H     1.1757872   -1.7251047    2.3843677
H     1.2719565   -0.4801860    3.6533820
H    -0.2361147   -1.3935479    3.4080117
H     2.1225022    2.2035783   -1.8897621
H    -1.8663925   -2.0626401    1.4757496
```

```
H    -2.2969194    0.1770655   -0.9129272
H    -2.9389814    0.2778272   -2.5713922
H    -1.9746053   -1.1211601   -2.0629366
H     2.1893030   -0.9748244   -1.1590108
H     2.3234222   -2.5454025   -1.9894132
H     1.3098131   -1.2548790   -2.6618450
H    -2.1394574   -5.6691817   -1.5450059
H    -3.7459836   -7.4648178   -1.0157284
H    -4.8763506   -7.5226333    1.2006046
H    -4.3508403   -5.7729364    2.8900271
H    -2.7087774   -4.0060246    2.3808522
H     0.9099400    1.2766824   -6.3432523
H     2.2967731    2.2780711   -8.1211153
H     4.0056377    3.9975630   -7.5590656
H     4.2836792    4.7221798   -5.1949490
H     2.8639449    3.7591405   -3.4239919
C    -0.9507096    1.3351412    4.7185959
H    -1.7453595    1.6851388    4.0509864
H    -1.2894184    1.4672614    5.7525493
H    -0.8072014    0.2657201    4.5535887
C     1.4944956    1.6232023    5.3495561
H     1.2581923    1.7394879    6.4138282
H     2.4051880    2.1888902    5.1303235
H     1.6996954    0.5638162    5.1677308
C     0.0562461    3.6067268    4.9167014
H    -0.7758524    4.0183858    4.3364066
H     0.9404897    4.2233713    4.7304324
H    -0.1975968    3.6571213    5.9822729
```

8o-H

```
E(TPSS-D3/def2-TZVP) = -2163.176655352 (conv)
Lowest Freq. =    16.98 cm^-1
70
8o-H (003c2_H/opt)
C     0.4372416    0.8028145   -0.1114191
C     0.2959371   -0.3780000    0.5815828
N     0.5705979   -0.0929858    1.9300345
C     0.8509120    1.2242246    2.0691974
N     0.7754709    1.7850050    0.8364137
C     0.9092940    3.2160825    0.5626732
C     0.7719189   -1.0992040    2.9740057
N     1.2030246    1.8454006    3.2304217
C     0.2271343    2.1326180    4.3683510
C     0.2598599    1.0959098   -1.5267152
C    -0.0423212   -1.7150311    0.1162608
```

C	-0.8583980	0.7045459	-2.2492294
S	-0.6854823	1.1594040	-3.9045191
C	0.8844446	1.8716416	-3.6550631
C	1.2403590	1.7592633	-2.3303013
C	-1.0788468	-2.5132027	0.6953977
C	-1.2692349	-3.7179493	0.0582710
S	-0.1433611	-3.8689361	-1.2625745
C	0.5685841	-2.3246547	-0.9702752
C	-2.0859242	-0.0081371	-1.7746039
C	1.6849723	-1.8209787	-1.8304630
C	-2.2386872	-4.7720672	0.3555363
C	-2.6464778	-5.6850009	-0.6314107
C	-3.5738094	-6.6803988	-0.3383192
C	-4.1164080	-6.7796000	0.9434530
C	-3.7188786	-5.8784231	1.9330833
C	-2.7855935	-4.8877939	1.6455289
C	1.6343830	2.4664538	-4.7610382
C	1.3904575	2.0883584	-6.0919601
C	2.1151444	2.6560556	-7.1355861
C	3.1013666	3.6069495	-6.8702031
C	3.3526756	3.9915639	-5.5517010
C	2.6238765	3.4324156	-4.5070786
H	0.3321941	3.4430006	-0.3337144
H	0.5151324	3.7763355	1.4122366
H	1.9557412	3.4845661	0.3944410
H	1.0884542	-2.0236505	2.4906566
H	1.5450280	-0.7312430	3.6496948
H	-0.1505697	-1.2812687	3.5294794
H	2.2028836	2.0870909	-1.9532570
H	-1.7020681	-2.1808748	1.5183042
H	-2.2283144	0.1606293	-0.7034636
H	-2.9770534	0.3425026	-2.3026329
H	-2.0053331	-1.0905376	-1.9294312
H	2.2821012	-1.0880268	-1.2807425
H	2.3415538	-2.6368643	-2.1452111
H	1.3043138	-1.3266320	-2.7319840
H	-2.2471437	-5.6013126	-1.6391459
H	-3.8787001	-7.3757889	-1.1144073
H	-4.8404273	-7.5557103	1.1712308
H	-4.1286431	-5.9563836	2.9357515
H	-2.4589195	-4.2113441	2.4303367
H	0.6407576	1.3309260	-6.3070453
H	1.9143649	2.3499307	-8.1577362
H	3.6676926	4.0480058	-7.6845407
H	4.1111783	4.7389139	-5.3384614
H	2.8054278	3.7608059	-3.4875934

```
C    -1.1933936    1.7401202    3.9545914
H    -1.5077667    2.2744049    3.0515176
H    -1.8839915    2.0020343    4.7604682
H    -1.2886428    0.6653045    3.7768059
C     0.6763270    1.3673950    5.6182134
H     0.0394705    1.6337022    6.4673756
H     1.7104545    1.6173731    5.8720443
H     0.6073523    0.2866758    5.4713461
C     0.3013018    3.6437824    4.6253984
H    -0.0568246    4.2117713    3.7601372
H     1.3288434    3.9512367    4.8522996
H    -0.3201888    3.9077328    5.4853126
H     1.8431756    2.6190535    3.0839749
```

8c

```
E(TPSS-D3/def2-TZVP) = -2162.726206189 (conv)
Lowest Freq. =    14.83 cm^-1
69
8c (004c1/opt)
C     0.3605920    1.0361850    0.0712002
C    -0.1104052   -0.1100350    0.8108073
N    -0.3670898    0.3205994    2.1190540
C    -0.0529806    1.6966114    2.2463501
N     0.4404120    2.0795988    0.9784178
C     0.6816047    3.4707250    0.6474372
C    -0.3003935   -0.6206577    3.2292057
N    -0.1346004    2.5551866    3.1827038
C    -0.8911158    2.5720587    4.4367222
C     0.6027814    0.9843363   -1.2804026
C    -0.1929656   -1.3609638    0.2548938
C     0.1279580   -0.2924132   -1.9933833
S     1.0313132   -0.2773695   -3.6176065
C     1.4286724    1.4406397   -3.4432849
C     1.1651376    1.9201667   -2.1835707
C    -0.7417349   -2.5802221    0.7279987
C    -0.8211951   -3.5901666   -0.1988419
S    -0.2434248   -3.1019606   -1.7984473
C     0.4632073   -1.5187830   -1.1271428
C    -1.3793524   -0.1524787   -2.2944128
C     1.9786133   -1.7307032   -0.9264989
C    -1.3493676   -4.9315554   -0.0025172
C    -1.4593722   -5.8269841   -1.0842184
C    -1.9771052   -7.1067125   -0.9077989
C    -2.3968304   -7.5287036    0.3536579
C    -2.2900457   -6.6540419    1.4398592
```

C	-1.7735561	-5.3766349	1.2672261
C	2.0090793	2.1626849	-4.5638976
C	2.2523111	1.5139019	-5.7906439
C	2.8207698	2.1941490	-6.8635482
C	3.1603745	3.5415964	-6.7432413
C	2.9228190	4.2022949	-5.5333836
C	2.3562910	3.5266992	-4.4607712
H	0.0105221	3.7926188	-0.1568518
H	0.4878522	4.0443469	1.5547752
H	1.7206947	3.6159867	0.3343597
H	0.4427795	-1.3906574	2.9962249
H	0.0072668	-0.0869789	4.1253586
H	-1.2673796	-1.1007001	3.4102180
H	1.4123875	2.9361025	-1.9016791
H	-1.1292478	-2.7036963	1.7318654
H	-1.9407775	-0.1018946	-1.3552442
H	-1.5399404	0.7708649	-2.8563225
H	-1.7443280	-0.9992450	-2.8794288
H	2.4194994	-0.8343022	-0.4777274
H	2.1291132	-2.5791674	-0.2543593
H	2.4767679	-1.9302997	-1.8773819
H	-1.1330980	-5.5102441	-2.0713612
H	-2.0517983	-7.7768826	-1.7595227
H	-2.7988390	-8.5278757	0.4928279
H	-2.6086565	-6.9740513	2.4281094
H	-1.6890250	-4.7175428	2.1257961
H	1.9854383	0.4659170	-5.8987919
H	2.9973111	1.6689328	-7.7980256
H	3.6023665	4.0738080	-7.5803509
H	3.1804810	5.2526646	-5.4286308
H	2.1772986	4.0603611	-3.5324814
C	-2.2091157	1.7746081	4.3924266
H	-2.7841290	2.0488627	3.5019601
H	-2.8119955	2.0145610	5.2750855
H	-2.0539658	0.6947625	4.3840620
C	-1.2484940	4.0600288	4.6499029
H	-1.8820940	4.4148089	3.8308757
H	-0.3358154	4.6628359	4.6638730
H	-1.7842613	4.2005811	5.5955127
C	-0.0061100	2.1327974	5.6199644
H	-0.5240240	2.3214256	6.5668569
H	0.9285771	2.7013741	5.6139006
H	0.2443374	1.0682402	5.5822820

8c-H

E(TPSS-D3/def2-TZVP) = -2163.143060443 (conv)

Lowest Freq. =   15.63 cm^-1

```
70
8c-H (004c1_H/opt)
C     0.4945548    0.9239716    0.1240582
C     0.0508539   -0.2313183    0.8539389
N    -0.0764095    0.1810563    2.2030585
C     0.2576809    1.4894887    2.3033274
N     0.6106505    1.9623438    1.0812600
C     0.8396536    3.3649420    0.7532908
C    -0.1367547   -0.7409407    3.3342555
N     0.3007564    2.1920294    3.4638133
C    -0.9102036    2.5588514    4.3172466
C     0.6610807    0.9345605   -1.2394670
C    -0.1083448   -1.4683970    0.2795190
C     0.1004917   -0.3120992   -1.9475532
S     0.8730444   -0.2737072   -3.6319478
C     1.3485287    1.4098107   -3.4406247
C     1.1922333    1.8730634   -2.1502399
C    -0.6420798   -2.6914594    0.7387996
C    -0.7926814   -3.6615691   -0.2309829
S    -0.3070701   -3.1178918   -1.8329783
C     0.4596168   -1.5760384   -1.1461463
C    -1.4229244   -0.1120489   -2.1249145
C     1.9831165   -1.8183850   -1.0346549
C    -1.3235901   -5.0038127   -0.0568286
C    -1.5939415   -5.8177732   -1.1737201
C    -2.1136427   -7.0973823   -1.0146657
C    -2.3712262   -7.5948120    0.2635369
C    -2.1020998   -6.8021095    1.3833655
C    -1.5838837   -5.5235083    1.2280289
C     1.8790039    2.1417977   -4.5793535
C     2.1445657    1.4814518   -5.7944448
C     2.6648351    2.1722284   -6.8829967
C     2.9280516    3.5391494   -6.7843595
C     2.6637982    4.2113504   -5.5872454
C     2.1450179    3.5243194   -4.4981110
H     0.2066505    3.6359512   -0.0948001
H     0.5688628    3.9822343    1.6103903
H     1.8892483    3.5318666    0.4974198
H     0.5321516   -1.5798983    3.1284523
H     0.1983150   -0.2095239    4.2244142
H    -1.1537494   -1.1119955    3.4822484
H     1.5212982    2.8656449   -1.8692825
H    -0.9764520   -2.8573759    1.7554169
```

```
H    -1.9090284   -0.0604096   -1.1450208
H    -1.5960887    0.8243478   -2.6599015
H    -1.8623093   -0.9346717   -2.6912709
H     2.4651616   -0.9535342   -0.5670443
H     2.1556814   -2.7015738   -0.4156458
H     2.4273174   -1.9780992   -2.0183918
H    -1.3987945   -5.4383601   -2.1734637
H    -2.3171119   -7.7081932   -1.8887037
H    -2.7739052   -8.5951442    0.3887586
H    -2.2919799   -7.1882363    2.3802733
H    -1.3624566   -4.9290586    2.1089904
H     1.9445729    0.4165485   -5.8807695
H     2.8646075    1.6439457   -7.8100531
H     3.3317502    4.0799055   -7.6347163
H     2.8585588    5.2766239   -5.5082883
H     1.9290508    4.0660239   -3.5824579
C    -2.1847599    1.9494642    3.7311570
H    -2.3299096    2.2511505    2.6885146
H    -3.0390453    2.3117503    4.3089816
H    -2.1893322    0.8590454    3.7877325
C    -1.0060963    4.0917045    4.2838000
H    -1.2176447    4.4521888    3.2716530
H    -0.0763759    4.5510776    4.6400825
H    -1.8132602    4.4288401    4.9399775
C    -0.6635616    2.0779986    5.7509202
H    -1.4796341    2.4102834    6.3995254
H     0.2728955    2.4864848    6.1422282
H    -0.6136996    0.9869726    5.8034267
H     0.9763906    2.9488590    3.4391824
```

NHC-o

```
E(TPSS-D3/def2-TZVP) = -1949.984847561 (conv)
Lowest Freq. =    15.29 cm^-1
55
Biel-o (005c1/opt)
C     0.1435923    1.5637651    1.0964594
C    -0.0432501    0.4382805    1.8651866
N    -0.0618652    0.8874219    3.1923835
C     0.1079367    2.2419539    3.3118658
N     0.2328583    2.6284872    2.0031333
C     0.3951239    4.0273444    1.6258611
C    -0.1998895    0.0230922    4.3582494
C     0.2111176    1.6969042   -0.3555484
C    -0.1721176   -0.9565163    1.4545951
C    -0.7168306    1.1349762   -1.2203591
```

S	-0.2728493	1.4264089	-2.8661298
C	1.1586535	2.3143934	-2.4223576
C	1.2645914	2.3677438	-1.0512778
C	-1.2360691	-1.8185339	1.8655592
C	-1.1943489	-3.0736985	1.3028773
S	0.1889626	-3.1998793	0.2516288
C	0.6987355	-1.5787108	0.5718483
C	-1.9613411	0.3725948	-0.8909542
C	1.9344338	-1.0286741	-0.0678734
C	-2.1213017	-4.1894966	1.4825411
C	-2.2101826	-5.2274712	0.5385540
C	-3.1006566	-6.2820872	0.7173227
C	-3.9296947	-6.3216821	1.8392839
C	-3.8518319	-5.2974186	2.7852745
C	-2.9554727	-4.2472991	2.6141055
C	2.0567603	2.8704408	-3.4326622
C	2.0686925	2.3826863	-4.7511722
C	2.9320144	2.9169986	-5.7032143
C	3.8099974	3.9459861	-5.3597138
C	3.8088278	4.4402776	-4.0536712
C	2.9399923	3.9147939	-3.1026584
H	-0.2830788	4.2767098	0.8052215
H	0.1600292	4.6234201	2.5070183
H	1.4232757	4.2344257	1.3118102
H	0.4450905	-0.8537421	4.2545977
H	0.0951070	0.6111059	5.2266998
H	-1.2352993	-0.3107127	4.4813855
H	2.1048572	2.8305169	-0.5466295
H	-2.0386829	-1.4963904	2.5190040
H	-2.2912794	0.6319008	0.1188918
H	-2.7666693	0.6025796	-1.5956297
H	-1.7901753	-0.7103551	-0.9124829
H	2.3164790	-0.1977549	0.5316670
H	2.7136601	-1.7930123	-0.1493384
H	1.7315827	-0.6418516	-1.0737321
H	-1.5852497	-5.1948251	-0.3505056
H	-3.1523911	-7.0721881	-0.0264910
H	-4.6269755	-7.1427324	1.9769736
H	-4.4852465	-5.3226880	3.6676459
H	-2.8837748	-3.4716847	3.3709437
H	1.4049779	1.5659268	-5.0238277
H	2.9240041	2.5229541	-6.7155754
H	4.4857138	4.3606619	-6.1018202
H	4.4808671	5.2479119	-3.7771281
H	2.9285897	4.3267413	-2.0978358

NHC-o-H

E(TPSS-D3/def2-TZVP) = -1950.431619353 (conv)

Lowest Freq. =   18.03 cm^-1

```
56
Biel-o-H (005c1_H/opt)
C     0.0917802    0.6867549    1.6794779
C    -0.0917802   -0.6867549    1.6794779
N    -0.1501937   -1.0780970    3.0224719
C     0.0000000    0.0000000    3.8035788
N     0.1501937    1.0780970    3.0224719
C     0.3004109    2.4565763    3.5090702
C    -0.3004109   -2.4565763    3.5090702
C     0.1937073    1.6229259    0.5693312
C    -0.1937073   -1.6229259    0.5693312
C    -0.7088473    1.6470011   -0.4855350
S    -0.2308461    2.8288144   -1.6459157
C     1.1861094    3.2962713   -0.7466642
C     1.2623309    2.5617881    0.4146198
C    -1.2623309   -2.5617881    0.4146198
C    -1.1861094   -3.2962713   -0.7466642
S     0.2308461   -2.8288144   -1.6459157
C     0.7088473   -1.6470011   -0.4855350
C    -1.9442447    0.8273340   -0.6923639
C     1.9442447   -0.8273340   -0.6923639
C    -2.0994638   -4.3230885   -1.2472318
C    -2.1800422   -4.6178682   -2.6184764
C    -3.0578511   -5.5919569   -3.0842578
C    -3.8756977   -6.2850328   -2.1909506
C    -3.8040365   -6.0015873   -0.8256462
C    -2.9213522   -5.0346295   -0.3558454
C     2.0994638    4.3230885   -1.2472318
C     2.1800422    4.6178682   -2.6184764
C     3.0578511    5.5919569   -3.0842578
C     3.8756977    6.2850328   -2.1909506
C     3.8040365    6.0015873   -0.8256462
C     2.9213522    5.0346295   -0.3558454
H    -0.2329083    3.1195482    2.8275446
H    -0.1275327    2.5215917    4.5096188
H     1.3564579    2.7312902    3.5360804
H     0.2329083   -3.1195482    2.8275446
H     0.1275327   -2.5215917    4.5096188
H    -1.3564579   -2.7312902    3.5360804
H     2.0947462    2.6459569    1.1042376
H    -2.0947462   -2.6459569    1.1042376
H    -2.3267085    0.4693931    0.2675593
H    -2.7296192    1.4118125   -1.1794454
```

```
H    -1.7408325   -0.0509596   -1.3160155
H     2.3267085   -0.4693931    0.2675593
H     2.7296192   -1.4118125   -1.1794454
H     1.7408325    0.0509596   -1.3160155
H    -1.5641597   -4.0664167   -3.3246577
H    -3.1084987   -5.8053585   -4.1475517
H    -4.5614409   -7.0436728   -2.5550406
H    -4.4294440   -6.5451768   -0.1238355
H    -2.8496383   -4.8438232    0.7112223
H     1.5641597    4.0664167   -3.3246577
H     3.1084987    5.8053585   -4.1475517
H     4.5614409    7.0436728   -2.5550406
H     4.4294440    6.5451768   -0.1238355
H     2.8496383    4.8438232    0.7112223
H     0.0000000    0.0000000    4.8823177
```

NHC-c

```
E(TPSS-D3/def2-TZVP) = -1949.955738296 (conv)
Lowest Freq. =    13.51 cm^-1
55
Biel-c (006c1/opt)
C     0.2102625    1.6893138    1.2861026
C    -0.2769449    0.5650018    2.0387866
N    -0.4542328    1.0549448    3.3418867
C    -0.0746902    2.3643992    3.4838802
N     0.3363951    2.7233876    2.2265024
C     0.7916225    4.0707938    1.9247325
C    -0.9284703    0.2681838    4.4687822
C     0.4050811    1.6397482   -0.0758507
C    -0.4295444   -0.6861970    1.4850989
C    -0.1443647    0.3825351   -0.7719519
S     0.6936979    0.3698194   -2.4281322
C     1.1296706    2.0766628   -2.2757214
C     0.9427372    2.5620519   -1.0004957
C    -0.9607213   -1.8958455    1.9843119
C    -1.0990612   -2.9052963    1.0577226
S    -0.6180206   -2.4100916   -0.5700134
C     0.1677596   -0.8580731    0.0777488
C    -1.6566100    0.5955735   -1.0123626
C     1.6818995   -1.1284529    0.2340647
C    -1.6157092   -4.2453271    1.2813881
C    -1.7537193   -5.1519355    0.2116064
C    -2.2616761   -6.4310173    0.4155320
C    -2.6432518   -6.8414134    1.6931180
C    -2.5093956   -5.9556115    2.7672181
```

C	-2.0033592	-4.6781760	2.5674530
C	1.6613998	2.7912354	-3.4243535
C	1.8357246	2.1388139	-4.6610461
C	2.3569844	2.8125432	-5.7610827
C	2.7166228	4.1564932	-5.6578288
C	2.5472989	4.8205713	-4.4385641
C	2.0277709	4.1514233	-3.3385166
H	0.1359788	4.5414594	1.1839329
H	0.7569878	4.6337788	2.8570112
H	1.8170116	4.0539053	1.5398665
H	-0.2604788	-0.5801720	4.6538487
H	-0.9345533	0.9277292	5.3361108
H	-1.9409351	-0.1045932	4.2796068
H	1.2258144	3.5717405	-0.7304810
H	-1.2765718	-2.0221906	3.0123404
H	-2.1750850	0.6823430	-0.0519447
H	-1.7937211	1.5209405	-1.5769248
H	-2.0864763	-0.2366385	-1.5728446
H	2.1685468	-0.2573036	0.6845432
H	1.8168954	-1.9917594	0.8900572
H	2.1464549	-1.3329738	-0.7323320
H	-1.4535303	-4.8453332	-0.7869289
H	-2.3585357	-7.1101667	-0.4267086
H	-3.0376888	-7.8403584	1.8536368
H	-2.7999006	-6.2663610	3.7669070
H	-1.9016431	-4.0088677	3.4159861
H	1.5523882	1.0938031	-4.7550152
H	2.4811472	2.2858927	-6.7029985
H	3.1218220	4.6837392	-6.5163688
H	2.8209959	5.8680569	-4.3480955
H	1.8999210	4.6872705	-2.4032209

NHC-c-H

E(TPSS-D3/def2-TZVP) = -1950.396493740 (conv)

Lowest Freq. = 15.81 cm^-1

56

Biel-c-H (006c1_H/opt)

C	0.2396401	0.6804376	1.9246498
C	-0.2396401	-0.6804376	1.9246498
N	-0.3993811	-1.0273109	3.2882314
C	0.0000000	0.0000000	4.0427177
N	0.3993811	1.0273109	3.2882314
C	0.8607061	2.3159355	3.8034899
C	-0.8607061	-2.3159355	3.8034899
C	0.4110917	1.4186718	0.7756864

C	-0.4110917	-1.4186718	0.7756864
C	-0.1681317	0.7508668	-0.4834392
S	0.6085917	1.6721985	-1.8910961
C	1.1027928	2.9879158	-0.8353193
C	0.9535847	2.6901018	0.5065964
C	-0.9535847	-2.6901018	0.5065964
C	-1.1027928	-2.9879158	-0.8353193
S	-0.6085917	-1.6721985	-1.8910961
C	0.1681317	-0.7508668	-0.4834392
C	-1.6886387	1.0385544	-0.5152136
C	1.6886387	-1.0385544	-0.5152136
C	-1.6370097	-4.2110242	-1.4088218
C	-1.8953747	-4.3004413	-2.7906771
C	-2.4203200	-5.4623441	-3.3437049
C	-2.6948067	-6.5628582	-2.5301079
C	-2.4376419	-6.4933080	-1.1575485
C	-1.9147500	-5.3339139	-0.6014950
C	1.6370097	4.2110242	-1.4088218
C	1.8953747	4.3004413	-2.7906771
C	2.4203200	5.4623441	-3.3437049
C	2.6948067	6.5628582	-2.5301079
C	2.4376419	6.4933080	-1.1575485
C	1.9147500	5.3339139	-0.6014950
H	0.1986007	3.1075134	3.4450741
H	0.8391937	2.2855098	4.8931831
H	1.8814860	2.5012440	3.4620622
H	-0.1986007	-3.1075134	3.4450741
H	-0.8391937	-2.2855098	4.8931831
H	-1.8814860	-2.5012440	3.4620622
H	1.2842195	3.3798054	1.2729828
H	-1.2842195	-3.3798054	1.2729828
H	-2.1764087	0.5713756	0.3465026
H	-1.8445650	2.1185469	-0.4709530
H	-2.1413874	0.6481723	-1.4278462
H	2.1764087	-0.5713756	0.3465026
H	1.8445650	-2.1185469	-0.4709530
H	2.1413874	-0.6481723	-1.4278462
H	-1.6865294	-3.4480424	-3.4320177
H	-2.6149242	-5.5108053	-4.4104901
H	-3.1022443	-7.4717328	-2.9618052
H	-2.6414566	-7.3501247	-0.5226903
H	-1.7045279	-5.3037616	0.4630517
H	1.6865294	3.4480424	-3.4320177
H	2.6149242	5.5108053	-4.4104901
H	3.1022443	7.4717328	-2.9618052
H	2.6414566	7.3501247	-0.5226903

```
H     1.7045279    5.3037616    0.4630517
H     0.0000000    0.0000000    5.1228916
```

NHI^{Me}

```
E(TPSS-D3/def2-TZVP) = -635.7621381901 (conv)
Lowest Freq. =    35.76 cm^-1
38
IMAM-1e (007c1/opt)
C    -0.2019485   -1.2939601   -0.4505884
C     1.0629305   -0.8138604   -0.5508601
N    -1.0393665   -0.2387795    0.0095571
N     1.0565672    0.5256220   -0.1272749
C    -0.2498000    0.9168694    0.1825543
N    -0.5324543    2.1342476    0.5012673
C    -2.2039125   -0.4718395    0.8950865
C     2.1112928    1.5431975   -0.2373817
C    -1.8917667    2.6275787    0.5561494
H    -1.8744857    3.7013204    0.3353646
H    -2.3514553    2.5236791    1.5538113
H    -2.5654295    2.1488280   -0.1752775
C    -0.7226565   -2.6327180   -0.8516898
C     2.2891473   -1.5208667   -1.0220272
C     3.3974845    1.1468356    0.4932823
H     3.9500245    0.3606362   -0.0292702
H     3.1741510    0.8025380    1.5076016
H     4.0541719    2.0204765    0.5641026
C     2.3516691    1.9534784   -1.6960632
H     3.0532215    2.7939857   -1.7371276
H     1.4105810    2.2673614   -2.1565459
H     2.7738312    1.1337542   -2.2868610
C    -3.4022690   -1.0641201    0.1471615
H    -3.6272692   -0.4695534   -0.7431011
H    -4.2802646   -1.0540827    0.8018938
H    -3.2289244   -2.0981964   -0.1606200
C    -1.8102939   -1.2826269    2.1376876
H    -0.9752725   -0.8008188    2.6552240
H    -1.5126077   -2.3033676    1.8804207
H    -2.6585125   -1.3408601    2.8284597
H     0.0993017   -3.2461316   -1.2289951
H    -1.4727310   -2.5501982   -1.6484027
H    -1.1847974   -3.1830407   -0.0231075
H     2.0180597   -2.5044197   -1.4120122
H     3.0088354   -1.6721206   -0.2088265
H     2.8040365   -0.9758220   -1.8208341
H    -2.4930467    0.5199070    1.2366787
H     1.6639584    2.3970673    0.2805637
```

NHI^{Me}-H

```
E(TPSS-D3/def2-TZVP) = -636.1904988222 (conv)
Lowest Freq. =    45.24 cm^-1
39
IMAM-1e-H (007c1_H/opt)
C    -0.0920694   -1.4552224   -0.2454520
C     1.1071433   -0.8640235   -0.5507924
N    -0.8735876   -0.4925421    0.4019666
N     1.0316571    0.4658629   -0.1154246
C    -0.1781270    0.6672197    0.4652277
N    -0.6457300    1.8132166    1.0652044
C    -2.2216600   -0.6484134    1.0281502
C     2.0266321    1.5585053   -0.3271458
C    -1.6184679    2.6697024    0.3447964
H    -1.1827153    3.1421557   -0.5437852
H    -1.9643894    3.4386455    1.0366717
H    -2.4746805    2.0652154    0.0426106
C    -0.5198683   -2.8622066   -0.4989855
C     2.3097549   -1.4880801   -1.1756236
C     3.3755619    1.2353993    0.3176633
H     3.9125622    0.4573647   -0.2281940
H     3.2523992    0.9180954    1.3570409
H     3.9940388    2.1370497    0.3050299
C     2.1228442    1.9311868   -1.8094194
H     2.7455611    2.8245842   -1.9086837
H     1.1352397    2.1505966   -2.2244296
H     2.5823460    1.1373238   -2.4023679
C    -3.3185660   -0.7756591   -0.0322862
H    -3.2559833    0.0261459   -0.7733901
H    -4.2938617   -0.7146854    0.4584929
H    -3.2694815   -1.7333439   -0.5562634
C    -2.2357573   -1.7825204    2.0545333
H    -1.3973330   -1.6954108    2.7509375
H    -2.2092228   -2.7671624    1.5836188
H    -3.1639517   -1.7149379    2.6287510
H     0.1229829   -3.3050264   -1.2619878
H    -1.5485595   -2.9150171   -0.8610203
H    -0.4418588   -3.4798475    0.4016211
H     2.0311176   -2.4518324   -1.6055480
H     3.0964716   -1.6716220   -0.4362876
H     2.7302775   -0.8761578   -1.9765789
H    -2.3482523    0.2950431    1.5652682
H     1.5940961    2.4124976    0.2010581
H     0.0834371    2.3479006    1.5250236
```

References

[1] V. I. Nikolayenko, D. C. Castell, D. P. Van Heerden, L. J. Barbour, *Angew. Chem. Int. Ed.* **2018**, *57*, 12086.

[2] T. C. Pijper, T. Kudernac, W. R. Browne, B. L. Feringa, *J. Phys. Chem. C* **2013**, *117*, 17623.

[3] Z. Erno, A. M. Asadirad, V. Lemieux, N. R. Branda, *Org. Biomol. Chem.*, **2012**, *10*, 2787.

[4] B. M. Neilson, V. M. Lynch, C. W. Bielawski, *Angew. Chem. Int. Ed.* **2011**, *50*, 10322.

[5] O. V. Dolomanov, L. J. Bourhis, R. J. Gildea, J. A. K. Howard, H. Puschmann, *J Appl Crystallogr* **2009**, *42*, 339.

[6] a) L. Palatinus, S. J. Prathapa, S. van Smaalen, *J Appl Crystallogr* **2012**, *45*, 575; b) L. Palatinus, A. van der Lee, *J Appl Crystallogr* **2008**, *41*, 975; c) L. Palatinus, G. Chapuis, *J Appl Crystallogr* **2007**, *40*, 786.

[7] G. M. Sheldrick, *Acta crystallographica. Section A, Foundations of crystallography* **2008**, *64*, 112.

[8] J. Tao, J. P. Perdew, V. N. Staroverov and G. E. Scuseria, *Phys. Rev. Lett.*, **2003**, *91*, 146401.

[9] a) S. Grimme, J. Antony, S. Ehrlich, H. Krieg, *J. Chem. Phys.*, **2010**, *132*, 154104. b) S. Grimme, S. Ehrlich, L. Goerigk, *J. Comput. Chem.*, **2011**, *32*, 1456.

[10] F. Weigend; R. Ahlrichs. *Phys. Chem. Chem. Phys.*, **2005**, *7*, 3297.

[11] S. Grimme, *Chem. Eur. J.*, **2012**, *18*, 9955.

[12] Y. Zhao, D. G. Truhlar, *J. Phys. Chem. A*, **2005**, *109*, 5656.

[13] a) A. Klamt, *J. Phys. Chem.* **1995**, *99*, 2224-2235. b) A. Klamt, V. Jonas, T. Bürger, J.C. Lohrenz, *J. Phys. Chem. A* **1998**, *102*, 5074-5085. c) F. Eckert, A. Klamt, *AIChE Journal*, **2002**, *48*, 369-385. d) COSMOtherm, Release 19; © **2019** COSMOlogic GmbH & Co. KG, a Dassault Systèmes company.

[14] TURBOMOLE V7.5 **2020**, a development of University of Karlsruhe and Forschungszentrum Karlsruhe GmbH, 1989-2007, TURBOMOLE GmbH, since **2007**; available from http://www.turbomole.com.

[15] Kunetskiy, R. A.; Polyakova, S. M.; Vavřík, J.; Císařová, I.; Saame, J.; Nerut, E. R.; Koppel, I.; Koppel, I. A.; Kütt, A.; Leito, I. et al. *Chem. Eur. J.* **2012**, *18*, 3621.
